# Transcriptional profiling unveils type I and II interferon networks in blood and tissues across diseases

**DOI:** 10.1038/s41467-019-10601-6

**Published:** 2019-06-28

**Authors:** Akul Singhania, Christine M. Graham, Leona Gabryšová, Lúcia Moreira-Teixeira, Evangelos Stavropoulos, Jonathan M. Pitt, Probir Chakravarty, Annika Warnatsch, William J. Branchett, Laura Conejero, Jing-Wen Lin, Sophia Davidson, Mark S. Wilson, Gregory Bancroft, Jean Langhorne, Eva Frickel, Abdul K. Sesay, Simon L. Priestnall, Eleanor Herbert, Marianna Ioannou, Qian Wang, Ian R. Humphreys, Jonathan Dodd, Peter J. M. Openshaw, Katrin D. Mayer-Barber, Dragana Jankovic, Alan Sher, Clare M. Lloyd, Nicole Baldwin, Damien Chaussabel, Venizelos Papayannopoulos, Andreas Wack, Jacques F. Banchereau, Virginia M. Pascual, Anne O’Garra

**Affiliations:** 1 Laboratory of Immunoregulation and Infection, The Francis Crick Institute, London, NW1 1AT UK; 2 Bioinformatics Core, The Francis Crick Institute, London, NW1 1AT UK; 3 Antimicrobial Defence Laboratory, The Francis Crick Institute, London, NW1 1AT UK; 4 Inflammation, Repair and Development Section, National Heart and Lung Institute, Imperial College London, London, SW7 2AZ UK; 5 London School of Hygiene and Tropical Medicine, London, WC1E 7HT UK; 6 Malaria Laboratory, The Francis Crick Institute, London, NW1 1AT UK; 7 Immunoregulation Laboratory, The Francis Crick Institute, London, NW1 1AT UK; 8 Helminth Immunology Laboratory, The Francis Crick Institute, London, NW1 1AT UK; 9 Host-Toxoplasma Interaction Laboratory, The Francis Crick Institute, London, NW1 1AT UK; 10 Advanced Sequencing Facility, The Francis Crick Institute, London, NW1 1AT UK; 11 Department of Pathobiology & Population Sciences, Royal Veterinary College, London, AL9 7TA UK; 12 Division of Infection and Immunity/Systems Immunity University Research Institute, Cardiff University, Cardiff, CF14 4XN UK; 13 Respiratory Infection Section, National Heart and Lung Institute, Imperial College London, London, W2 1PG UK; 14 Inflammation and Innate Immunity Unit, Laboratory of Clinical Immunology and Microbiology, National Institute of Allergy and Infectious Diseases, National Institutes of Health, Bethesda, MD 20892 USA; 15 Immunobiology Section, Laboratory of Parasitic Diseases, National Institute of Allergy and Infectious Diseases, National Institutes of Health, Bethesda, MD 20892 USA; 16 Baylor Institute for Immunology Research, Dallas, TX 75204 USA; 17 Systems Biology and Immunology Department, Sidra Medicine, PO BOX 26999, Doha, Qatar; 18 The Jackson Laboratory for Genomic Medicine, Farmington, CT 06030 USA; 19 Drukier Institute for Children’s Health, Weill Cornell Medical College, New York, NY 10065 USA; 20 National Heart and Lung Institute, Imperial College London, London, W2 1PG UK

**Keywords:** Computational biology and bioinformatics, Gene regulation in immune cells, Infectious diseases

## Abstract

Understanding how immune challenges elicit different responses is critical for diagnosing and deciphering immune regulation. Using a modular strategy to interpret the complex transcriptional host response in mouse models of infection and inflammation, we show a breadth of immune responses in the lung. Lung immune signatures are dominated by either IFN-γ and IFN-inducible, IL-17-induced neutrophil- or allergy-associated gene expression. Type I IFN and IFN-γ-inducible, but not IL-17- or allergy-associated signatures, are preserved in the blood. While IL-17-associated genes identified in lung are detected in blood, the allergy signature is only detectable in blood CD4^+^ effector cells. Type I IFN-inducible genes are abrogated in the absence of IFN-γ signaling and decrease in the absence of IFNAR signaling, both independently contributing to the regulation of granulocyte responses and pathology during *Toxoplasma gondii* infection. Our framework provides an ideal tool for comparative analyses of transcriptional signatures contributing to protection or pathogenesis in disease.

## Abstract (web summary)

The authors present an extensive profile of host transcriptional respones to a diverse group of pathogens and allergens. In doing so, they identify TH1, type I IFN, TH17, and TH2 responses, that underlie each immune response in both the blood and lung, which represents a global profile of host-pathogen immune responses.

## Introduction

The host response during infection and inflammation, in both mouse models and human disease is complex, with a spectrum of responses having been reported across infections with intracellular pathogens, viruses, fungi, or allergy, often driven and dominated by specific groups of cytokines, activating protective responses or pathology^1–6^. There are few transcriptional studies or data resources on the global immune responses spanning different experimental models of diseases across distinct types of immune responses. While tissue transcriptomic approaches have been applied widely to different experimental models of disease individually, this has been reported to a lesser extent for the blood^7,8^. Conversely, in humans, transcriptomic approaches have been applied to whole blood or peripheral blood mononuclear cells (PBMC)^9–16^, however, little is known about how immune responses in blood are reflected at disease sites. Moreover, application of whole blood transcriptomics has not always discerned signatures of disease, revealed only upon transcriptomic analysis of purified cells or PBMC^17,18^.

Many published whole blood disease-specific signatures are dominated by IFN-inducible signatures and those attributable to innate immune responses, as broadly described in both experimental models and human diseases^9–12,15,19^. Although mainly dominated by a type I IFN transcriptional signature, some are accompanied by a cluster of genes that have been attributed to IFN-γ signaling, classically referred to as IFN-stimulated genes (ISGs)^20,21^. Cytokines, chemokines, signaling, and cell membrane molecules can also form part of the IFN signature^22^. How type I IFN and IFN-γ-inducible genes are expressed across a spectrum of different diseases and how the blood transcriptional signature reflects the tissue response are unclear. Effects of type I IFN are clearly not limited to antiviral responses, but also play a role in bacterial^3,4,9,11,23,24^, helminth, allergy^25^, and other inflammatory responses, with beneficial or detrimental effects^3,4,26–33^. IFN-γ is key to activating cell-mediated immune responses to control intracellular pathogens^29,34^, but dampens allergic and anti-helminthic responses^5,34^, and if uncontrolled can lead to immune pathology^35,36^. A blood signature of allergy, asthma, or helminth responses in humans, reflecting T_H_2-type responses or a T_H_17-type^37,38^ signature in inflammation, have as yet not been reported. Whether analysis of such signatures has been attempted and such signatures are not detectable in blood, or as yet have not been investigated, is unclear.

We purposefully chose pathogens and an allergen to yield a wide breadth of different types of immune response in the lung, representative of T_H_1, type I IFN, T_H_17, and T_H_2 responses, hypothesizing that distinct responses underlying the immune response in each model could be determined by the transcriptional signature of unseparated lung cells. To this end, we have used bioinformatics approaches, including modular and cellular deconvolution analyses, to decipher the global transcriptional response in the lungs of mice infected or challenged with a broad spectrum of infectious pathogens, including parasites, bacteria, viruses, fungi, or allergens, and also to determine to what extent each of these responses is preserved in the blood. We demonstrate a unique global transcriptional signature for each of the different diseases against the controls in both lung and blood. The lung transcriptional signatures showed a gradation, ranging from IFN-inducible gene clusters, to those associated with granulocyte/neutrophil/IL-17 dominated genes, to responses dominated by expression of genes encoding T_H_2 cytokines, mast cells and B cells, with only preservation of some signatures in the blood. Unique and overlapping regulatory functions of both type I IFNs and IFN-γ signaling pathways during infection with *Toxoplasma* *gondii*, and a role for both IFNs in regulating the T_H_17/neutrophil-induced pathology, was observed. Our study provides a useful resource of the global differential immune responses in both blood and tissue across a broad spectrum of diseases, also providing translational knowledge on how the blood signature reflects the local tissue immune response. This resource is now easily accessible with the use of an online webapp: https://ogarra.shinyapps.io/MouseModules/.

## Results

### Transcriptional signatures across diseases

**Fig. 1:**
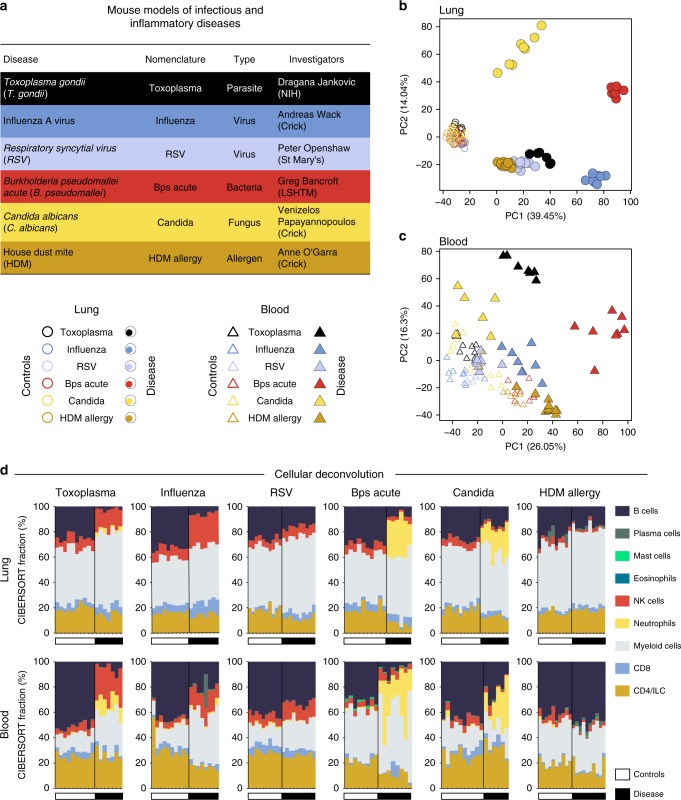
Global transcriptional analysis captures differences across infectious and inflammatory diseases. **a** RNA-seq analysis was performed on lung and blood samples (Supplementary Data 1) obtained from experimental mouse models of 6 infectious and inflammatory diseases. **b**, **c** Principal component analysis in lung (**b**) and blood (**c**) samples, depicting the variation in the global gene expression profiles across diseases. Principal components 1 (PC1) and 2 (PC2), which capture the greatest variation in gene expression, are shown. Circles and triangles represent lung and blood samples, respectively, empty and filled symbols represent control and disease samples, respectively, and color represents mouse models. **d** Stacked bar plots depicting in silico immune cell composition of lung and blood RNA-seq samples, derived using the CIBERSORT algorithm based on cellular signatures obtained from ImmuCC. Each bar represents percent fractions for 9 representative cell types for an individual mouse sample, with colors representing the different cell types. White and black bars at the bottom of each plot represent control and disease samples, respectively. ILC innate lymphoid cells, NK cells natural killer cells

To determine the global changes in the host response to infection and allergens, we performed RNA-based next-generation sequencing (RNA-Seq) on RNA isolated from both lung and blood, at the pre-determined peak of the response of mice infected with *T. gondii*; influenza A virus (influenza); respiratory syncytial virus (RSV); acute *Burkholderia pseudomallei* (*B. pseudomallei*); *Candida albicans (C. albicans);* or challenged with the allergen house dust mite (HDM), to capture the breadth of T_H_1, to type I IFN, to T_H_17, to T_H_2 responses (Fig. 1a; Supplementary Fig. 1a; Supplementary Data 1a). Principal component analysis of the RNA-Seq data depicted a unique global transcriptional signature for each of the different diseases as compared to controls (PC1) in lung (Fig. 1b), and to a lesser extent in blood (Fig. 1c). The total differentially expressed genes in all datasets are shown in Supplementary Data 1b. PC2 representing the second largest variation in the data, separated the different diseases in the lung, with *B. pseudomallei* infected mice positioned in between fungal and other infections (Fig. 1b). Mice infected with RSV and HDM allergy, although distinct from each other, clustered more closely to the controls (Fig. 1b, c). The blood transcriptional signatures were also investigated in a distinct set of mice infected with *Plasmodium chabaudi chabaudi* (*P. chabaudi*, malaria), murine cytomegalovirus (MCMV), *Listeria monocytogenes (Listeria)* and chronic *B. pseudomallei* (Supplementary Fig. 1a), and shown also to cluster away from the controls, with each disease clustering independently of each other, although transcriptional signatures of *P. chabaudi* and MCMV clustered closely to each other (Supplementary Fig. 1b).

To infer the immune cellular composition from transcriptomic data, cellular deconvolution analyses^39,40^ were first applied to the RNA-Seq dataset obtained from the ImmGen Consortium (GSE109125) on flow cytometry sorted cells, to verify the accuracy of the immune subsets being identified from the deconvolution analysis. Based on this comparative analysis, we grouped the 25 immune cell types from the cellular deconvolution analyses^39,40^ into a broader set of 9 categories, representing the major immune cell types (Supplementary Fig. 2a). Application of the validated cellular deconvolution analyses to the lung and blood transcriptional data (Fig. 1a) identified a dominance of diverse cellular populations in the different diseases (Fig. 1d). Natural killer (NK) cells were increased in *T. gondii* and influenza infection in both lung and blood, with only a weaker increase during RSV infection, but were reduced during *B. pseudomallei* and *C. albicans* infections, and remained unaltered in HDM allergy (Fig. 1d). Neutrophils/granulocytes were significantly over-represented in the lungs and blood of mice infected with *B. pseudomallei* and *C. albicans* (Fig. 1d). Cellular deconvolution analysis did not reveal an increase of mast cells or eosinophils in the lungs or blood from HDM-allergen-challenged mice (Fig. 1d). Although accurate mast cell identification by the deconvolution analysis was confirmed using the ImmGen sorted cell dataset, eosinophils could not be verified using this approach since the ImmGen database lacked this population (Supplementary Fig. 2a). To determine that the absence of eosinophil detection was not a limitation of the deconvolution analysis, we analyzed bronchoalveolar lavage cells (BAL) from HDM allergen-challenged mice, since this compartment has previously been reported to contain eosinophils after allergen challenge^41^. Indeed, this confirmed an increase in eosinophils in BAL from HDM allergen-challenged mice (Supplementary Fig. 2b). Overall, these findings demonstrate clear distinctions in the transcriptional signatures and immune cell compositions across a spectrum of infectious and inflammatory diseases, from whole lung and blood.

### Modular transcriptional signatures across diseases

**Fig. 2:**
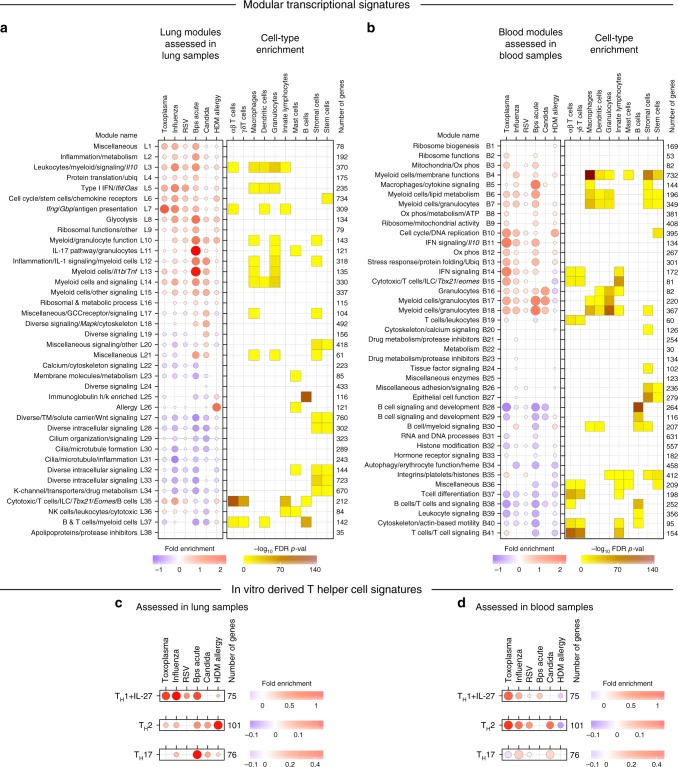
Modular transcriptional signatures define a spectrum of immune responses across diseases. **a**, **b** Fold enrichment in disease compared to controls for modules of co-expressed genes derived using WGCNA in lung (L1–L38) (**a**) and blood (B1–B41) (**b**) samples. Module name indicates biological processes associated with the genes within the module, and number of genes within each module are shown. Fold enrichment scores were derived using QuSAGE, with red and blue circles indicating the cumulative over- or under-abundance of all genes within the module, for each disease compared to the respective controls. Color intensity of the dots represents the degree of perturbation, indicated by the color scale. Size of the dots represents the relative degree of perturbation, with the largest dot representing the highest degree of perturbation within the plot. Within each disease, only modules with FDR *p*-value < 0.05 were considered significant and depicted here. Cell types associated with genes within each module were identified using cell-type-specific signatures obtained for 10 cell types from the ImmGen ULI RNA-seq dataset (Supplementary Fig. 3). Cell-type enrichment was calculated using a hypergeometric test, with only FDR *p*-value < 0.05 considered significant and depicted here. Color intensity represents significance of enrichment. GCC glucocorticoid, K-channel potassium channel, Ox phos oxidative phosphorylation, TM transmembrane, Ubiq ubiquitination. **c**, **d** Fold enrichment for in vitro-derived T helper cell signatures for T_H_1 cells treated with IL-27 (T_H_1 + IL-27), T_H_2 cells, and T_H_17 cells in lung (**c**) and blood (**d**) samples across diseases. Fold enrichment scores were derived using QuSAGE, with red and blue circles indicating the cumulative over- or under-abundance of all genes within the module, for each disease compared to the respective controls. Color intensity of the dots represents the degree of perturbation, indicated by the color scale. Size of the dots represents the relative degree of perturbation, with the largest dot representing the highest degree of perturbation within the plot. Within each disease, only T helper cell signatures with FDR *p*-value < 0.05 were considered significant and depicted here

We next applied Weighted Gene Co-expression Network Analysis (WCGNA)^42^, a modular approach, to identify groups of genes co-expressed together across the lung and blood samples obtained from the various mouse models of infectious and inflammatory diseases. These groups of genes, termed modules, were derived in an unbiased way based on the transcriptional profiles of the protein coding genes across all control and disease samples, resulting in 38 modules in lung (L1–L38, Supplementary Data 2) and 41 modules in blood (B1–B41; Supplementary Data 3). The genes within the modules were functionally annotated using Ingenuity Pathway Analysis (IPA), MetaCore, and Gene Ontology (GO), (Supplementary Data 4 and 5); those commonly identified by the three methods were retained and validated by further manual curation. Next, these modules were assessed in each dataset in lung (Fig. 2a, left panel) and in blood (Fig. 2b, left panel), using QuSAGE^43^ to identify over-abundant (red) and under-abundant (blue) modules in disease datasets against respective controls (Fig. 2a, b, left panels). Cell types associated with each module were identified by comparing cell-type-specific signatures derived for 10 cell types using the ImmGen Ultra Low Input (ULI) RNA-seq dataset (Supplementary Figs. 2a and 3a, Supplementary Data 6) against the genes within each module, using a hypergeometric test (Fig. 2a, b, right panels).

The lung transcriptional signatures showed a gradation, ranging from IFN-inducible gene clusters (IFN-γ/Type II and Type I IFN), to those associated with granulocyte/neutrophil/IL-17 dominated genes, to responses dominated by expression of genes encoding T_H_2 cytokines, mast cells and B cells. Two modules of interferon-related genes were identified—type I IFN inducible genes (Module L5), which included genes associated with innate immune responses, and classical ISGs, such as *Ifnb1*, *Ifit1*and *3, Oas1a, 2* and *3, Oasl1* and *2*, *Mx1, Stat2, Irf7* and *Irf9*; and the IFN-γ-inducible (Type II) gene module (L7), which included *Ifng*, *Irf1*, *2* and *8*, and other downstream targets *Il12rb1 and b2*, *Tap1* and *2, and* genes associated with APC function such as H2-MHC molecules, and host defence, such as *Gbps* (Fig. 2a left panel; Supplementary Data 2). The majority of genes in each respective module (L5 and L7) were attributed to either Type I and/or Type II using the interferome database^44^ (Supplementary Data 2). Both L5 and L7 modules, were over-abundant in lungs from mice infected with *T. gondii*, influenza, RSV, acute *B. pseudomallei*, albeit to different levels, and to a very low extent in HDM allergen-challenged mice. Conversely, in lungs of *C. albican*s infected mice, both L5 and L7 modules were under-abundant. Genes such as *Irf7, Irf9*, *Mx1, Ifit3, Oas1a* (L5) were induced most highly in lungs of virally infected mice (influenza and RSV). (Supplementary Data 2). The type I IFN signaling module (L5) was accompanied by the Leukocytes/Myeloid/Signaling (L3, over-abundant across all diseases) module and both showed enrichment for cell types of the macrophage, dendritic cell and granulocyte lineages, with L3 also being enriched for innate lymphoid cells (ILCs) and αβ T cells (Fig. 2a, right panel). In contrast to the type I IFN-inducible module (L5), the IFN-γ-dominated module (L7) showed cell-type enrichment for αβ T cells, dendritic cells, and ILCs. The lungs from mice infected with *C. albicans*, were dominated by modules encoding myeloid cells (L10, L12–L15), granulocytes (L10, L11) and *Il17* and IL-17-associated cytokines (L11) and *Il1b*/IL-1 signaling (L12, L13, respectively). However, IL-17 (including increased expressions of *Il17a, Il1a and Il22*) and granulocyte-associated (L10 and L11) responses were most pronounced during *B. pseudomallei* infection, with this infection also exhibiting increased IFN gene signatures (L5 and L7) in contrast to the *C. albicans* infection. These IL-17 (L11) and *Il1b*/IL-1 signaling (L12, L13) associated modules, and modules L10 and L14 showed significant enrichment for granulocytes and/or myeloid cells (Fig. 2a, right panel), indicating an increase in these cell types upon infection with *B. pseudomallei* and *C. albicans*, in keeping with the cellular deconvolution analysis (Fig. 1d). The HDM allergen-challenged mice generally exhibited weaker responses in the lung, except for the L26 module containing genes associated with allergic manifestations, which dominated this response (Fig. 2a, left panel). This dominant module (L26) in HDM allergy, contained increased expression of genes such as *Il4, Il5, Il13*, and *Il33*, and the eosinophil-attracting chemokines, *Ccl11* and *Ccl24*, in keeping with the cellular deconvolution analysis (Supplementary Fig. 2b), and genes associated with mast cell function^45^, in keeping with the significant enrichment for mast cells in this module (Fig. 2a, right panel) (Supplementary Data 2). The HDM allergy lung response was also accompanied by an over-abundance of immunoglobulin genes (L25) enriched for B cells (Fig. 2a), which was absent or under-represented across all the other diseases.

Independent modular and cell enrichment analysis in blood also revealed common and reciprocal signatures across diseases (Fig. 2b; Supplementary Data 3 and 5), although the response in the blood appeared weaker than the lung (Fig. 2a, b). IFN-signaling was observed across two modules: B11, containing genes including *Gbps, Ido, Il10, Oas1a* and *Oas1g;* and B14, containing *Ifng*, *Gbps*, *H2*, *Ifits*, *Irf1* and *7, Irgm1* and *2*, *Mx1*, *Oas3*, *Oasl1*, *2*, *Stat1* and *Stat2*, *Tap1* and *2* (Fig. 2b, left panel; Supplementary Data 3). Both modules were over-abundant in blood of mice infected with *T. gondii*, influenza, RSV, acute *B. pseudomallei*, albeit to different levels, with module B14 being enriched for T cells and ILCs, but module B11 showing no enrichment for specific cell types, possibly indicating a broader distribution across many blood cell types (Fig. 2b, right panel). Blood signatures from mice infected with malaria, MCMV, *Listeria*, and chronic *B. pseudomallei*, also demonstrated a strong contribution of both IFN signaling modules (B11 and B14) (Supplementary Fig. 1c). In contrast to the lung, modular derivation directly from the blood did not reveal a detectable *Il17a* and IL-17-associated cytokine gene module, although myeloid cell/granulocyte-associated gene modules (B16–B18) were present in blood of acute *B. pseudomallei*, *C. albicans*, and *T. gondii* and to a lesser extent in influenza and RSV infected mice (Fig. 2b, left panel). This is in keeping with the cellular enrichment for macrophages and granulocytes (Fig. 2b, right panel). Additionally, modular derivation directly from the blood did not reveal a module showing perturbation of allergy-associated genes that had been detected in the lung (Fig. 2a, L26). Modules representing immunoglobulin or B cell-related genes were either unchanged or under-abundant in the blood (B28, B38), except for module B30, which contained B cell and myeloid-associated genes, and was over-abundant during HDM-allergen challenge and influenza infection (Fig. 2b). Under-representation of modules associated with T and some B cell functions was observed, for the most part, in the blood across all diseases (Fig. 2b, left panel; Supplementary Fig. 1c, right panel; B28; and B36–B41), in keeping with previous studies^7,9,46^.

The Cytotoxic/T cells/NK/*Tbx21/Eomes* lung and blood modules (L35 and B15) showed unexpected discordancy from the *Ifng* modules (L7 and B14), with acute *B. pseudomallei* (Fig. 2; Supplementary Fig. 4) giving rise to this apparent discordancy in the modules (Fig. 2a, b; Supplementary Fig. 4; Supplementary Datas 2 and 3). The Cytotoxic/T cells/NK/*Tbx21/Eomes* modules (L35 and B15), were most abundant during infection with *T. gondii* and influenza but significantly under-abundant during *B. pseudomallei* infection (Fig. 2a, left panel), whereas the *Ifng* module, was abundant in all three infections. This disassociation of *tbx21* and *ifng* expression (Supplementary Fig. 4) is in keeping with a previous report^47^.

Overall, these findings, using both RNA-Seq (Fig. 2) and microarray platforms (Supplementary Figs. 5 and 6), demonstrate distinct modular transcriptional patterns in the lungs from the infected/challenged mice reflective of T helper (T_H_)1-type responses (*T. gondii*, influenza, RSV; and more weakly *B. pseudomallei* infection), T_H_17-type (*B.peusodomallei* and *C. albicans* infection) and T_H_2-type (HDM allergy) in vivo (Fig. 2a).

To test whether in vitro-differentiated cells from T_H_1(+IL-27) cells, T_H_2 cells and T_H_17 cells reflect the in vivo responses, we assessed their in vitro-derived T_H_ cell^48^ signatures (Supplementary Data 7) in lung (Fig. 2c) and blood (Fig. 2d) RNA-Seq samples across all diseases. In vitro-derived T_H_1(+IL-27) populations showed enrichment in blood and lungs from *T. gondii*, influenza, RSV, *B. pseudomallei* infected mice; T_H_2 cells showed dominance in the lungs from HDM allergy challenged mice, but not in blood from HDM allergy challenged mice; and T_H_17 cells showed a very strong enrichment in the lungs from *B. pseudomallei* infected mice, with weaker enrichments in *C. albicans*, influenza infected and HDM-allergen-challenged mice, and in blood of *C. albicans* infected mice (Fig. 2d). Collectively this demonstrates that in vitro-derived T_H_ cell subsets express genes reflective of the local in vivo responses to distinct pathogens or allergens, and that each T_H_ cell subset is represented in specific diseases.

### Fidelity of some lung transcriptional profiles in blood

**Fig. 3:**
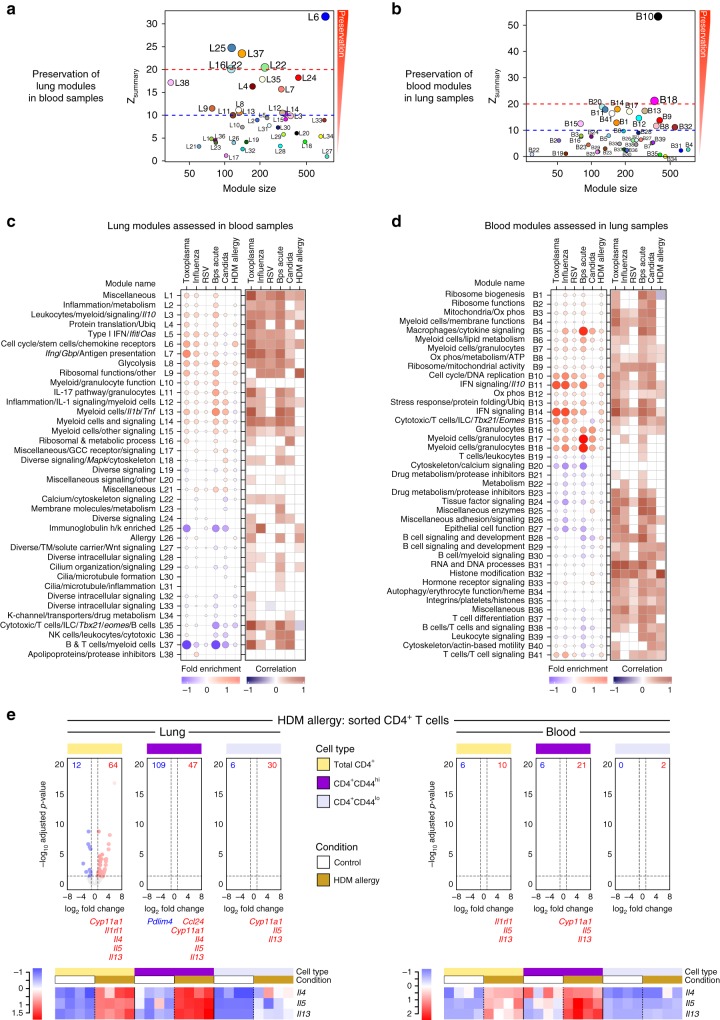
Comparison between the transcriptional profiles in lung and blood across diseases. **a**, **b** Modular preservation to assess the reproducibility and robustness of network topology of the lung modules in blood samples (**a**), and of the blood modules in lung samples (**b**) across the control and disease samples across all mouse models. *Z*_summary_ scores indicative of the degree of preservation were calculated in WGCNA using permutation testing, with scores >10 considered strongly preserved. Each circle represents a module indicated by the module number, with colors assigned in WGCNA for visual distinction. **c**, **d** Assessment of fold enrichment of the lung modules in blood samples (**c**), and of the blood modules in lung samples (**d**) in disease compared to controls. Red and blue circles indicate the cumulative over- or under-abundance of all genes within the module, for each disease compared to the respective controls. Color intensity of the dots represents the degree of perturbation, indicated by the color scale. Size of the dots represents the relative degree of perturbation, with the largest dot representing the highest degree of perturbation within the plot. Within each disease, only modules with FDR *p*-value < 0.05 were considered significant and depicted here. Pearson correlation of foldchanges for genes within the module (disease samples compared to respective controls) between lung and blood is shown, with dark red and blue squares representing positively and negatively correlated gene perturbations, respectively. Significance was calculated using a two-tailed probability of *t*-test values for each correlation, and adjusted for multiple tests within each disease. Only FDR *p*-values < 0.05 were considered significant and depicted here. GCC glucocorticoid, K-channel potassium channel, Ox phos oxidative phosphorylation, TM transmembrane, Ubiq ubiquitination. **e** Volcano plots depicting differential gene expression for all genes, in HDM allergy compared to controls, in sorted CD4 T cells (total CD4^+^, CD4^+^CD44^hi^, and CD4^+^CD44^lo^) from lung and blood samples. Significantly differentially expressed genes (log_2_ fold change >1 or <−1, and FDR *p*-value < 0.05) are represented as red (up-regulated) or blue (down-regulated) dots. The numbers of down- (in blue) or up-regulated (in red) genes are listed in the volcano plot. Below each volcano plot, the genes significantly differentially expressed in the 121 genes in the L26 Allergy module are shown in red (up-regulated) or blue (down-regulated). Heatmaps are shown for log_2_ gene expression values for *Il4*, *Il5*, and *Il13*. Gene expression values were averaged and scaled across the row to indicate the number of standard deviations above (red) or below (blue) the mean, denoted as row *Z*-score

Little has been reported on how different immune responses in blood are reflected at the site of disease. To assess the similarity between the co-expression patterns of genes in lung and blood, the reproducibility and robustness of gene network topology was tested as assessed by *Z*_summary_ scores indicative of the degree of preservation, with scores >10 considered strongly preserved (Fig. 3a, b; Supplementary Data 8 and 9). The cell cycle/DNA processes modules (L6 and B10) were highly preserved across tissues in their co-expression pattern (Fig. 3a, b, Supplementary Fig. 7). The IFN modules were also significantly conserved between lung and blood (L5, L7 and B11, B14) (Fig. 3a–d; Supplementary Fig. 7). The lung granulocyte/myeloid modules (L11–L14) were only moderately preserved in blood (Fig. 3a, c; Supplementary Fig. 7), whereas the equivalent blood modules B17 and B18 granulocyte/myeloid modules were strongly preserved in lung (Fig. 3b, d; Supplementary Fig. 7). Testing the lung modular signature on the blood dataset did reveal that this T_H_17 and granulocyte (L11 and L14) modules were actually preserved in blood (Fig. 3a, c), although the expression of *Il17a* itself was extremely low in the blood (Supplementary Data 2 and 3). The lung allergy module (L26) was not preserved in the blood (Fig. 3a and Supplementary Data 8), in keeping with the inability to detect increased expression of *Il4*, *Il5*, *Il13*, and *Ccl11* (Supplementary Data 2 and 3) and showed weak correlations between the fold changes for genes within this module between lung and blood (Fig. 3c). Overall, the global modular lung signature showed lower correlation in blood samples (Fig. 3c, right panel) than when the blood modular signature was assessed in lung samples (Fig. 3d, right panel). These findings highlight that certain infections, such as *T. gondii* and *B. pseudomallei*, are better preserved between lung and blood than others such as RSV and HDM allergy, and that certain immune responses, such as the IFN response, are better reflected between the lung and blood upon infection, suggesting which immune pathways can become systemic and those which remain local to the insult.

### Lung allergy signatures are not conserved in blood

Since the lung allergy module, including *Il4*, *Il5*, and *Il13* genes, (L26) was not preserved in blood of HDM allergen-challenged mice (Figs. 2a, b and 3a, c), we tested this module in the lungs (BAL) and blood from an alternative nasal sensitization mouse model of allergy to HDM (also, alternative vivarium CML, WB Imperial College)^41^, where similarly the Allergy module L26 was detected in BAL but not blood (Supplementary Fig. 8; Supplementary Data 10). It has been suggested that certain T-cell signatures of disease can only be detected in T cells purified from blood and not whole blood or PBMC^17,18^. To determine whether we could detect any genes from the Allergy Module (L26) in purified CD4^+^ T cells, or activated effector CD4^+^CD44^hi^ versus CD4^+^CD44^lo^ T cells from the blood, RNA-Seq was applied to flow cytometry purified blood populations and compared to the equivalent populations from the lungs of HDM allergen-challenged mice. T_H_2-specific genes were detected at high level in CD4^+^ T cells and activated effector CD4^+^CD44^hi^ from the lungs of HDM-allergen-challenged mice (Fig. 3e; Supplementary Data 11) and now also in the blood of HDM-allergen-challenged mice (Fig. 3e; Supplementary Data 11). These findings are in keeping with the cellular attribution of the T_H_2-type cytokine genes over-expressed within the lung Allergy Module (L26) to αβ T cells and ILCs, with the majority of genes attributable to other cell types in the lung, perhaps explaining the absence of this module in the blood (Supplementary Fig. 9).

### Conservation of lung IFN-inducible signatures in blood

**Fig. 4:**
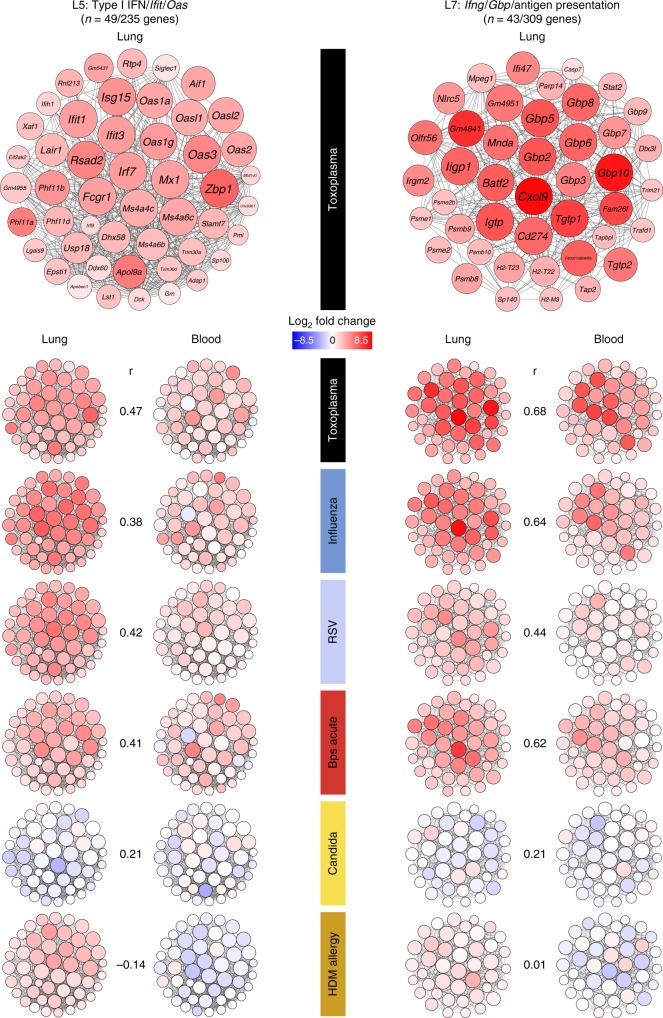
Gene networks of IFN-associated lung modules in lung and blood across diseases. Gene networks for L5 (Type I IFN/*Ifit*/*Oas*) and L7 (*Ifng*/*Gbp*/Antigen presentation) modules depicting the “hub” genes representing genes with high intramodular connectivity, i.e., genes most connected with all other genes within the module. For each module, a representative network is shown for *Toxoplasma* with gene names, followed by smaller gene networks for the 6 diseases. Each gene is represented as a circular node with edges representing correlation between the gene expression profiles of the two respective genes. Color of the node represents log_2_ foldchange of the gene for each disease compared to its respective controls, in the lung (left panel) and blood (right panel) samples for both modules. Pearson correlation coefficients (*r*) for foldchanges for all genes (disease samples compared to respective controls) in the L5 and L7 modules between lung and blood are shown

Since the lung Type I IFN*/Ifit/Oas* (L5) and the *Ifng*/*Gbp*/Antigen presentation (L7) modules were conserved in the blood (Fig. 3a, c; Supplementary Fig. 7), we examined the “hub” genes, i.e., genes most representative of the transcriptional profile of the module and most connected with all other genes within each module, in lung and blood (Supplementary Data 12 and 13; Fig. 4). Over-abundance of both Type I IFN (L5) and IFN-γ (L7) inducible genes was observed in mice infected with *T. gondii*, influenza, RSV, and *B. pseudomallei* infected mice correlating between lung and blood, but not in HDM allergen-challenged or *C. albican*s infected mice. Strikingly, high expression in both modules was observed in the lungs of *T. gondii* infected mice, with all genes within these modules correlating highly across tissues (Fig. 4).

### Type I IFN and IFN-γ signaling in *T. gondii* infection

**Fig. 5:**
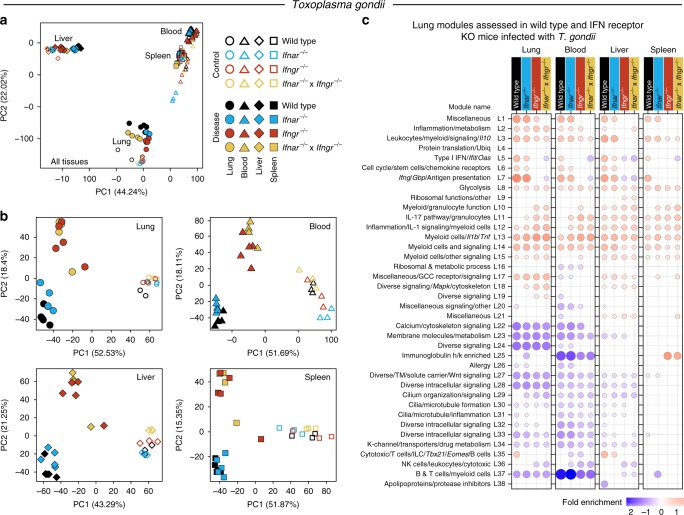
Changes in the transcriptional profiles across tissues following *T. gondii* infection, in the absence of Type I and/or II IFN signaling. **a**, **b** Principal component analysis across all tissues—lung, blood, liver, and spleen (**a**), and individually in each tissue (**b**), depicting the variation in the global gene expression profiles across tissue type, disease, and host genotype. Principal components 1 (PC1) and 2 (PC2), which describe the greatest variation in gene expression, are shown. Shape represents the different tissues, empty and filled symbols represent control and disease samples, respectively, and color represents host genotype: Wild type, *Ifnar*^*−/−*^, *Ifngr*^*−/−*^ and double *Ifnar*^*−/−*^ × *Ifngr*^*−/*−^ mice. **c** Modular transcriptional profiles of all tissues assessed using the lung modules in Wild type and IFN receptor knockout (KO) mice after infection. Red and blue circles indicate the cumulative over- or under-abundance of all genes within the module for each Wild type and IFN receptor KO disease group compared to the respective Wild type controls in each tissue. Color intensity of the dots represents the degree of perturbation, indicated by the color scale. Size of the dots represents the relative degree of perturbation, with the largest dot representing the highest degree of perturbation within the plot. Within each group, only modules with FDR *p*-value < 0.05 were considered significant and depicted here. GCC glucocorticoid, K-channel potassium channel, TM transmembrane, Ubiq ubiquitination

Because of the striking preservation of the type I IFN and IFN-γ pathways in blood and lungs of mice infected with *T. gondii*, we decided to further investigate their role in the global immune response to this pathogen as well as their contribution to disease. To this end, Wild type C57Bl/6, *Ifnar*^*−/−*^*, Ifngr*^*−/−*^ and double *Ifnar*^*−/−*^ × *Ifngr*^*−/−*^, were infected with *T. gondii* and tissues (lung, blood, liver, and spleen) harvested at peak disease for RNA-Seq and histology. PCA plots from the RNA-Seq data showed the biggest separation between tissues, over-and-above the separation observed between uninfected and infected mice, and Wild type versus the different IFN-receptor-deficient mice, with blood and spleen being the most closely associated (Fig. 5a). To reveal within tissue differences, PCA plots were constructed for each tissue separately, where the uninfected mice of all genotypes separated from the *T. gondii* infected mice and accounted for the largest variation in the data (PC1) (Fig. 5b). The second largest variation (PC2) was based on the deficiency of *Ifngr*, upon infection with *T. gondii* (Fig. 5b). To investigate the potential changes in the modular response, which may result from each different IFN-receptor-deficient mice upon infection, we assessed the previously defined modular lung (from Fig. 2a) and blood (from Fig. 2b) signatures across all tissues from these mice, as compared to Wild type uninfected controls for each respective tissue (Fig. 5c).

In *T. gondii* infected *Ifnar*^*−/−*^ mice, decreased modular over-abundance was observed in the Type I IFN module (L5), including lower gene expression for *Ifit, Oas, Irf7* (Supplementary Data 14–18), in the lungs, blood, liver, and spleen, in comparison to the Wild type mice, with the greatest contrast observed in the blood and spleen (Fig. 5c). Strikingly, *Ifngr*^*−/−*^ and *Ifnar*^*−/−*^ × *Ifngr*^*−/−*^ mice, showed a much greater decrease in abundance of Type I IFN (L5)-inducible genes, including classical ISGs, such as, *Ifit, Oas, Irf7*, across all tissues (Fig. 5c; Supplementary Data 14–18). There was also highly decreased abundance of IFN-γ-inducible genes within the (L7) module in the *Ifngr*^*−/−*^ and *Ifnar*^*−/−*^ × *Ifngr*^*−/−*^ mice across all tissues, but in *Ifnar*^*−/−*^ mice this decreased abundance was only prominent in the spleens (Fig. 5c). An under-abundance of the NK cell Module (L36), and the Cytotoxic/T cells/ILC/*Tbx21/Eomes*/B cells (L35) was observed in the blood of *Ifnar*^*−/−*^, *Ifngr*^*−/−*^ and *Ifnar*^*−/−*^ × *Ifngr*^*−/−*^ mice (Fig. 5c). IL-17 pathway/granulocytes (L11), myeloid/granulocyte function (L10) and the inflammation/IL-1 signaling/myeloid Cells (L12) modules were more over-abundant in lungs and blood from *Ifnar*^*−/−*^, *Ifngr*^*−/−*^ and *Ifnar*^*−/−*^ × *Ifngr*^*−/−*^ mice in contrast to the Wild type comparison, with only small changes observed in liver and spleen (Fig. 5c). Additionally, increased abundance of the immunoglobulin module (L25) was observed in the spleen in the absence of IFN-γ signaling (Fig. 5c). Similar findings were observed, when the blood-derived modular signature (from Fig. 2b) was assessed in all tissues from *T. gondii* infected Wild type or IFN-receptor-deficient mice. A decreased modular over-abundance was observed in the equivalent IFN blood modules (B11 and B14) in the *Ifngr*^*−/−*^ and *Ifnar*^*−/−*^ × *Ifngr*^*−/−*^ mice across all tissues (Supplementary Fig. 10). *Ifnar*^*−/−*^ mice, again showed a slight decrease in the IFN-inducible modules (B11 and B14) including genes such as *Oas, Mx1*, as well as a decrease in the cytotoxic/T cells/ILC/*Tbx21/Eomes* module (B15) (Supplementary Fig. 10; Supplementary Data 14–18). In keeping with the results obtained from the lung-derived modules, the blood-derived modules also showed increased abundance, albeit weaker, in the granulocyte (B16) module from the *Ifnar*^*−/−*^, as well as *Ifngr*^*−/−*^ and *Ifnar*^*−/−*^ × *Ifngr*^*−/−*^ mice, being more pronounced in the blood and lung (Supplementary Fig. 10).

Further analysis of our data against the interferome database (Supplementary Datas 2, 3 and 14a, b), revealed that although many modules contain genes that are associated with type I and type II, the most significant enrichment was indeed found in L5 (Type I IFN/ifit/Oas) and L7 (*ifng/Gbp/*Antigen presentation) modules (Supplementary Data 19), in keeping with our annotation. Our data demonstrate partial dependence of genes in the L5 module on type I-inducible signaling, and complete dependence of all the genes in this module on IFNGR signaling during *T. gondii* infection (Fig. 5c). This may be explained by our findings that type I and type II IFNs induce some genes in common (Supplementary Datas 2, 3 and 14), and secondly by our data showing genes classically attributed to type I IFN-inducible signaling by the literature and the interferome data base are actually strongly dependent on IFNGR signaling (Fig. 5c). The module L7 was not affected by abrogation of IFNAR signaling but was completely abrogated in the absence of IFNGR signaling (Fig. 5c), demonstrating their dependence on Type II (IFN-γ) signaling, in keeping with our annotation. A negative role for IFNAR signaling in *T. gondii* infected mice, was observed with increase in the module L11 (IL-17 pathway/granulocytes) (Fig. 5c), although a more profound negative effective was observed in the absence of IFNGR signaling, with increases in modules L10 (myeloid/granulocyte function) and L11 (IL-17 pathway/granulocytes), as well as the L25 (immunoglobulin h/k enriched) module in the spleen only (Fig. 5c).

**Fig. 6:**
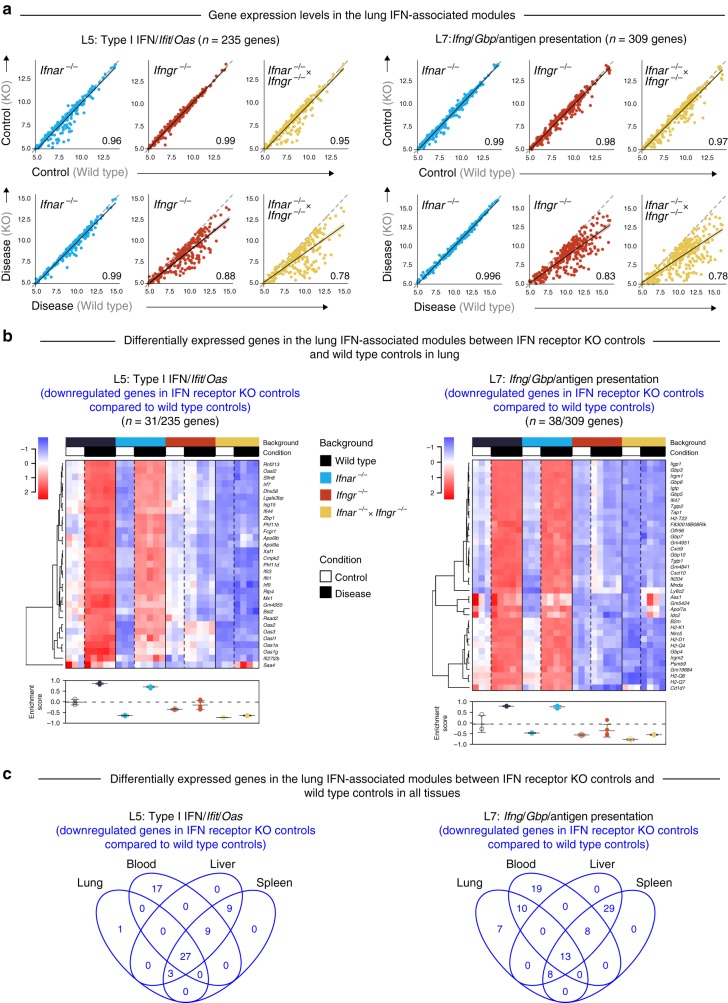
Tonic activity of Type I and/or II IFN signaling in lung within the IFN-associated lung modules. **a** Correlation of gene expression within the L5 (Type I IFN/*Ifit*/*Oas*) and L7 (*Ifng*/*Gbp*/Antigen presentation) modules in lung samples, in control mice: control Wild type group (*x*-axis) compared to each of the control IFN receptor knockout (KO) groups (*y*-axis), and in *T. gondii* infected mice: disease Wild type group (*x*-axis) compared to each of the disease IFN receptor KO groups (*y*-axis). Each dot represents the average log_2_ gene expression value for a gene. Dashed gray line within each plot at the 45° slope represents identical expression levels between Wild type and IFN receptor KO groups, with genes above or below the line showing higher expression in the IFN receptor KO or Wild type group, respectively. Linear regression lines with 95% confidence interval, and Pearson correlation coefficients are shown for each plot. **b** Heatmap depicting the log_2_ gene expression values of the differentially expressed genes between the IFN receptor KO controls compared to the Wild type controls in lung (Supplementary Fig. 11), in the L5 and L7 modules. Gene expression values were averaged and scaled across the row to indicate the number of standard deviations above (red) or below (blue) the mean, denoted as row *Z*-score. Dendrogram shows unsupervised hierarchical clustering of genes, with distances calculated using Pearson correlation and clustered using the complete linkage. Enrichment scores calculated on a single-sample basis using GSVA are shown for the differentially expressed genes below the heatmap for each module. Empty and filled circles represent control and disease samples, respectively, and the color of the circle represents Wild type and IFN receptor KO mice. Mean and standard deviation for each group are shown, with a dashed gray line indicating the mean of the Wild type control group. **c** Venn diagrams depicting the downregulated genes in each of the IFN receptor KO control groups compared to the Wild type control group, in lung (Supplementary Fig. 11), blood (Supplementary Fig. 12), liver (Supplementary Fig. 14), and spleen (Supplementary Fig. 15), depicting commonalities in the list of genes involved in tonic IFN signaling across tissues in the L5 and L7 modules

Type I IFNs have been reported to be constitutively produced at low quantities in the absence of infectious insult, and yet exert profound effects^49^, with different sets of genes being affected under tonic or Type I IFN-stimulated conditions^22^. To investigate further a change in Type I IFN induced tonic signaling, we examined global and modular effects of the uninfected *Ifnar*^*−/−*^, *Ifngr*^*−/−*^ and double *Ifnar*^*−/−*^ × *Ifngr*^*−/−*^ mice. Indeed, within the Type I IFN/Ifit/Oas module (L5), lungs from uninfected *Ifnar*^*−/−*^ mice showed decreased expression as compared to Wild type controls for a subset of genes, which included *Ifit1, Ifit3, Oas1a, Oas2, Oas3, Irf7, Irf9*, and *Mx1* (Fig. 6a, b; Supplementary Fig. 11; Supplementary Data 15). Upon *T. gondii* infection, these genes were upregulated in the lungs of *Ifnar*^*−/−*^ mice, similarly to the Wild type mice, however to a lesser extent (Fig. 6b), in keeping with the modular analysis (Fig. 5c). These data suggest that other signaling pathways induced during infection can compensate for type I IFN in the induction of these genes. Strikingly, the upregulation of these type I IFN-inducible genes during infection was totally abrogated in the lungs of the *Ifngr*^*−/−*^ and *Ifnar*^*−/−*^ × *Ifngr*^*−/−*^ mice, demonstrating the dependence of these genes, reported to be induced by type I IFN, on IFN-γ signaling during *T. gondii* infection (Fig. 6b). In the *Ifng*/Gbp/antigen presentation module (L7), decreased expression of a subset of genes, including the known IFN-γ-induced genes such as *Gbps, Tap1*, and MHC molecules, was observed in the lungs of uninfected *Ifngr*^*−/−*^ mice and double *Ifnar*^*−/−*^ × *Ifngr*^*−/−*^ mice (Fig. 6a, b; Supplementary Fig. 11; Supplementary Data 15). Even upon *T. gondii* infection, these tonically regulated genes by IFN-γ, were not increased in the lungs of *Ifngr*^*−/−*^ and *Ifnar*^*−/−*^ × *Ifngr*^*−/−*^ mice (Fig. 6a, b). Collectively these data demonstrate genes involved in the tonic type I IFN and IFN-γ signaling in lungs (Fig. 6a, b; Supplementary Fig. 11; Supplementary Data 15). Similar findings were observed in the blood, liver and spleen of uninfected *Ifnar*^*−/−*^*, Ifngr*^*−/−*^ and double *Ifnar*^*−/−*^ × *Ifngr*^*−/−*^ versus Wild type uninfected mice (Supplementary Figs. 12–17; Supplementary Datas 16–18).

**Fig. 7:**
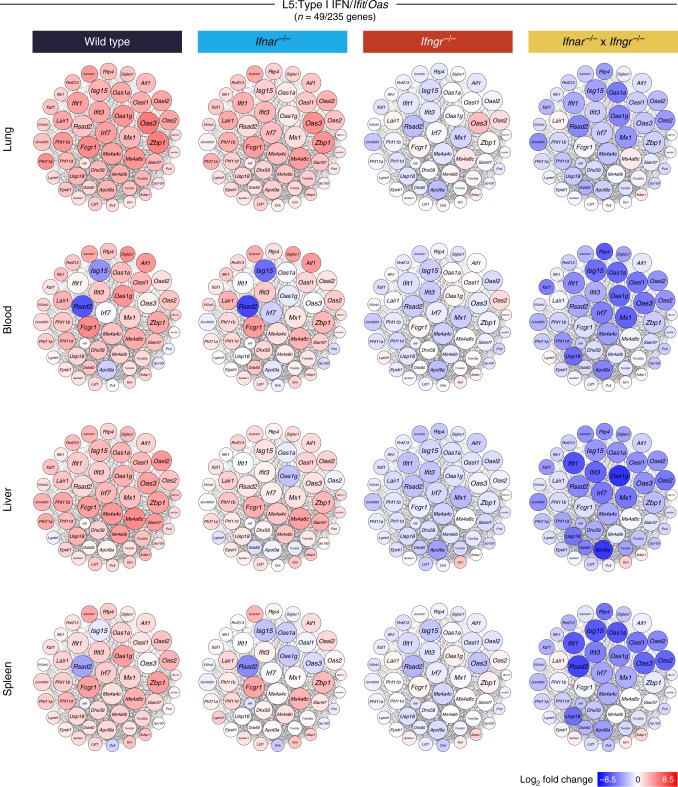
Type I and/or II IFN signaling regulates the expression of genes within the lung Type I IFN//*Ifit*/*Oas* (L5) module following *T. gondii* infection. Gene networks depicting the “hub” genes in the lung L5 module representing genes with high intramodular connectivity, i.e., genes most connected with all other genes within the module. Each gene is represented as a circular node with edges representing correlation between the gene expression profiles of the two respective genes. Color of the node represents log_2_ foldchange of the gene for Wild type or IFN receptor knockout *T. gondii* infected mice compared to Wild type controls, across lung, blood, liver, and spleen

The absence of type I IFN signaling in the *Ifnar*^*−/−*^ mice showed a robust but not absolute decrease in the induction of the hub genes in the Type I IFN/*Ifit*/*Oas* module (L5), across all tissues, with the greatest decrease observed in the liver and spleen upon *T. gondii* infection (Fig. 7). The genes in this network were abrogated in the absence of IFN-γ signaling during *T. gondii* infection in *Ifngr*^*−/−*^ mice (Fig. 7), similarly to findings reported above (Fig. 6). The biggest decrease in genes in this network of the L5 module was observed in the double *Ifnar*^*−/−*^ × *Ifngr*^*−/−*^ mice, confirming the requirement for both Type I IFN and IFN-γ signaling in the induction of type I IFN-inducible genes (Fig. 7). Analysis of the network of hub genes in the IFN-γ inducible genes module (L7) demonstrated an absolute requirement for IFN-γ signaling but not type I IFN signaling for the induction of these genes upon *T. gondii* infection (Supplementary Fig. 18).

### Type I IFN and IFN-γ control pathology during infection

**Fig. 8:**
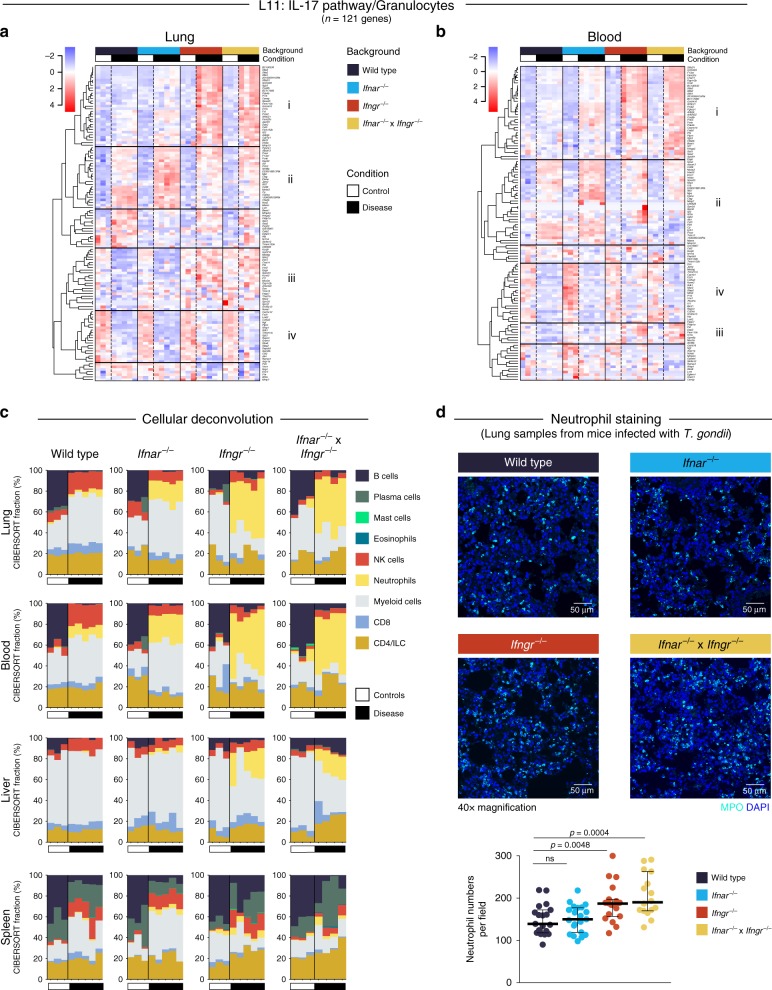
Type I and/or II IFN signaling differentially regulates granulocyte-associated genes and neutrophil recruitment during *T. gondii* infection. **a**, **b** Heatmaps depicting the log_2_ expression values of all genes within the L11 (IL-17 pathway/granulocytes) lung module in lung (**a**) and blood (**b**) samples, across Wild type and IFN receptor knockout (KO) mice, and control and disease samples. Gene expression values were averaged and scaled across the row to indicate the number of standard deviations above (red) or below (blue) the mean, denoted as row *Z*-score. Dendrograms show unsupervised hierarchical clustering of genes, with distances calculated using Pearson correlation and clustered using the complete linkage. Four clusters of genes within the heatmaps are highlighted with roman numerals, showing distinct expression patterns across the groups in lung and blood samples. **c** Stacked bar plots depicting in silico immune cell composition of lung, blood, liver, and spleen RNA-seq samples, derived using the CIBERSORT algorithm based on cellular signatures obtained from ImmuCC. Each bar represents percent fractions for 9 representative cell types for an individual mouse sample, with colors representing the different cell types. White and black bars at the bottom of each plot represent control and disease samples, respectively. ILC innate lymphoid cells, NK cells natural killer cells. **d** Representative immunofluorescence confocal micrographs of thick lung sections depicting MPO-positive neutrophils (MPO, cyan) in Wild type and IFN receptor KO mice upon *T. gondii* infection. Scale bar represents 50 μm. Quantification of neutrophil numbers per field is shown, with each dot representing one field from one mouse (*n* = 4–5 fields from *n* = 4–5 mice per group), with median and 95% confidence interval indicated. Significance was calculated using unpaired *t*-test for each IFN receptor KO compared to Wild type; ns not significant

Consistent with differences at the transcriptional level, major decreases in weight loss were observed during *T. gondii* infection in all IFN-receptor-deficient mice, as compared to the infected Wild type mice (data not shown). Since the *Ifnar*^*−/−*^ mice also showed signs of increased pathology upon *T. gondii* infection despite the rescue, in part, of Type I IFN-inducible response in these mice upon *T. gondii* infection (Figs. 5c, 6 and 7), we investigated the modular response further to understand the mechanism underpinning the exacerbated disease in these mice. An increase in certain neutrophil-associated genes from the IL-17 pathway/granulocytes module (L11), was observed in all IFN-receptor-deficient mice upon infection (Cluster i and ii, Fig. 8a, b), although *ll17a* itself was not detectable in *T. gondii* infected tissues, except in the absence of IFN-γ signaling (Supplementary Data 14). In keeping with this, *Il1b*, contained in Module L13, was also elevated in blood and other tissues of *Ifnar*^*−/−*^ mice (Fig. 5c; Supplementary Data 14–18). On the other hand, increased expression of genes in Cluster iii, such as *Pf4* (reported as a chemokine for neutrophils and monocytes) were only increased in the lungs of *Ifngr*^*−/−*^ and *Ifnar*^*−/−*^ × *Ifngr*^*−/−*^ *T. gondii* infected mice (Fig. 8a). Additional to the observed increase of neutrophil-associated genes (Figs. 5c and 8a, b), we additionally show an increase in the proportion of granulocytes/neutrophils in the lungs and blood of all *Ifnar*^*−/−*^, *Ifngr*^*−/−*^ and *Ifnar*^*−/−*^ × *Ifngr*^*−/−*^ mice as compared to Wild type infected with *T. gondii*, using cellular deconvolution analyses of our RNA-Seq data (Fig. 8c).

To verify differences observed at the transcriptional level in *Ifnar*^*−/−*^*, Ifngr*^*−/−*^, and double *Ifnar*^*−/−*^ × *Ifngr*^*−/−*^ as compared to Wild type mice infected with *T. gondii*, we examined the mice using histopathology. In the lungs of infected Wild type mice, mild histiocytic and neutrophilic interstitial pneumonia with mild necrosis of alveolar walls was generally detected (Supplementary Fig. 19a and b). In the lungs of all of the infected IFN-receptor-deficient mice, interstitial pneumonia ranged from mild to moderate with frequently increased numbers of granulocytes (neutrophils and eosinophils) and/or mononuclear cells (Supplementary Fig. 19a and b). The livers of *T. gondii* infected Wild type mice presented with multifocal random necrotizing and pyogranulomatous hepatitis. Infected *Ifngr*^*−/−*^ additionally contained frequent foci of hepatocellular lytic necrosis, whereas *Ifnar*^*−/−*^ infected mice presented with multiple large thrombi occluding vessels with associated hepatocellular coagulative necrosis, resulting from ischemic injury, both of which could be associated with the increased neutrophilic activity observed (Supplementary Fig. 19a). Increased numbers of granulocytes were found in the absence of IFNR signaling in all tissues analyzed (lung, liver, spleen) from *T. gondii* infected mice (Supplementary Fig. 19a and b). This was confirmed by an increase in neutrophil numbers in the lungs of *T. gondii* infected mice, as shown by staining of myeloperoxidase (MPO), quantified by the number of positive cells for nuclear morphology, with the greatest effect seen in the absence of IFNGR signaling (Fig. 8d). Increased parasite loads, inferred by parasite RNA-readcounts were observed especially in blood and liver in the absence of both IFNAR or IFNGR signaling, although this was more pronounced in the *Ifngr*^*−/−*^ mice (Supplementary Fig. 19c), in keeping with our observed decrease in *Nos2* gene expression (Supplementary Data 14). It is probable that in the absence of IFNR signaling macrophages are unable to control the infection which results in increased neutrophil recruitment, in keeping with increased neutrophil levels seen in blood and lung by deconvolution analysis (Fig. 8c) and granulocyte-associated genes (Fig. 5c; Supplementary Fig. 10).

It should be noted that the wild type control mice infected with *T. gondii* either at NIAID, NIH, or The Francis Crick Institute, showed a similar modular lung and blood transcriptional signature demonstrating the robust nature of this global signature of infection, generated from a large number of high-quality samples (Supplementary Data 20) across different vivariums (Supplementary Fig. 20). Collectively, our transcriptome and histology data show that type I IFN and IFN-γ signaling are both involved in control of neutrophilic inflammation during *T. gondii* infection, likely contributing to the increased pathology seen in the *Ifnar*^*−/−*^*, Ifngr*^*−/−*^, and double *Ifnar*^*−/−*^ × *Ifngr*^*−/−*^ as compared to Wild type mice upon infection, over-and-above any type I IFN and IFN-γ induced pathways of microbial control.

## Discussion

We have generated a comprehensive resource of modular transcriptional signatures from various infectious and inflammatory diseases to identify commonalities and differences in the immune response to specific infections or challenges to aid the discovery of pathways in disease. We show that distinct immune responses in experimental models of T_H_1, type I IFN, T_H_17, and T_H_2 diseases could be determined by the whole transcriptional signature of unseparated lung cells. Type I IFN and IFN-γ pathways showed high expression in lungs of *T. gondii*, influenza A and RSV infected mice. Pathways driven by IL-17 including granulocyte/neutrophil-associated genes were abundant in the lungs of mice infected with *B. pseudomallei* and *C. albicans* only, and a signature of mast cells and T_H_2-type cytokines was only abundant in the lungs of mice challenged with HDM allergen. IFN-inducible gene signatures were detectable in the blood-derived modules, similarly across the different diseases, with strongest preservation in *T. gondii* infected mice, but the IL-17 pathway module was not detectable. Testing the lung module in blood, revealed some IL-17 pathway genes, but these did not include *Il17* family members. The T_H_2 allergy module was not preserved in the blood of mice challenged with HDM allergen, but only detectable in purified blood CD4^+^ effector T cells.

Our obervations that T_H_2-associated genes were only detectable in purified blood CD4^+^ effector T cells are similar to reported findings that certain disease-specific signatures were only detectable in T cells purified from blood and not from whole blood or PBMC^17,18^. Collectively these findings may suggest that disease-specific signatures attributable to T cells, or other cells of low abundance in the blood, may not be revealed in whole blood or PBMC, but will require cell purification. However, a T_H_2-associated gene signature can be found in whole lung where the immune response is generated to the allergen and there are sufficient effector T cell numbers. In contrast to the difficulty in detecting T_H_2 allergy and T_H_17 signatures in the blood, our findings suggest that cells contributing to IFN-inducible signatures are more abundant in the blood, in keeping with the numerous reports that IFN-inducible signatures are detectable in whole blood in human disease^6,9–16,19^.

Type I IFN-inducible genes normally reflective of viral infections^16,19^ were herein additionally observed in the lungs and blood of bacterial infections with *B. pseudomallei* as previously reported during tuberculosis disease^9,11,12,15,46^, and now also to a very high extent during *T. gondii* infection, on a par with influenza infection. For example, we observe genes such as Oas, Mx1, Ifit, as well as those not regarded as classic IFN-inducible genes, including cytokines, chemokines, signaling and cell membrane molecules to form part of the IFN signature^22^. Accompanying this response, were IFN-γ inducible genes such as Irf8, fundamental for an IL-12-driven T_H_1 response^29^, and molecules involved in protection against intracellular bacteria and parasites such as Gbps and Nos2. The co-existence of IFN-γ and T_H_17 responses during B. pseudomallei infection which we observed, demonstrate the complex immune response to this pathogen, where IFN-γ is essential for protection^50^ yet the response is accompanied by increased granulocyte/neutrophils. The absence of type I IFN and IFN-γ-inducible signatures during *C. albicans* infection, could indicate a lack of induction of the signaling pathways required to induce these molecules, or active inhibition by cytokines or other regulatory factors. For example inhibition of type I IFN by IL-1 as has been reported during *Mycobacterium* *tuberculosis* infection^51^.

Type I IFN blocks IFN-γ mediated protective responses to intracellular pathogens^4,51–55^, and yet a number of pathogens elicit both type I IFN-induced gene expression and IFN-γ responses^3,4,9^. However, it is unclear how type I IFN and IFN-γ-inducible gene pathways co-operate or regulate each other, especially when expressed to a very high level as we now show during *T. gondii* infection in vivo. To examine this relationship between type I IFN and IFN-γ-mediated regulation of gene expression and function we infected *Ifnar*^*−/−*^*, Ifngr*^*−/−*^ and *Ifnar*^*−/−*^ × *Ifngr*^*−/−*^ mice with *T. gondii*. Classic IFN-γ-induced genes encoding APC processing and presentation function, such as MHC molecules and *Tap1* and *2*, found in lung module L7, and *Nos2*, were all abrogated in the absence of IFNGR signaling, reinforcing IFN-γ as the driver of this modular response. We demonstrate that induction of genes such as *Oas, Mx1, Ifit* reported to be regulated by type I IFN signaling, is totally dependent on IFN-γ signaling during infection with *T. gondii*. This may suggest a stronger contribution of IFN-γ signaling for the induction of genes known to be induced by type I IFN, in a setting such as *T. gondii* infection, where *Ifng* levels are at least three-fold higher in lungs of mice infected with *T. gondii* as compared to influenza. The expression of IFN-γ induced *Nos2* was increased in livers and spleens of *Ifnar*^*−/−*^ mice infected with *T. gondii*, but this increase was abrogated when IFN-γ signaling was absent in *Ifnar*^*−/−*^ × *Ifngr*^*−/−*^ mice as seen in *Ifngr*^*−/−*^ mice. This demonstrates in vivo blockade of IFNGR signaling by type I IFN during a parasite infection, as has been reported for infections with bacteria such as *L. monocytogenes*^52^ or *M. tuberculosis*^53,54,56^. Our findings collectively show the complexity of the relationship between type I IFN and IFN-γ signaling. Although as previously reported type I IFN can indeed negatively regulate IFN-γ-induced genes, type I IFN-induced gene expression appears to be partially dependent on IFNAR signaling, while totally dependent on IFN-γ signaling during infection with *T. gondii*.

While IFN-γ has been shown to be a dominant cytokine in protection against *T. gondii* infection^29,57^, type I IFN has also been shown to offer protective effects^58,59^, with both of these IFNs exhibiting microbicidal effects in vitro^60^ against *T. gondii* infection. Mechanisms of protection by IFN-γ against *T. gondi* infection have been broadly described^61^, but it is unclear how type I IFN controls disease. Supportive of a role for type I IFN in protection against *T. gondii* infection, enhancement of NK cell IFN-γ production by type I IFN has been reported^62^. TLR-12 signaling promoting type I IFN production by plasmacytoid dendritic cells, has also been shown to increase IL-12 production^63^. In both cases type I IFN would therefore augment T_H_1 immunity, fundamental for protection against *T. gondii* infection^29^. Here we show that the absence of type I IFN results in increased parasite loads in the blood and liver of *T. gondii* infected mice accompanied by exacerbated pathology. This correlated with increased transcriptional modules encoding granulocyte-related genes and higher neutrophil numbers. Although neutrophils have been shown to dampen *T. gondii* infection, uncontrolled and very high numbers of neutrophils may contribute to inflammation, further exacerbating pathology and pathogen load, such as has been reported during *M. tuberculosis* infections^64–67^.

Tonic activity of the type I IFN signaling pathway has been reported in the absence of infection^8,22,49^, which we also show here, although we additionally show that these genes, including classical ISGs, are highly upregulated during infection with *T. gondii*. We show here the findings that IFN-γ can also mediate a tonic effect on a small number of genes, such as *Gbps, Cxcl9* and genes of the MHC-complex, distinct from ISGs associated with type I IFN signaling. These IFN-γ-induced tonic genes were additionally upregulated upon infection with *T. gondii*, and are totally dependent on IFNGR signaling. The observed tonic IFN-γ signaling may further impact the immune response to pathogens.

In conclusion, using transcriptomic analyses of comprehensive datasets generated from in vivo models of infection and inflammation, we have captured a breadth of distinct immune responses. This spectrum consisted of distinct immune response patterns in the lung, ranging from very high IFN-γ expression, type I IFN-inducible gene expression, IL-17-induced neutrophil dominated signatures and expression of *Il4, Il5, Il13*, and mast cell-associated genes. Type I IFN and IFN-γ signatures were found to be abundant across all the diseases, albeit to different levels, except during *C. albicans* infection. IFN-inducible signatures were preserved in blood, with strongest co-expression during *T. gondii* infection. Our unbiased transcriptomic analyses further revealed that although genes known to be inducible by type I IFN were decreased in the absence of type I IFN signaling as expected, they were completely abrogated in the absence of IFN-γ signaling, revealing an advanced layer of regulation in an IFN-γ-rich environment resulting from *T. gondii* infection. Additionally, both type I IFN and IFN-γ signaling were shown to each play a major role in the regulation of granulocyte responses and the control of parasite load and pathology during *T. gondii* infection. These findings, using transcriptomic analyses of blood and whole organ tissue to capture cellular interactions contributing to changes in gene expression during infection and disease outcome, together with mice deficient in IFNAR and IFNGR signaling, provide a framework for discovery of pathways of gene regulation in disease.

## Methods

### Experimental animals

All mice were bred and maintained in specific pathogen-free conditions according to Home Office UK Animals (Scientific Procedures) Act 1986 unless otherwise stated and were used at 6–18 weeks of age. C57BL/6J Wild-type mice were bred at the MRC National Institute for Medical Research (NIMR) or The Francis Crick Institute unless otherwise stated. *Ifnar*^*−/−*^ originally provided by Matthew Albert (Institute Pasteur, France)^33^ and *Ifngr*^*−/−*^^68^ on the C57BL/6 background were further inter-crossed to generate double *Ifnar*^*−/−*^ × *Ifngr*^*−/−*^ mice. All animal experiments were carried out in accordance with UK Home Office regulations unless otherwise stated, project licences: *T. gondii* infection of wild type control mice, *Ifnar*^*−/−*^, *Ifngr*^*−/−*^ and double *Ifnar*^*−/−*^ × *Ifngr*^*−/−*^ mice (for Figs. 5–8 and Supplementary Figs. 10–19) 80/2616 (at The Francis Crick Institute); Influenza A infection, 70/7643 (MRC NIMR); RSV infection, 70/7554 (Imperial College London); *B. pseudomallei* infection, 70/6934 (London School of Hygiene and Tropical Medicine); *C. albicans* infection, 70/8811 (MRC NIMR); HDM allergy, 70/7643 (MRC NIMR), P5AF488B4 (The Francis Crick Institute) and 70/7463 (Imperial College London); *Aspergillus fumigatus* infection, 70/8811 (The Francis Crick Institute); *P. chabaudi* AS infection, 80/2358 (MRC NIMR) and 70/8326 (The Francis Crick Institute); MCMV infection, 30/2969 (Cardiff University); *L. monocytogenes* infection, 70/7643 (MRC NIRM) and were approved by the institutions’ Ethical Review Panels unless otherwise stated. C57BL/6 mice mice for *T. gondii* infection used for module derivation and further analysis (Figs. 1–4 and Supplementary Figs. 1, 4–7) were maintained and infected at an American Association for the Accreditation of Laboratory Animal Care-accredited animal facility at National Institute of Allergy and Infectious Diseases (NIAID) and housed in accordance with the procedures outlined in the Guide for the Care and Use of Laboratory Animals under an animal study proposal approved by the NIAID Animal Care and Use Committee.

### Disease models

For ***T. gondii*** **infection**, type II avirulent *T. gondii* strain ME-49 cysts were obtained from brains of chronically infected C57BL/6 mice (Taconic Biosciences, USA). Cyst preparations were pepsin treated to eliminate potential contamination with host cells and female C57BL/6 mice were inoculated intraperitoneally (i.p.) with an average of 15 cysts in phosphate buffered (PBS) as described^69^. On day 7 post infection, blood and lung samples were collected from individual mice using uninfected C57BL/6 mice as controls. T. gondii infection was similarly carried out of (1) C57BL/6 wild type mice at the NIAID, NIH, (for Figs. 1–4 and Supplementary Figs. 1, 4–7) and (2) C57BL/6 wild type control mice, Ifnar, *Ifngr*^*−/−*^ and double *Ifnar*^*−/−*^ × *Ifngr*^*−/−*^ mice (Figs. 5–8 and Supplementary Figs. 10–19) at The Francis Crick Institute, with blood, spleen, liver, and lung samples harvested at days 6/7 from infected mice. Whole blood was mixed with Tempus reagent (Life Technologies) at 1:2 ratio prior to freezing, lung samples were stored in RNA-later (Ambion) at −80 °C until RNA isolation.

For **Influenza A virus infection**, Influenza A/X-31 (H3N2) strain (a kind gift from Dr. J. Skehel, MRC NIMR) was grown in the allantoic cavity of 10-day-embryonated hen’s eggs, stored at −80 °C and titrated on Madin-Darby Canine Kidney (MDCK) cells prior to infection. Female C57BL/6J mice (MRC NIMR) were infected intranasally (i.n.) with 8 × 10^3^ TCID_50_ in 30 μl of PBS. Control uninfected mice received PBS only. Whole blood and lung samples were collected from individual infected and control treated mice on day 6 post infection.

For **RSV infection**, plaque-purified human RSV A2 strain originally obtained from the American Type Culture Collection (ATCC) was grown to high titer (≥10^7^ focus-forming units (FFU) per ml) in Hep-2 cells, snap frozen, and assayed for infectivity prior to use. All virus preparations were free of mycoplasma (Gen-Probe, San Diego, CA). Female C57BL/6J mice (MRC NIMR) were infected i.n. with 1 × 10^6^ FFU of RSV diluted in 100 μl PBS. Control uninfected mice received PBS only. Whole blood and lung samples were collected from individual mice on day 2 post infection.

For ***B. pseudomallei*** **infection**, *B. pseudomallei* strain 576 originally isolated from a melioidosis patient was provided by Dr. T. Pitt (Health Protection Agency, London, UK) and cryopreserved as described^7^. All procedures using live bacteria were performed under Advisory Committee on Dangerous Pathogens containment level 3 conditions. Female C57BL/6 mice (Harlan Laboratories, UK) were infected intranasally with 50 μl containing 2500 colony forming units (CFU) (acute model) or 100 CFU (chronic model)^70^ of *B. pseudomallei* derived from cryopreserved stocks diluted in pyrogen-free saline. Control uninfected mice received 50 μl pyrogen-free saline only. Whole blood and lung samples were collected from individual infected and control treated mice on day 3 post infection (acute model) and on days 27, 39, 49, 65, and 90 (chronic model).

For ***C. albicans*** **infection**, *C. albicans* (clinical isolate SC5314, a kind gift from A. Zychlinsky, Max Planck Institute for Infection Biology, Berlin Germany) was cultured in yeast extract peptone dextrose medium at 37 °C overnight, subcultured for a further 4 h and resuspended in PBS immediately prior to infection. Female C57BL/6J mice (MRC NIMR) were infected intratracheally (i.t.) with 1 × 10^5^ *C. albicans* diluted in 50 μl PBS. Whole blood and lung samples were collected from individual mice on day 1 post infection, using uninfected C57BL/6J mice as controls.

For **HDM allergy induction**, female C57BL/6J mice (MRC NIMR) were sensitized with 10 mg HDM (Greer) and 2 mg Imject Alum (Thermo Scientific) in 200 μl PBS or Alum alone as control by i.p. injections on days 0 and 14, followed by i.t. challenge with 10 mg HDM in 20 μl of PBS or PBS on days 21 and 24 as described^71^. Whole blood and lung samples were collected from individual HDM and PBS control treated mice on day 25. For fluorescence-activated cell sorting (FACS) of T cells, pooled blood from four or five individual HDM or PBS-treated mice was collected into heparin sodium (Wockhardt) at 10–30 international units per ml of blood. PBMCs were isolated by density separation with Lympholyte®-Mammal (Cedarlane). Lung CD4^+^ T cells were enriched by positive selection (Miltenyi Biotech) from a corresponding pool of lungs. Blood and lung cells were stained with CD3 (145-2C11) APC, CD4 (RM4-5) eFluor450 and CD44 (IM7) PE (all from eBioscience) and CD3^+^CD4^+^, CD3^+^CD4^+^CD44^low^ and CD44^high^ cells were sorted on MoFlo XDP (Beckman Coulter) and BD FACSAria™ Fusion (Beckton Dickinson) flow cytometers and 15,000 per population collected into TRI-Reagent LS (Sigma-Aldrich). Alternatively, for HDM allergy induction via mucosal exposure (test HDM allergy dataset), female C57BL/6J mice (Charles River Laboratories) were administered 25 μg HDM (Greer) in 25 μl PBS or 25 μl PBS control i.n. under isoflurane anesthesia 5 days per week for 3 weeks. Whole blood and BAL samples were collected from individual HDM and PBS control treated mice 24 h after final allergen challenge. For BAL, airways were flushed 3 times with 1 ml of chilled 5 mM EDTA (Invitrogen, Thermo Fisher) in PBS via a tracheal cannula, before resting mice with 1 ml EDTA/PBS in the lungs for 5 min and lavaging a further 3 times with 1 ml EDTA/PBS.

For ***P. chabaudi*** **AS infection**, the cloned *P. chabaudi* AS line was originally obtained from David Walliker, University of Edinburgh, UK and subsequently cryopreserved as described^72^. Female C57BL/6J (MRC NIMR) mice housed under reverse light conditions (light 19.00–07.00, dark 07.00–19.00 GMT) were infected by i.p. injection of 10^5^ infected red blood cells derived from cryopreserved stocks. Whole blood samples were collected from individual mice on day 6 post infection, using uninfected C57BL/6J mice as controls.

For **mCMV infection**, Smith strain mCMV originally obtained from the ATCC was prepared in salivary glands from BALB/c mice (Harlan, UK) and purified over a sorbitol gradient. Female C57BL/6 mice (Harlan, UK) were infected i.p. with 3 × 10^4^ plaque forming units (PFU) of mCMV^73^. Whole blood samples were collected from individual mice on day 2 post infection, using uninfected C57BL/6 mice as controls.

For ***L. monocytogenes*** **infection**, *L. monocytogenes* was originally obtained from Drs. H. Rogers, K. Murphy, and E. Unanue, DNAX Research Institute, USA. Bacteria were grown in BHI broth (BD BBL) to mid-log phase as determined by OD_560_ and cryopreserved in 20% glycerol/PBS at −80 °C. Female C57BL/6 mice were infected by intravenous (i.v.) injection of 5 × 10^3^ CFU of *L. monocytogenes* derived from cryopreserved stocks diluted in 200 μl of PBS^8^. Control uninfected mice received PBS only. Whole blood samples were collected from individual mice on day 3 post infection, using uninfected C57BL/6J mice as controls.

### RNA isolation

Blood was collected in Tempus reagent (Life Technologies) at 1:2 ratio. Total RNA was extracted using the PerfectPure RNA Blood Kit (5 PRIME). Globin RNA was depleted from total RNA (1.5–2 µg) using the Mouse GLOBINclear kit (Thermo Fisher Scientific). Tissues were collected in TRI-Reagent (Sigma-Aldrich). Total RNA was extracted using the RiboPure™ Kit (Ambion). FACS sorted blood/lung cells were collected into TRI-Reagent LS (Sigma-Aldrich). Total RNA was extracted using the Purelink RNA microkit (Thermo Fisher). BAL cell pellets were obtained from pooled lavage fluid from each mouse, washed once in PBS and lysed in 350 μl RLT buffer. Lysates were passed through QIAshredder columns (QIAGEN). RNA was extracted using the RNeasy mini kit as per manufacturer’s instructions, including on-column DNase I digestion (both QIAGEN). All RNA was stored at −80 °C until use.

### Quantity and quality of RNA samples

Quantity was verified using NanoDrop™ 1000/8000 spectrophotometers (Thermo Fisher Scientific). Quality and integrity of the total and the globin-reduced RNA were assessed with the HT RNA Assay Reagent kit (Perkin Elmer) using a LabChip GX bioanalyser (Caliper Life Sciences/Perkin Elmer) and assigned an RNA Quality Score (RQS) or RNA 6000 Pico kit (Agilent) using a BioAnalyzer 2100 (Agilent) and assigned an RNA Integrity (RIN) score. RNA with an RQS/RIN >6 was used to prepare samples for microarray or RNA-seq.

Supplementary Data 20 provides details of each sample provided including QC data such as the RIN, RNA conc, 260/280 ratio, # reads sequenced, # reads aligned to genome, # reads aligned to genes by HTSeq, etc.

### Microarray

cRNA was prepared from 200 ng globin-reduced blood RNA or 200 ng tissue total RNA using the Illumina TotalPrep RNA Amplification Kit (Ambion). Quality was checked using an RNA 6000 Nano kit (Agilent) using a BioAnalyzer 2100 (Agilent). Biotinylated cRNA samples were randomized; 1.5 µg cRNA was then hybridized to Mouse WG-6 v2.0 bead chips (Illumina) according to the manufacturer’s protocols.

### RNA-seq

cDNA library preparation: for blood and tissues, total/globin-reduced RNA (200 ng) was used to prepare cDNA libraries using the TruSeq Stranded mRNA HT Library Preparation Kit (Illumina). For cDNA library preparation of FACS sorted cells, total RNA (30–500 pg) was used to prepare cDNA libraries using the NEBNext® Single Cell/Low Input RNA Library Prep Kit NEBNext® Multiplex Oligos for Illumina® #E6609 (New England BioLabs). Quality and integrity of the tagged libraries were initially assessed with the HT DNA HiSens Reagent kit (Perkin Elmer) using a LabChip GX bioanalyser (Caliper Life Sciences/Perkin Elmer). Tagged libraries were then sized and quantitated in duplicate (Agilent TapeStation system) using D1000 ScreenTape and reagents (Agilent). Libraries were normalized, pooled and then clustered using the HiSeq® 3000/4000 PE Cluster Kit (Illumina). The libraries were imaged and sequenced on an Illumina HiSeq 4000 sequencer using the HiSeq® 3000/4000 SBS kit (Illumina) at a minimum of 25 million paired-end reads (75 bp/100 bp) per sample.

### Histology

Lung, liver, and spleen tissues from *T. gondii* infected C57BL/6/J, *Ifnar*^*−/−*^, Ifngr^*−/−*^ and *Ifnar*^*−/−*^Ifngr1^*−/−*^ mice were fixed in 10% neutral-buffered formalin followed by 70% ethanol, processed and embedded in paraffin, sectioned (lung and liver, single lobe; spleen, longitudinal (or in fewer cases) transverse sections) at 4 µm and stained with hematoxylin and eosin (H&E). A single section from each tissue was viewed and scored as a consensus by two board-certified veterinary pathologists (E.W.H. and S.L.P.) blinded to the groups. A semi-quantitative scoring method was devised to assess the following histological features; inflammation (granulocytes and mononuclear cells), necrosis and presence of thrombosis with coagulative necrosis (for liver only): 0 = no lesion present, 1 = mild changes, and 2 = moderate or marked changes.

### Microscopy for neutrophil quantification

Lung sections from *T. gondii* infected C57BL/6J, *Ifnar*^*−/−*^, *Ifngr*^*−/−*^ and double *Ifnar*^*−/−*^ × *Ifngr*^*−/−*^ mice were de-waxed, re-hydrated, and treated with a standard antigen retrieval protocol (Target Retrieval Solution pH 9.0, Agilent Technologies at 97 °C for 45 min) before immunofluorescence staining. For neutrophil staining, sections were incubated with primary antibodies Goat anti-Human/Mouse Myeloperoxidase (AF3667, R&D), followed by Alexa Fluor 488-conjugated donkey anti‐goat (A11055, Life Technologies) and DAPI. Stained lung tissues were mounted with ProLong Gold Antifade Mountant (Life Technologies) and examined by confocal microscopy. Image analysis was performed using ImageJ. For neutrophil quantitation, 4–5 nonoverlapping fields per section were photographed at 40× magnification by Leica SP5 microscope and neutrophil numbers per field were counted based on myeloperoxidase (MPO) staining and neutrophil morphology (lobulated nuclei).

### Power calculation for modular derivation

The rationale for the a priori power calculation for number of mouse samples required for derivation of modules: Mead's resource equation^74^ was used for the a priori estimate of sample sizes for laboratory animals. An a priori statistical power analysis was not possible without information on the variability of transcriptomic experiments for all of the datasets, nor information on what magnitude of effect would be sufficiently significant. In addition, modular derivation is an exploratory approach that does not test any hypotheses.

Mead's resource equation: *E* = *N* − *B* − *T*

*N*: total number of mice in the study minus 1; *B*: blocking component, the number of environmental effects allowed for in the design minus 1; *T*: treatment component, the number of groups being used minus 1; *E*: degrees of freedom of the error component, and should be between 10 and 20.

For our study, we used two study groups per dataset (*T* = 1) and no differences in environment between groups (*B* = 0). Using those numbers with the above equation, and setting *E* to a value between 10 and 20, *N* is determined to be a value between 11 and 21. Therefore, we could have used between 12 and 22 animals for each dataset. A rounded number was chosen at the high end of the range, taking into consideration the large number of variables being measured. The solved equation: (10–20) = (11 – 21) − 0 − 1.

### RNA-seq data analysis

Raw paired-end RNA-seq data was subjected to quality control using FastQC (Babraham Bioinformatics) and MultiQC^75^. Trimmomatic^76^ v0.36 was used to remove the adapters and filter raw reads below 36 bases long, and leading and trailing bases below quality 25. The filtered reads were aligned to the *Mus musculus* genome Ensembl GRCm38 (release 86) using HISAT2^77^ v2.0.4 with default settings and RF rna-strandedness, including unpaired reads, resulting from Trimmomatic, using option -U. The mapped and aligned reads were quantified to obtain the gene-level counts using HtSeq^78^ v0.6.1 with default settings and reverse strandedness. Raw counts were processed using the bioconductor package DESeq2^79^ v1.12.4 in R v3.3.1, and normalized using the DESeq method to remove the library-specific artefacts. Variance stabilizing transformation was applied to obtain normalized log_2_ gene expression values. Further quality control was performed using principal component analysis, boxplots, histograms and density plots. Differentially expressed genes were calculated using the Wald test in DESeq2^79^. Genes with log_2_ fold change >1 or <−1 and false discovery rate (FDR) *p*-value < 0.05 corrected for multiple testing using the Benjamini–Hochberg (BH) method^80^ were considered significant. For module generation, and modular fold enrichment, only protein coding genes were considered Ensembl gene biotypes—protein coding, immunoglobulin genes IG-C, -D, -J, -LV and -V, and T cell receptor genes TR-C, -D, -J and -V.

### Microarray data analysis

Microarray data was processed in GeneSpring GX v14.8 (Agilent Technologies). Flags were used to filter out the probe sets that did not result in a “present” call in at least 10% of the samples, with the “present” lower cut-off of 0.99. Signal values were then set to a threshold level of 10, log_2_ transformed, and per-chip normalized using 75th percentile shift algorithm. Next, per-gene normalization was applied by dividing each messenger RNA transcript by the median intensity of all the samples. Next, transcripts were filtered to select the most variable probes: those that had a minimum of 1.5-fold expression change compared with the median intensity across all samples, in greater than 10% of all samples. For modular fold enrichment analysis, Illumina IDs were converted to Ensembl IDs using the annotation file available from Illumina, retaining IDs with one to one mapping.

### Cellular deconvolution

Deconvolution analysis for quantification of relative levels of distinct cell types on a per sample basis was carried out on normalized counts using CIBERSORT^39^. CIBERSORT estimates the relative subsets of RNA transcripts using linear support vector regression. Mouse cell signatures for 25 cell types were obtained using ImmuCC^40^ and grouped into 9 representative cell types based on the application of ImmuCC cellular deconvolution analysis to the sorted cell RNA-seq samples from the ImmGen ULI RNA-seq dataset (Supplementary Fig. 1).

### Module generation

Weighted gene co-expression network analysis was performed to identify lung and blood modules using the package WGCNA^42^ in R. Modules were constructed independently in lung and blood samples, across all control and disease samples from the 7 mouse models of infectious and inflammatory diseases, using log_2_ RNA-seq expression values. The lung modules were constructed using the 10,000 genes with the highest covariance across all lung samples, and the blood modules were constructed using 10,000 genes with the highest covariance across all blood samples. For subsequent analysis to generate modules, same parameters were used to construct the lung and blood modules in independent analyses. A signed weighted correlation matrix containing pairwise Pearson correlations between all the genes across all the samples was computed using a soft threshold of *β* = 22 to reach a scale-free topology. Using this adjacency matrix, the topological overlap measure (TOM) was calculated, which measures the network interconnectedness^81^ and is used as input to group highly correlated genes together using average linkage hierarchical clustering. The WGCNA dynamic hybrid tree-cut algorithm^82^ was used to detect the network modules of co-expressed genes with a minimum module size of 20, and deep split = 2. Lung modules were numbered L1–L38, and blood modules were numbered B1–B41, an additional “grey” module was identified in both lung modules (Supplementary Data 2, module titled NA), and blood modules (Supplementary Data 3, module titled NA) consisting of genes that were not co-expressed with any other genes. These grey modules were not considered in any further analysis. To create gene interaction networks, hub genes with high intramodular connectivity and a minimum correlation of 0.75 were calculated, with a cut-off of 50 hub genes, and exported into Cytoscape v3.4.0 for visualization.

### Modular annotation

Lung and blood modules were enriched for biological pathways and processed using IPA (QIAGEN Bioinformatics), Metacore (Thomson Reuters), and the GO database. Significantly enriched canonical pathways, and upstream regulators were obtained from IPA (top 5). GO analysis was performed for the biological processes ontology domain, using the bioconductor package clusterProfiler^83^ v3.0.5 in R. Over-representation analysis was performed using the BH method, with *p*valueCutoff = 0.01 and *q*valueCutoff = 0.05. Redundant GO terms were removed using the simplify function in the clusterProfiler package, using the Wang similarity measure and a similarity cut-off of 0.7, and the top 10 terms were considered. Modules were assigned names based on representative biological processes from pathways and processes from all three tools (Supplementary Data 4 and 5).

### Module preservation analysis

Modular preservation of lung modules in blood, and of blood modules in lung was performed using modulePreservation function in the WGCNA package in R, to assess whether the density (how tight interconnections among genes in a module are), overlap in module membership, and connectivity patterns of individual modules defined in a reference data set are preserved in a test data set^84^. The modulePreservation function performs a permutation test (*n* = 30 permutations) to generate a composite *Z*_summary_ preservation statistic, which summarizes the evidence that the network connections of the module are more significantly preserved than those of random set of genes of equal size. Modules with a *Z*_summary_ score > 10 are considered strongly preserved, a *Z*_summary_ score between 2 and 10 indicates weak to moderate preservation, and modules with *Z*_summary_ scores <2 are considered not preserved^84^. Chord diagrams to visualize module membership of the genes between the lung and the blood modules were constructed using the package circlize^85^ v0.4.3 in R, for the 6,999 genes in common between the 10,000 genes used to construct the lung modules, and 10,000 genes used to construct the blood modules.

### Module enrichment analysis

Fold enrichment for the WGCNA modules was calculated using the quantitative set analysis for gene expression (QuSAGE)^43^ using the bioconductor package qusage v2.4.0 in R, to identify the modules of genes over- or under-abundant in a dataset, compared to the respective control group using log_2_ expression values. The qusage function was used with default n.points parameter (2^12^), expect when the analysis was performed in groups with smaller sample sizes (*n* ≤ 5) (Test HDM allergy dataset, Toxoplasma WT and IFN KO dataset across all tissues; Supplementary Data 14), where the n.points parameter was set to 2^16^. Only modules with enrichment scores with FDR *p*-value < 0.05 were considered significant, and plotted using the ggcorrplot function in R.

### Single sample enrichment analysis

Enrichment of modules and subset of genes within modules on a single sample basis was carried out using gene set variation analysis (GSVA) using the bioconductor package gsva in R^86^. The enrichment scores obtained were similar to those from Gene Set Enrichment Analysis (GSEA), but based on absolute expression to quantify the degree to which a gene set is over-represented in a particular sample, rather than differential expression between two groups.

### Cell-type-specific signatures and enrichment

Raw RNA-seq counts for separated cells, representing 10 distinct cell type populations, were downloaded from the ImmGen ULI RNA-seq dataset from the Gene Expression Omnibus (GEO) database (GEO accession: GSE109125). Raw counts were processed, as described above, using the bioconductor package DESeq2^79^ v1.12.4 in R, and normalized using the DESeq method to remove the library-specific artefacts. Variance stabilizing transformation was applied to obtain normalized log_2_ gene expression values. Differentially expressed genes were obtained from the 5,000 genes with the highest variance across all samples by comparing each cell type against all other cell types, using the bioconductor package limma^87^ v3.28.21 in R. Only upregulated genes with log_2_ foldchange >1 and FDR *p*-value < 0.05 were considered cell-type specific. Cell-type enrichment analysis to identify over-represented cell types in lung and blood modules was performed using a hypergeometric test, using the phyper function in R. *p*-Values were corrected for multiple testing using the p.adjust function in R, using the BH method, to obtain FDR corrected *p*-values.

### In vitro-derived T helper cell signatures generation

Raw single-end fastq files were downloaded from GEO database (GEO accession: GSE106464), for T_H_1+IL-27, T_H_2 and T_H_17 cells at 6 h. Fastq files were processed as described above, but using unstranded mapping options, to obtain raw counts. Raw counts were processed, as described above, using the bioconductor package DESeq2^79^ v1.12.4 in R, and normalized using the DESeq method to remove the library-specific artefacts. Variance stabilizing transformation was applied to obtain normalized log_2_ gene expression values. Differentially expressed genes were obtained by comparing each cell type against all other cell types, using the bioconductor package limma^87^ v3.28.21 in R. Only upregulated genes with log_2_ foldchange >1 and FDR *p*-value < 0.05 were considered T helper cell-type specific.

### Mapping mouse samples to the *Toxoplasma gondii* genome

The filtered RNA-seq fastq files from the mouse samples from Toxoplasma WT and IFN receptor KO dataset across all tissues (obtained as described above), were aligned to the *T. gondii* genome ToxoDB 7.1 Ensembl GRCm38 (release 35) using HISAT2^77^ v2.0.4 with default settings and RF rna-strandedness, including unpaired reads resulting from Trimmomatic using option -U. The mapped and aligned reads were quantified to obtain the gene-level counts using HtSeq^78^ v0.6.1 with default settings and reverse strandedness. Raw library sizes were calculated for each sample as the sum of read counts for all genes in that sample. Normalization factors, calculated from the original normalization analysis of the *Toxoplasma* WT and IFN receptor KO dataset across all tissues (using the *M. musculus* genome), using the estimateSizeFactors function from the DESeq2 package in R, were multiplied to the raw library sizes to obtain normalized library sizes, to quantify the presence of the *T. gondii* pathogen present in the mouse lung, blood, liver, and spleen samples in wildtype and IFN receptor KO mice.

### Interferome database analysis

IFN response genes (type I, type II, and type I and II) listed in the Interferome database^44^ (release v2.01; www.interferome.org accessed December 2019) were identified with the blood and lung modules from Fig. 2 (Supplementary Datas 2 and 3).

### Method for use of online WebApp

An online webapp https://ogarra.shinyapps.io/MouseModules/ accompanies the manuscript to visualize the findings of the study. The app is subdivided into 5 distinct pages that can be accessed through the tabs displayed on the top of the page, with a customized sidebar for user input on each page.

Tab 1: “**Gene** **expression**” allows the user to input a gene of interest (either a gene symbol or a mouse Ensembl ID) to visualize its expression (either as counts or log2 expression values) across 5 different datasets consisting of mouse models of infection and inflammation, as described in the manuscript. Each dot represents an individual sample, grouped together as controls and disease samples across the different mouse models.

Tab 2: “**Gene** **lookup in modules**” allows the user to input a gene of interest (either a gene symbol or a mouse Ensembl ID) and find out which lung and blood module it belongs to. Thirty-eight lung and forty-one blood modules were derived as part of the study from lung and blood samples obtained from six mouse models of infection and inflammation.

Tab 3: **“Lung modules”** allows the user to visualize the expression of each lung module (L1–L38) across lung samples obtained from six mouse models of infection and inflammation. The enrichment score between −1 and 1 represents the combined overall expression of all genes within the module for each sample. A table below the plot displays all genes present within that module.

Tab 4: **“Blood modules”** allows the user to visualize the expression of each blood module (L1–L41) across blood samples obtained from either the six mouse models used for module derivation, or the four distinct mouse models used for validation. The enrichment score between −1 and 1 represents the combined overall expression of all genes within the module for each sample. A table below the plot displays all genes present within that module.

Tab 5: **“Download data”** allows the user to download the genes present in all lung and blood modules, and the biological annotation of these modules.

For all plots in the app, the user can manually set the width and height of the plot, and download them as png files. Additionally, the user can interact with the plot by hovering over the data points to obtain detailed information for each sample point, as well as summary statistics for each group.

## Supplementary Information

Supplementary Information

Description of Additional Supplementary Files

Supplementary Data 1

Supplementary Data 2

Supplementary Data 3

Supplementary Data 4

Supplementary Data 5

Supplementary Data 6

Supplementary Data 7

Supplementary Data 8

Supplementary Data 9

Supplementary Data 10

Supplementary Data 11

Supplementary Data 12

Supplementary Data 13

Supplementary Data 14

Supplementary Data 15

Supplementary Data 16

Supplementary Data 17

Supplementary Data 18

Supplementary Data 19

Supplementary Data 20

Reporting summary

## Data Availability

The materials, data, code, and any associated protocols that support the findings of this study are available from the corresponding author upon request. The Microarray and RNA-seq datasets have been deposited in the NCBI Gene Expression Omnibus (GEO) database with the primary accession number GSE119856. Publically available datasets used in this study include GSE109125 (sorted cells from Immunological Genome Project), GSE106464 (in vitro differentiated T helper cells), and GSE61106 (*Burkholderia pseudomallei* (acute) microarray).
