# Supplementary material for: Transcriptional profiling unveils type I and II interferon networks in blood and tissues across diseases
Source: Nat Commun. 2019 Jun 28;10:2887. doi: 10.1038/s41467-019-10601-6 (PMC6599044; doi:10.1038/s41467-019-10601-6)
Supplement: Supplementary file 1 — Supplementary Information [file 41467_2019_10601_MOESM1_ESM.pdf]

Supplementary Information

Transcriptional profiling unveils type I and II interferon networks in blood and tissues across diseases

Singhania et al.

Mouse models of a distinct set of infectious diseases

a

Disease	Nomenclature	Type	Investigator
Plasmodium chabaudi (P. chabaudi)	Malaria	Parasite	Jean Langhorne (Crick)
Murine Cytomegalovirus (MCMV)	MCMV	Virus	Ian Humphreys (Cardiff)
Listeria monocytogenes (L. monocytogenes)	Listeria	Bacteria	Anne O'Garra (Crick)
Burkholderia pseudomallei chronic (B. pseudomallei)	Bps chronic	Bacteria	Greg Bancroft (LSHTM)

b

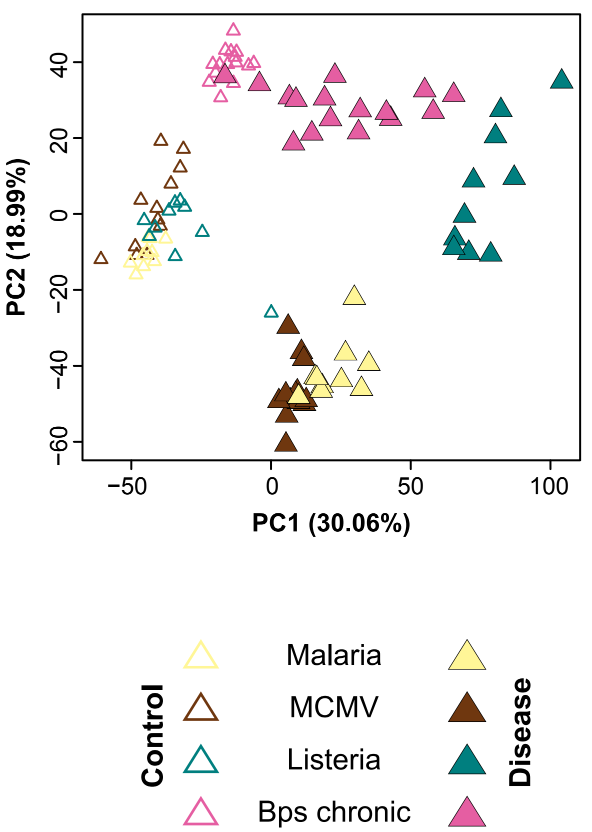

c

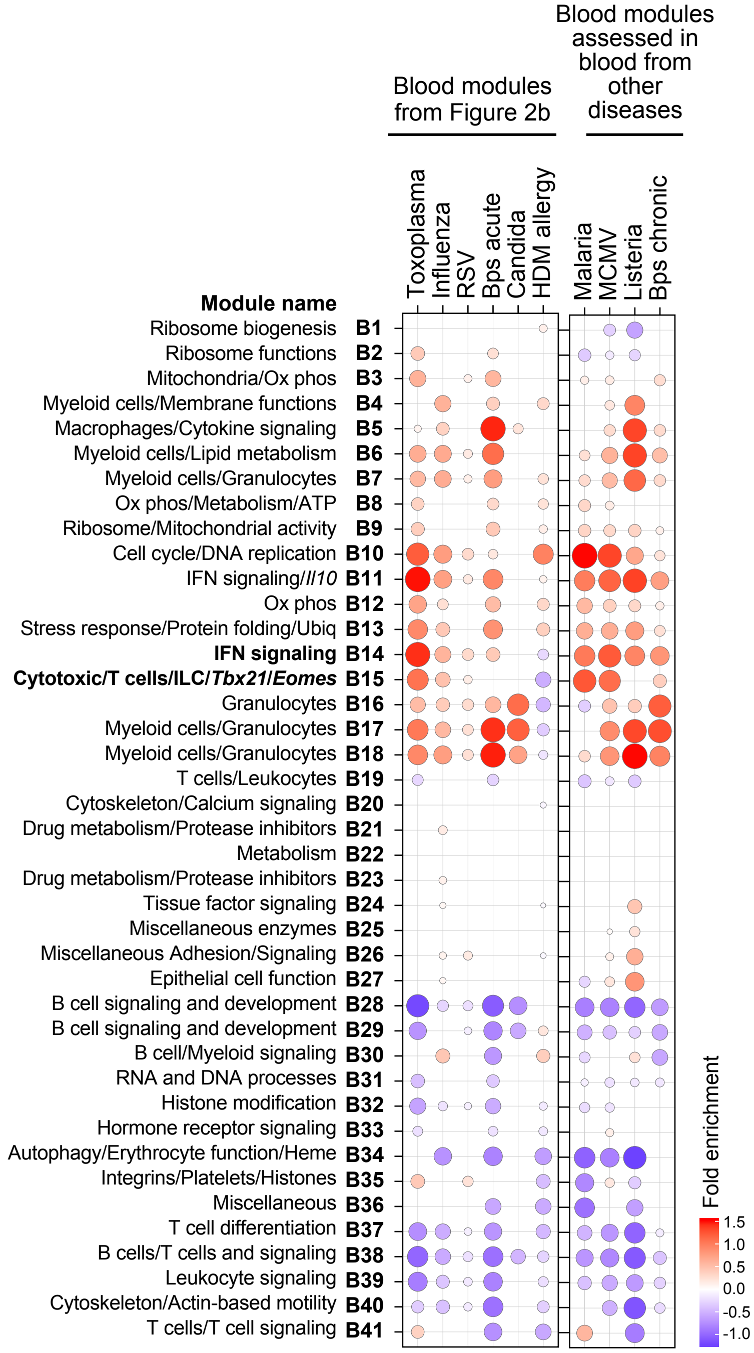

Supplementary Figure 1

Supplementary Figure 1. Blood modular transcriptional signature assessed in a set of distinct infectious diseases. a, RNA-seq analysis was performed on blood samples (Supplementary table 1) obtained from experimental mouse models of 4 infectious diseases, distinct from the original set of 6 diseases that were utilized in module generation. **b,** Principal component analysis in blood samples, depicting the variation in the global gene expression profiles across diseases. Principal components 1 (PC1) and 2 (PC2), which describe the greatest variation in gene expression, are shown. Empty and filled symbols represent control and disease samples, respectively, and color represents mouse models. **c,** Fold enrichment for the blood modules assessed in blood samples in the original set of 6 diseases utilized in module generation (fold enrichment plot from Figure 2b, plotted on a different scale), and assessed in the set of 4 distinct diseases. Fold enrichment scores were derived using QuSAGE, with red and blue circles indicating the cumulative over- or under-abundance of all genes within the module, for each disease compared to the respective controls. Color intensity of the dots represents the degree of perturbation, indicated by the color scale. Size of the dots represents the relative degree of perturbation, with the largest dot representing the highest degree of perturbation within the plot. Within each disease, only modules with FDR p-value < 0.05 were considered significant, and depicted here. Ox phos, oxidative phosphorylation; Ubiq, ubiquitination.

a Cellular deconvolution of the ImmGen ULI RNA-seq dataset

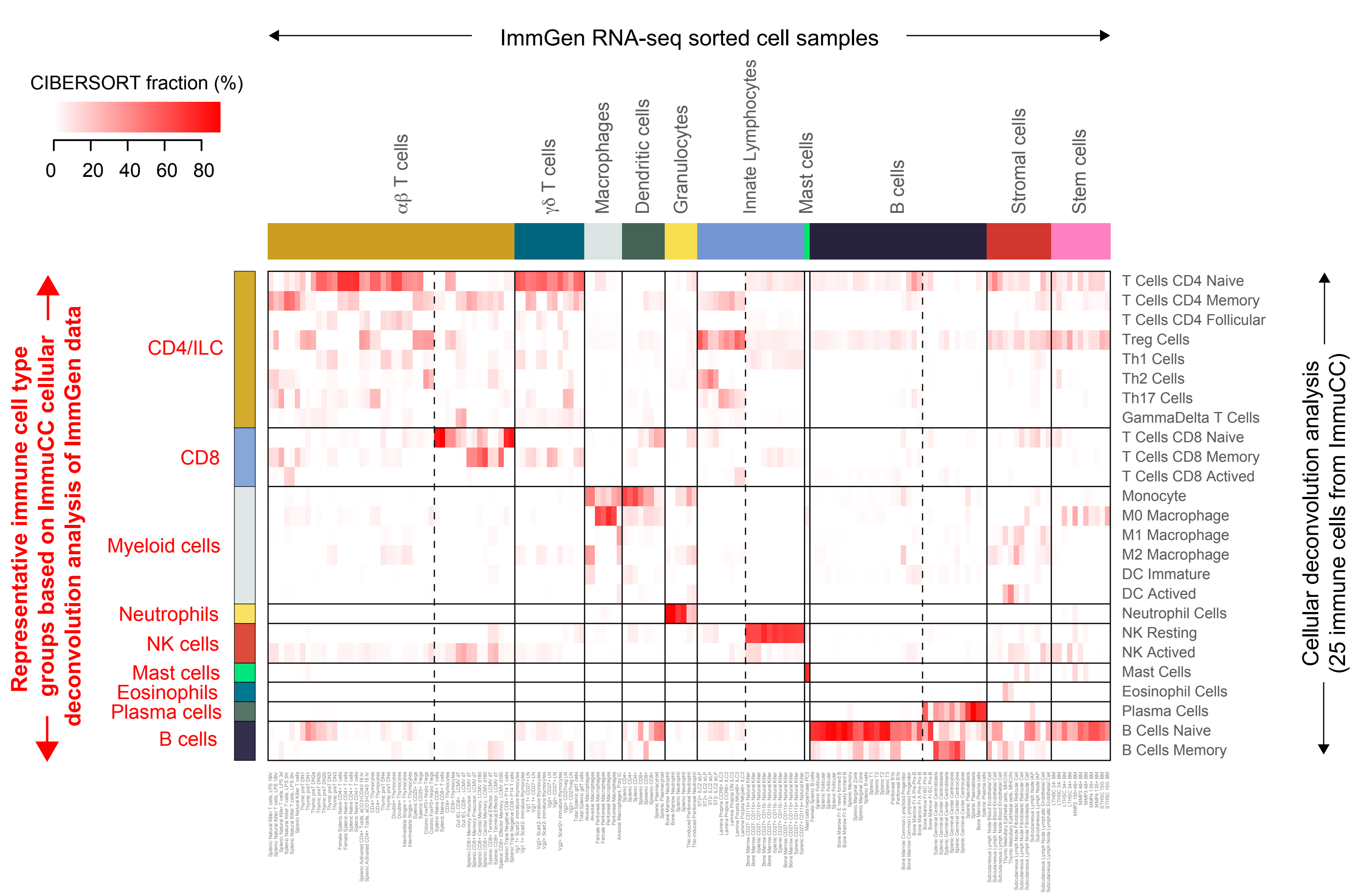

b Cellular deconvolution

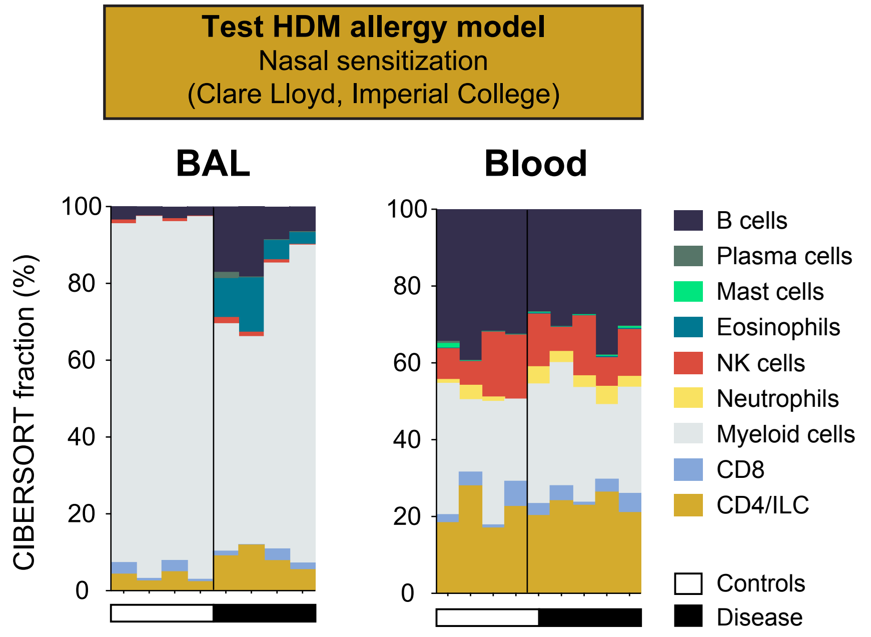

Supplementary Figure 2

Supplementary Figure 2. Cellular deconvolution anlaysis. a, Heatmap depicting in silico immune cell composition of RNA-seq samples of purified cell types from the ImmGen ULI RNA-seq dataset, derived using the CIBERSORT algorithm based on the 25 immune cellular signatures obtained from ImmuCC. Color represents percent CIBERSORT fraction, with darker the color representing higher the enrichment of a particular immune cell type in the RNA-seq samples of purified cell types from the ImmGen ULI RNA-seq dataset. Based on this enrichment, the 25 immune cell types from ImmuCC were grouped in to 9 representative cell types, as indicated on the right side in red. ILC, innate lymphoid cells; NK cells, natural killer cells. **b,** Stacked bar plots depicting in silico immune cell composition of bronchoalveolar lavage (BAL) and blood RNA-seq samples from a test HDM allergy model, derived using the CIBERSORT algorithm based on cellular signatures obtained from ImmuCC. Each bar represents percent fractions for 9 representative cell types for an individual mouse sample, with colors representing the different cell types. White and black bars at the bottom of each plot represent control and disease samples, respectively. ILC, innate lymphoid cells; NK cells, natural killer cells.

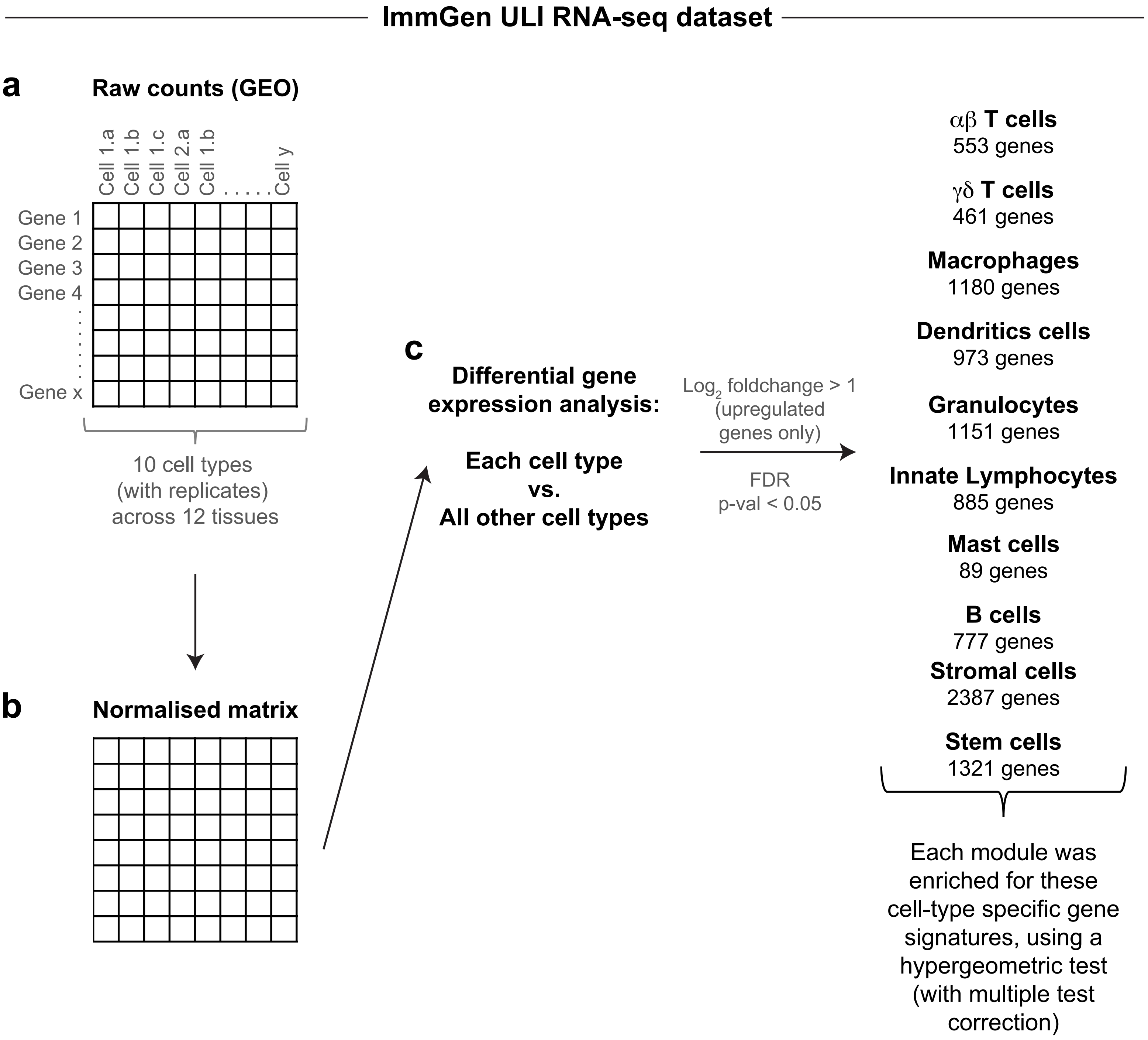

Supplementary Figure 3

Supplementary Figure 3. Cell-type specific signatures. a, RNA-seq raw counts for purified cells, representing 10 distinct cell type populations, from the ImmGen ULI RNA-seq dataset were downloaded from the Gene Expression Omnibus (GEO) database (GEO accession: GSE109125). **b,** Raw count data was normalised as described in the methods. **c,** Cell-type specific signatures were obtained by comparing each cell type against all other cell types. Only upregulated genes with log_2 foldchange > 1 and FDR p-value < 0.05 were considered cell-type specific.

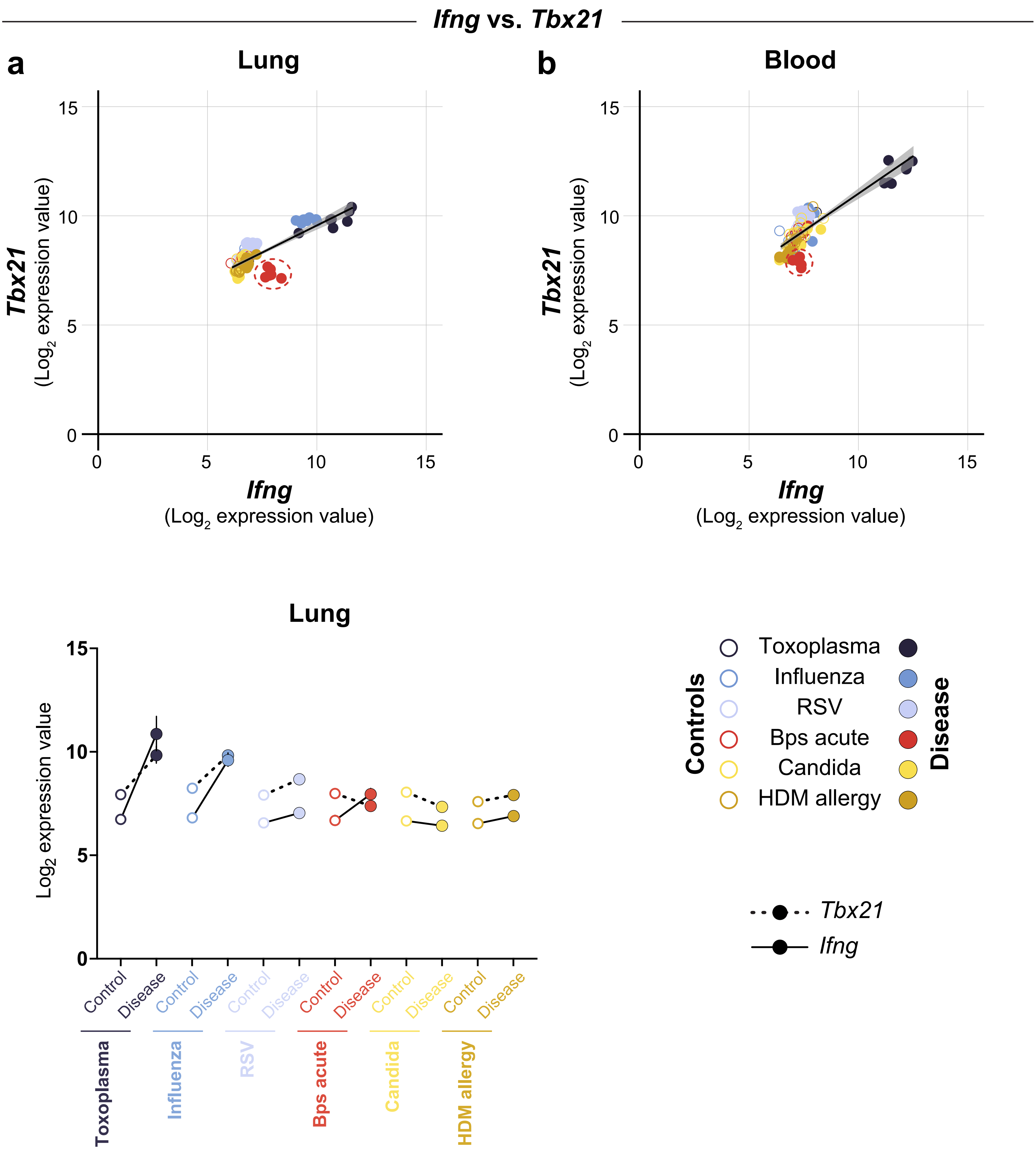

Supplementary Figure 4

Supplementary Figure 4. Co-expression of *Ifng* and *Tbx21* across infectious and inflammatory diseases. a,b, Scatter plot showing the log_2 expression values of *Ifng* (x-axis) and *Tbx21* (y-axis), in lung (**a**) and blood (**b**) samples from control and infected/challenged mice across the set of 6 diseases. Linear regression lines are shown, with shaded areas representing 95% confidence interval. The disease samples for *B. pseudomallei* acute infection are encircled. Log_2 co-expression profiles for *Ifng* (solid line) and *Tbx21* (dotted line) are also shown for lung samples (**a**). Empty and filled symbols represent control and disease samples, respectively, and color represents mouse models.

Lung RNA-seq modules assessed in Microarray data

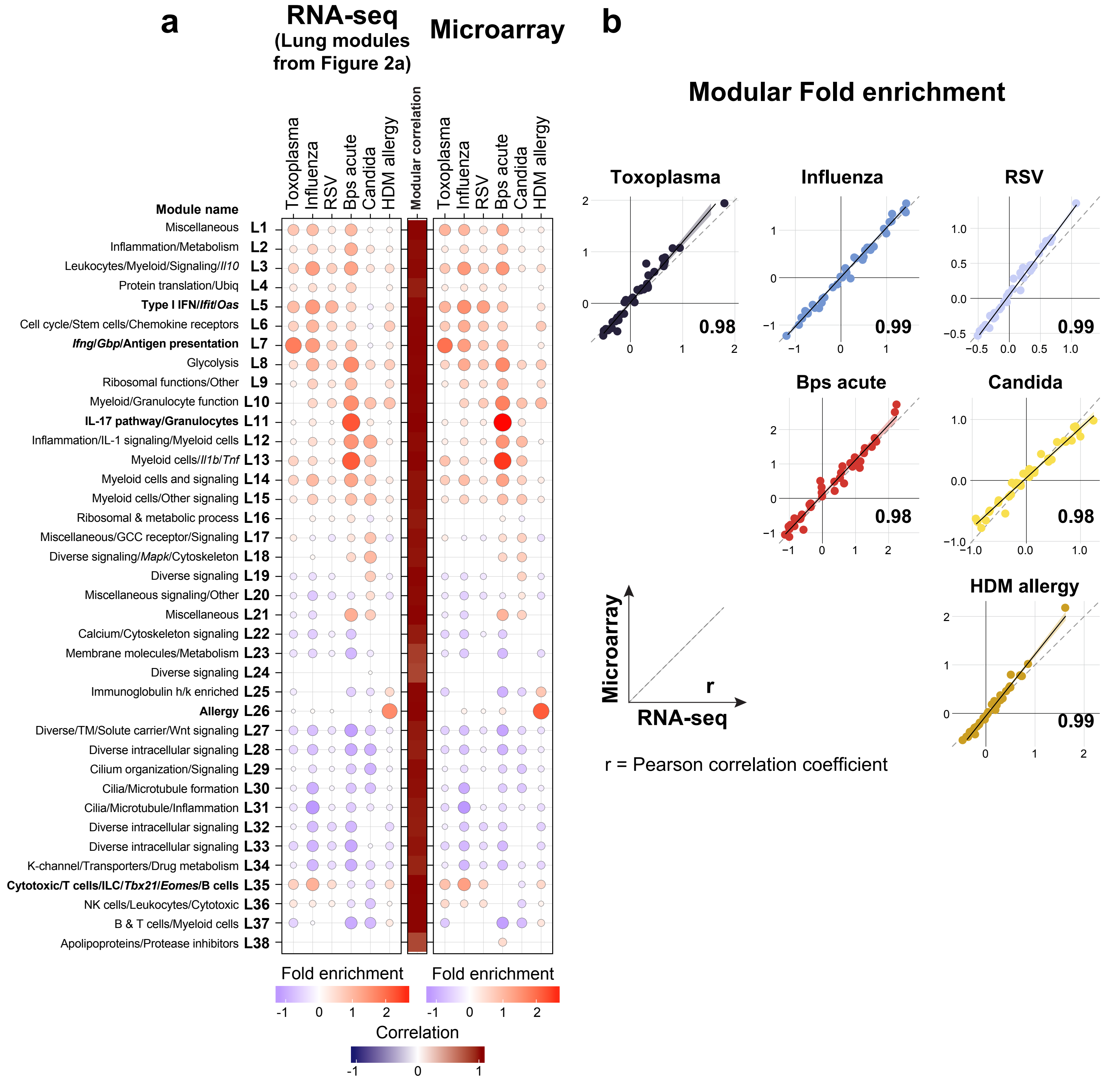

Supplementary Figure 5

Supplementary Figure 5. Enrichment of lung modular transcriptional signature in RNA-seq compared to microarray. a, Fold enrichment for lung modules in lung samples in the original set of 6 diseases utilized in module generation from data generated using RNA-seq (fold enrichment plot from Figure 2a), and assessed in the same set of 6 diseases from data generated using microarrays. Red and blue circles indicate the cumulative over- or under-abundance of all genes within the module, for each disease compared to the respective controls within each technology. Color intensity of the dots represents the degree of perturbation, indicated by the color scale. Size of the dots represents the relative degree of perturbation, with the largest dot representing the highest degree of perturbation within the plot. Within each disease, only modules with FDR p-value < 0.05 were considered significant and depicted here. Correlation between fold enrichment scores across the 6 diseases for each module between RNA-seq and microarray is shown, with dark red and blue squares representing positively and negatively correlated enrichment scores, respectively GCC, glucocorticoid; K-channel, potassium channel; TM, transmembrane; Ubiq, ubiquitination. **b**, Correlation of fold enrichment scores within each disease between RNA-seq (x-axis) compared to microarray (y-axis). Each dot represents fold enrichment score for an individual module. Dashed grey line within each plot at the 45-degree slope represents identical fold enrichment scores between RNA-seq and microarray, with fold enrichment scores above or below the line showing higher abundance in microarray or RNA-seq data, respectively. Linear regression lines with 95% confidence interval, and Pearson correlation coefficients (r) are shown for each plot.

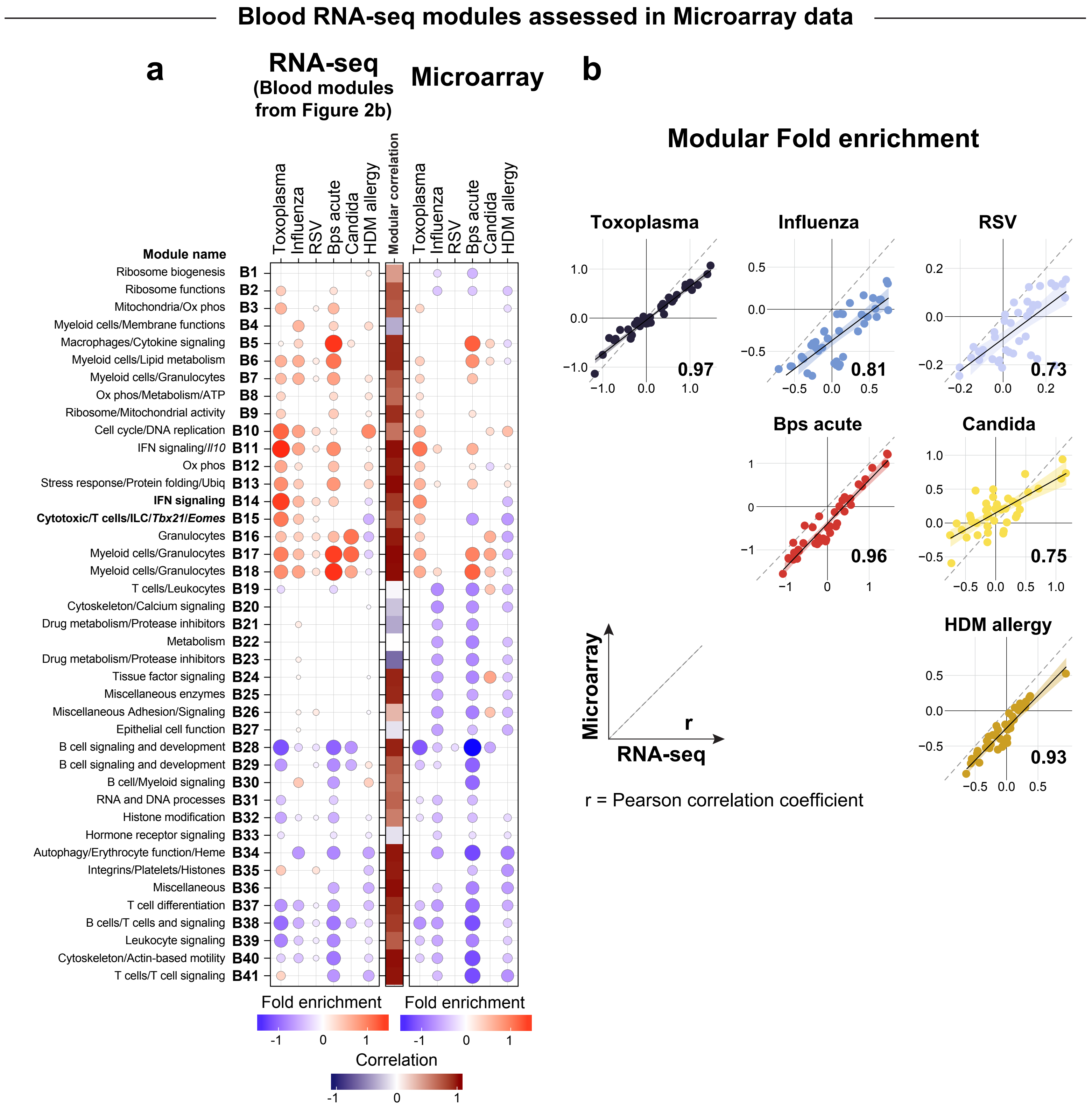

Supplementary Figure 6

Supplementary Figure 6. Enrichment of blood modular transcriptional signature in RNA-seq compared to microarray. a, Fold enrichment for blood modules in blood samples in the original set of 6 diseases utilized in module generation from data generated using RNA-seq (fold enrichment plot from Figure 2b), and assessed in the same set of 6 diseases from data generated using microarrays. Fold enrichment scores were derived using QuSAGE, with red and blue circles indicating the cumulative over- or under-abundance of all genes within the module, for each disease compared to the respective controls within each technology. Color intensity of the dots represents the degree of perturbation, indicated by the color scale. Size of the dots represents the relative degree of perturbation, with the largest dot representing the highest degree of perturbation within the plot. Within each disease, only modules with FDR p-value < 0.05 were considered significant and depicted here. Correlation between fold enrichment scores across the 7 diseases for each module between RNA-seq and microarray is shown, with red and blue squares representing positively and negatively correlated enrichment scores, respectively. Ox phos, oxidative phosphorylation; Ubiq, ubiquitination. **b**, Correlation of fold enrichment scores within each disease between RNA-seq (x-axis) compared to microarray (y-axis). Each dot represents fold enrichment score for an individual module. Dashed grey line within each plot at the 45-degree slope represents identical fold enrichment scores between RNA-seq and microarray, with fold enrichment scores above or below the line showing higher abundance in microarray or RNA-seq data, respectively. Linear regression lines with 95% confidence interval, and Pearson correlation coefficients (r) are shown for each plot.

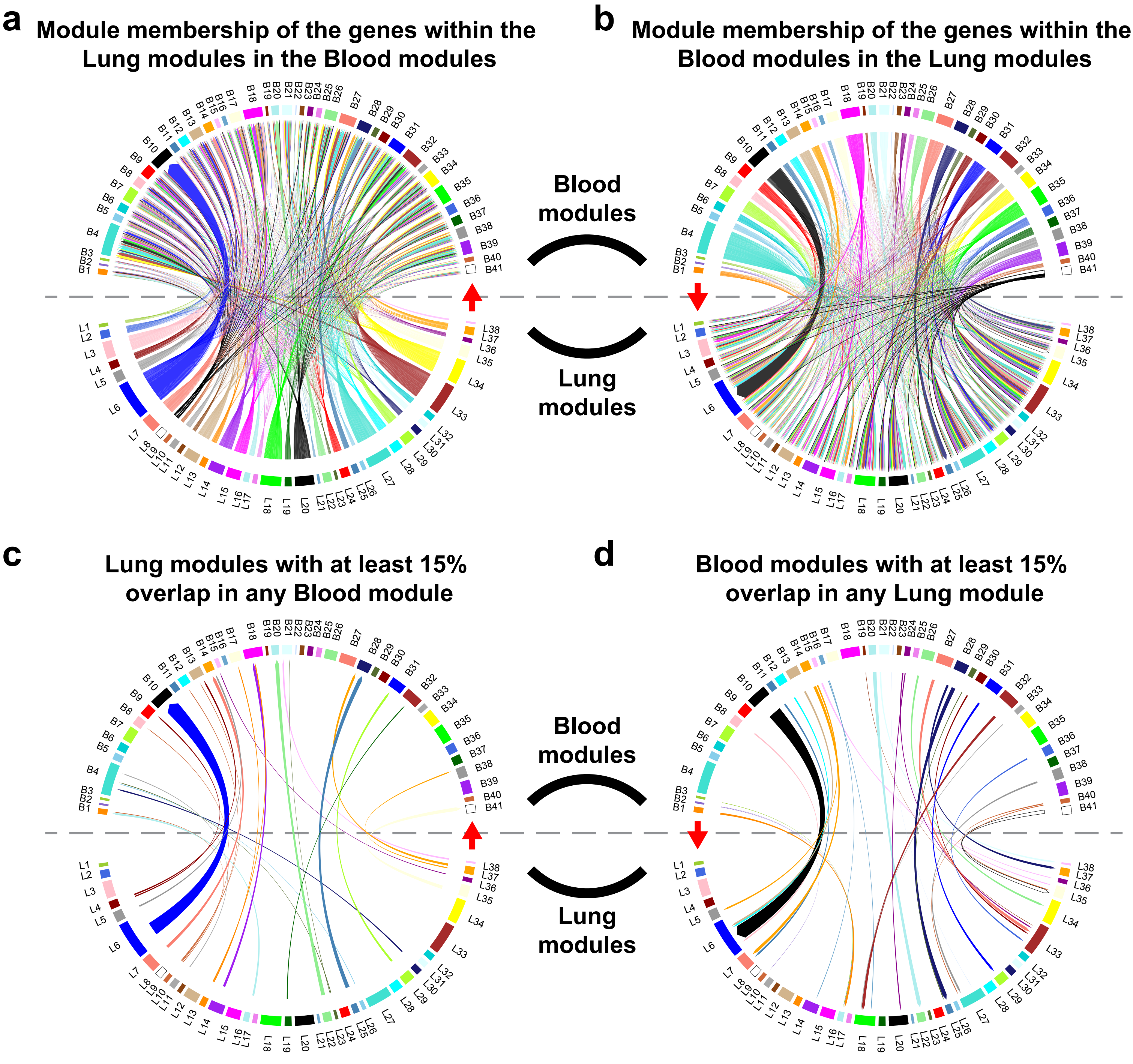

Lung modules

L1 Miscellaneous
L2 Inflammation/Metabolism
L3 Leukocytes/Myeloid/Signaling/*Il10*
L4 Protein translation/Ubiq
L5 Type I IFN/*Ifit*/*Oas*
L6 Cell cycle/Stem cells/Chemokine receptors
L7 *Ifng*/*Gbp*/Antigen presentation
L8 Glycolysis
L9 Ribosomal functions/Other
L10 Myeloid/Granulocyte function
L11 IL-17 pathway/Granulocytes
L12 Inflammation/IL-1 signaling/Myeloid cells
L13 Myeloid cells/*Il1b*/*Tnf*
L14 Myeloid cells and signaling
L15 Myeloid cells/Other signaling
L16 Ribosomal & metabolic process
L17 Miscellaneous/GCC receptor/Signaling
L18 Diverse signaling/*Mapk*/Cytoskeleton
L19 Diverse signaling
L20 Miscellaneous signaling/Other
L21 Miscellaneous
L22 Calcium/Cytoskeleton signaling
L23 Membrane molecules/Metabolism
L24 Diverse signaling
L25 Immunoglobulin h/k enriched
L26 Allergy
L27 Diverse/TM/Solute carrier/Wnt signaling
L28 Diverse intracellular signaling
L29 Cilium organization/Signaling
L30 Cilia/Microtubule formation
L31 Cilia/Microtubule/Inflammation
L32 Diverse intracellular signaling
L33 Diverse intracellular signaling
L34 K-channel/Transporters/Drug metabolism
L35 Cytotoxic/T cells/ILC/*Tbx21*/*Eomes*/B cells
L36 NK cells/Leukocytes/Cytotoxic
L37 B & T cells/Myeloid cells
L38 Apolipoproteins/Protease inhibitors

Blood modules

B1 Ribosome biogenesis
B2 Ribosome functions
B3 Mitochondria/Ox phos
B4 Myeloid cells/Membrane functions
B5 Macrophages/Cytokine signaling
B6 Myeloid cells/Lipid metabolism
B7 Ox phos/Metabolism/ATP
B8 Myeloid cells/Granulocytes
B9 Ribosome/Mitochondrial activity
B10 Cell cycle/DNA replication
B11 IFN signaling/*Il10*
B12 Ox phos
B13 Stress response/Protein folding/Ubiq
B14 IFN signaling
B15 Cytotoxic/T cells/ILC/*Tbx21*/*Eomes*
B16 Granulocytes
B17 Myeloid cells/Granulocytes
B18 Myeloid cells/Granulocytes
B19 T cells/Leukocytes
B20 Cytoskeleton/Calcium signaling
B21 Drug metabolism/Protease inhibitors
B22 Metabolism
B23 Drug metabolism/Protease inhibitors
B24 Tissue factor signaling
B25 Miscellaneous enzymes
B26 Miscellaneous Adhesion/Signaling
B27 Epithelial cell function
B28 B cell signaling and development
B29 B cell signaling and development
B30 B cell/Myeloid signaling
B31 RNA and DNA processes
B32 Histone modification
B33 Hormone receptor signaling
B34 Autophagy/Erythrocyte function/Heme
B35 Integrins/Platelets/Histones
B36 Miscellaneous
B37 T cell differentiation
B38 B cells/T cells and signaling
B39 Leukocyte signaling
B40 Cytoskeleton/Actin-based motility
B41 T cells/T cell signaling

Supplementary Figure 7

Supplementary Figure 7. Modular gene preservation between lung and blood. a,b, Module membership of the genes between the lung and the blood modules for the 6,999 genes in common between the 10,000 genes with the highest variance across all lung samples that were used to construct the lung modules, and 10,000 genes with the highest variance across all blood samples that were used to construct the blood modules. **c,d,** Only genes that show at least 15% of the total genes within a lung or blood module preserved in a corresponding blood or lung module, respectively. In the outer circular track, lung and blood modules are displayed on the bottom and top, respectively. Color of the line represents the color of the original module, i.e. if the gene was originally present in the lung module, but tested in the blood modules (**a,c**), or if the gene was originally present in the blood module, but tested in the lung modules (**b,d**). Module numbers are indicated and arbitrary colors are assigned to the modules for visual distinction. The size of the modular bar in the outer circular track represents the size of the module, i.e. the number of genes within that module. Each line in the inner circular area represents an individual gene, with arrows for each line representing where the gene in the lung module maps to the blood modules (**a,c**), and where the gene in the blood module maps to the lung modules (**b,d**). Thickness of the line represents the number of genes preserved. GCC, glucocorticoid; K-channel, potassium channel; Ox phos, oxidative phosphorylation; TM, transmembrane; Ubiq, ubiquitination.

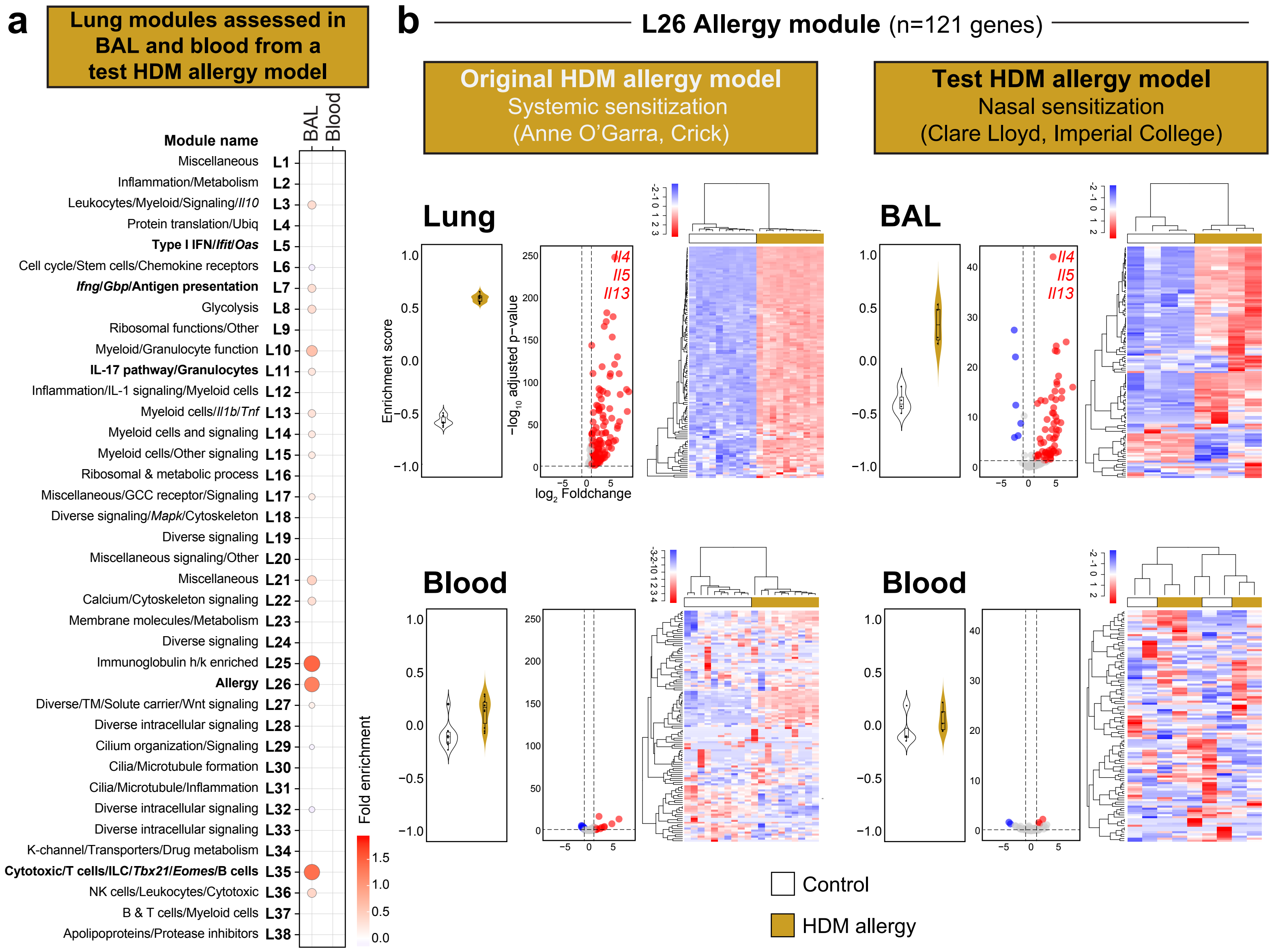

Supplementary Figure 8

Supplementary Figure 8. Lung modular transcriptional signature in HDM allergy. a, Fold enrichment for lung modules assessed in bronchoalveolar lavage (BAL) and blood samples obtained from a distinct model of HDM allergy. Red and blue circles indicate the cumulative over- or under-abundance of all genes within the module, for each tissue compared to the respective controls. Color intensity of the dots represents the degree of perturbation, indicated by the color scale. Size of the dots represents the relative degree of perturbation, with the largest dot representing the highest degree of perturbation within the plot. Within each sample group, only modules with FDR p-value < 0.05 were considered significant and depicted here. GCC, glucocorticoid; K-channel, potassium channel; TM, transmembrane; Ubiq, ubiquitination. **b,** Transcriptional profile of the lung Allergy (L26) module in the original and the test HDM allergy models. For each tissue in each HDM allergy model, violin plots, volcano plots and heatmaps are shown. Violin plots depict the frequency distribution of enrichment scores calculated on a single-sample basis using GSVA for the 121 genes in the L26 module. Each dot represents an individual sample, and box plots inside the violin plots show the median with interquartile range, along with the 95% confidence interval. Volcano plots depict the differentially expressed genes for disease compared to controls for the 121 genes in the L26 module. Significantly differentially expressed genes (log_2 fold change >1 or <−1, and FDR p-value < 0.05) are represented as red (upregulated) or blue (downregulated) dots. Differential gene expression for *Il4*, *Il5* and *Il13* is indicated in red within the volcano plot, depicting upregulation, in volcano plots where these genes were significantly differentially expressed in HDM allergy compared to controls. Heatmaps show log_2 gene expression values for the 121 genes in the L26 module. Gene expression values were averaged and scaled across the row to indicate the number of standard deviations above (red) or below (blue) the mean, denoted as row Z-score. Dendrograms show unsupervised hierarchical clustering of genes and samples, with distances calculated using Pearson correlation and clustered using the complete linkage.

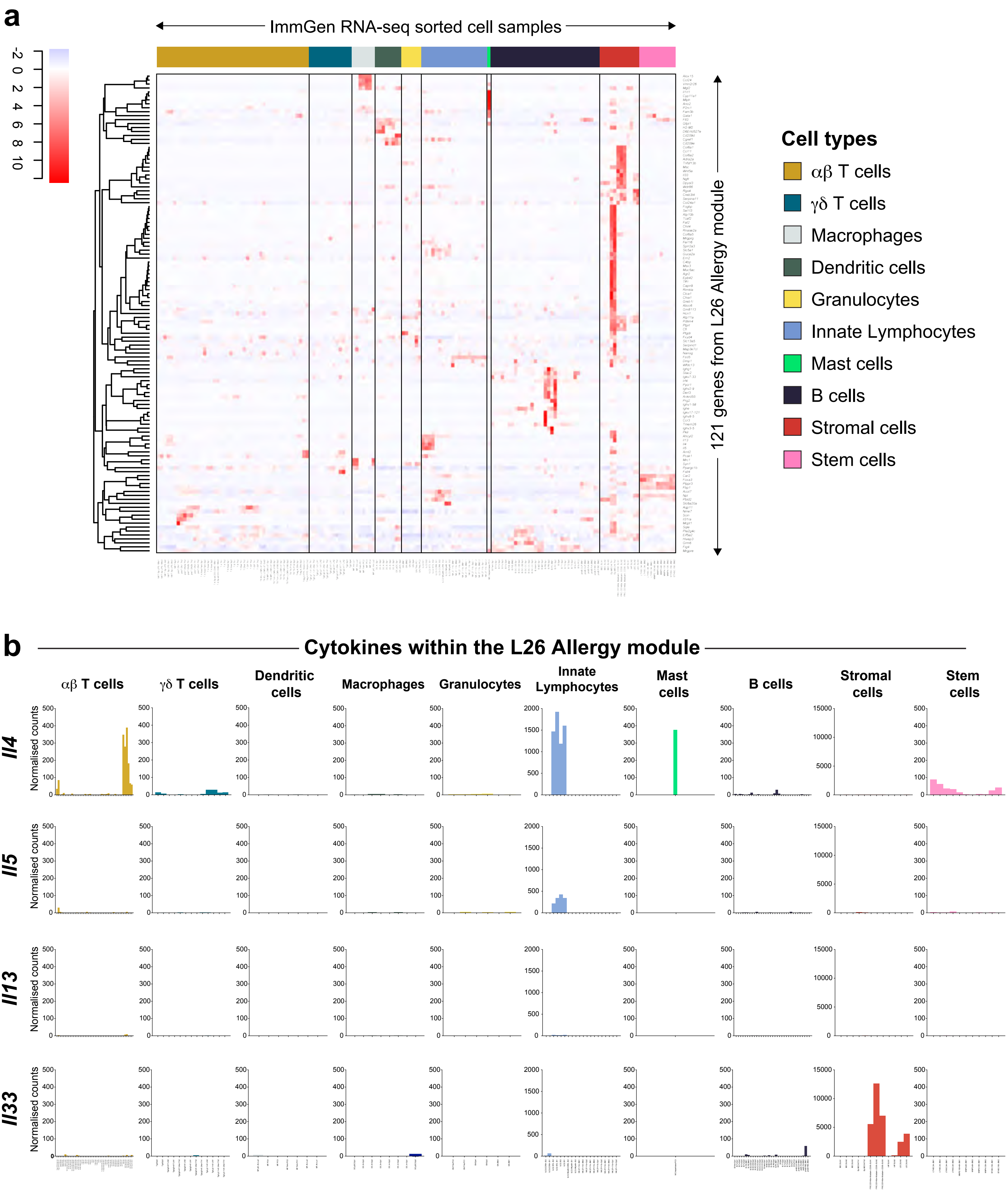

Supplementary Figure 9

Supplementary Figure 9. Predicted cell type specific source for the genes within the lung Allergy (L26) module. a, Heatmap depicting the normalised counts for the 121 genes in the L26 module across separated cells, representing 10 distinct cell type populations, obtained from the ImmGen ULI RNA-seq dataset from the Gene Expression Omnibus (GEO) database (GEO accession: GSE109125), with colors in the top bar representing the different cell types. Normalised counts were averaged and scaled across the row to indicate the number of standard deviations above (red) or below (blue) the mean, denoted as row Z-score. Dendrogram shows unsupervised hierarchical clustering of genes, with distances calculated using Pearson correlation and clustered using the complete linkage. **b,** Normalised counts of the genes encoding the cytokines IL-4, IL-5, IL-13 and IL-33, present within the L26 module. Each bar represents the normalised counts for an individual purified cell type within the cell population.

Toxoplasma gondii

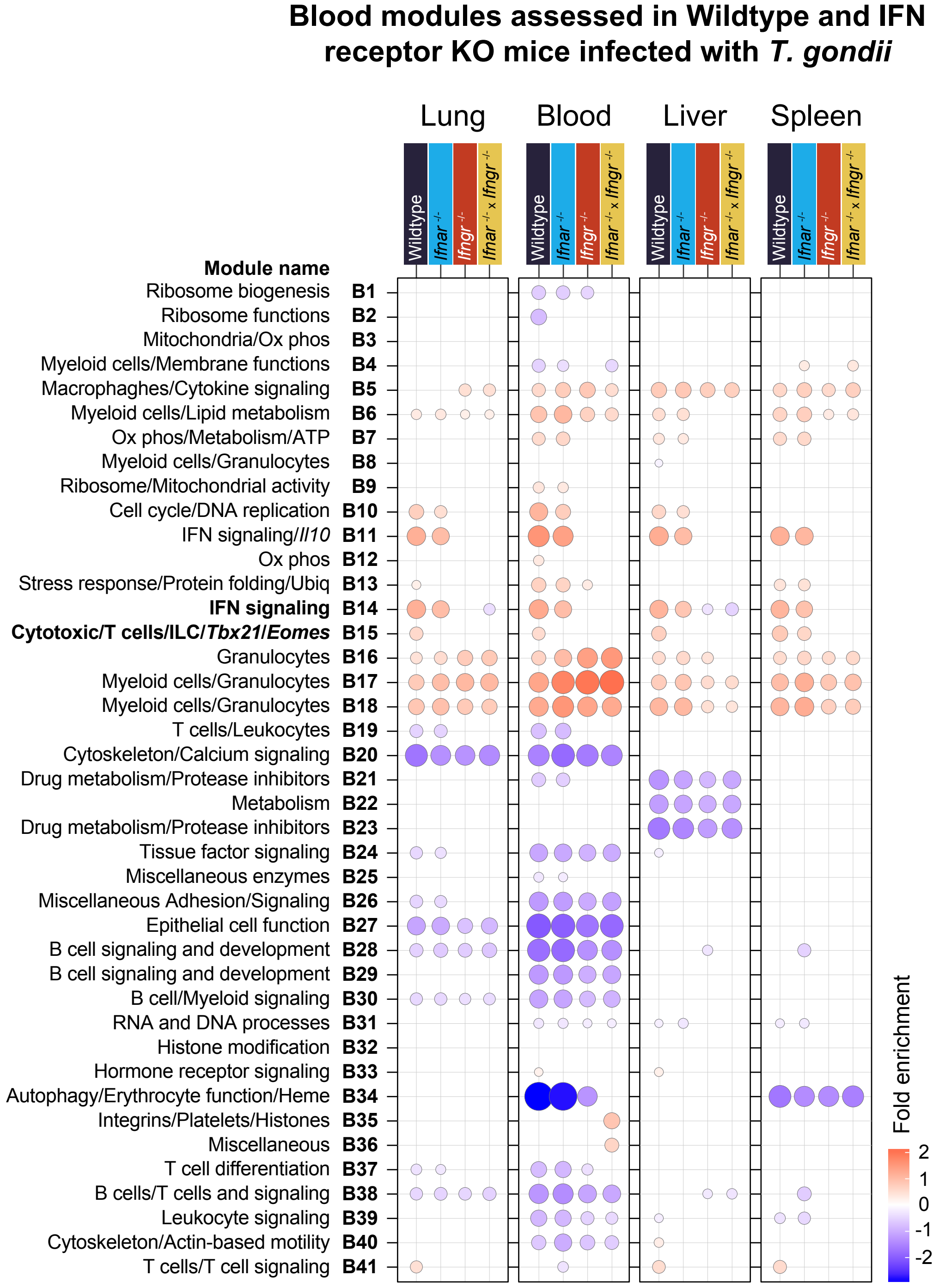

Supplementary Figure 10

Supplementary Figure 10. Changes in the blood modular transcriptional profiles across tissues following *T. gondii* infection, in the absence of Type I and/or II IFN signaling. Modular transcriptional profiles of lung, blood, liver and spleen, from *T. gondii* infected mice, assessed using the blood modules. Red and blue circles indicate the cumulative over- or under-abundance of all genes within the module, for each Wild type and IFN receptor knockout (KO) disease group compared to the respective Wild type controls in each tissue. Color intensity of the dots represents the degree of perturbation, indicated by the color scale. Size of the dots represents the relative degree of perturbation, with the largest dot representing the highest degree of perturbation within the plot. Within each group, only modules with FDR p-value < 0.05 compared to respective controls were considered significant and depicted here. Ox phos, oxidative phosphorylation; Ubiq, ubiquitination.

a IFN receptor KO controls compared to Wildtype controls in Lung

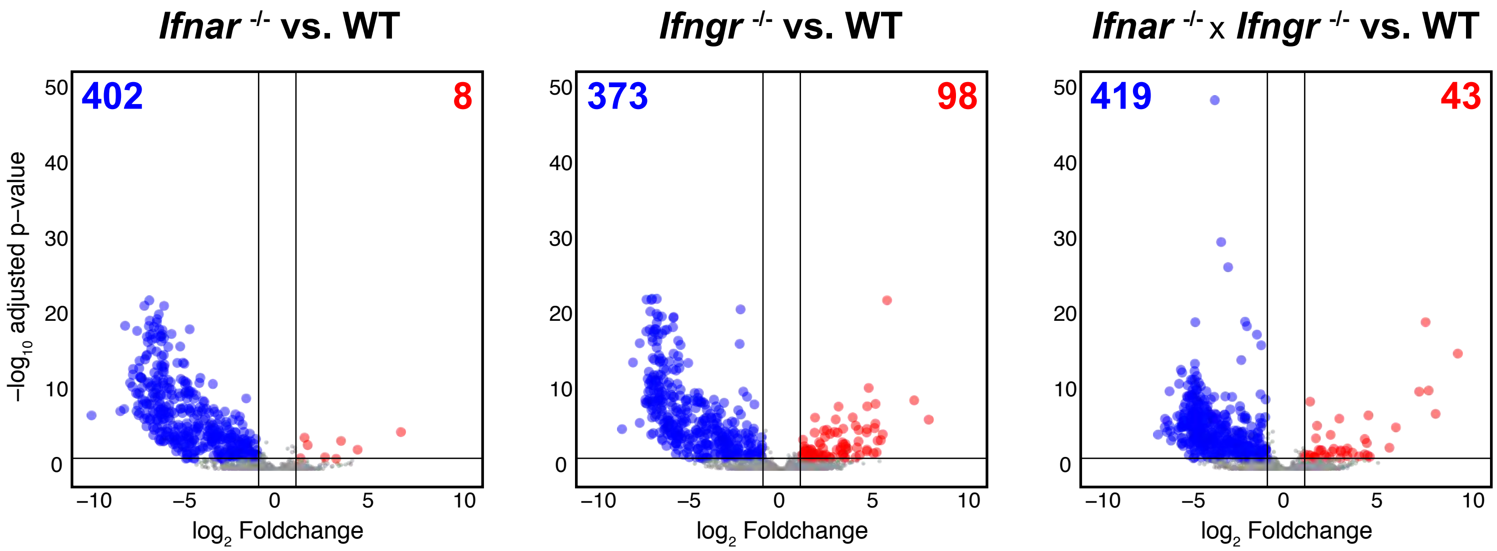

b

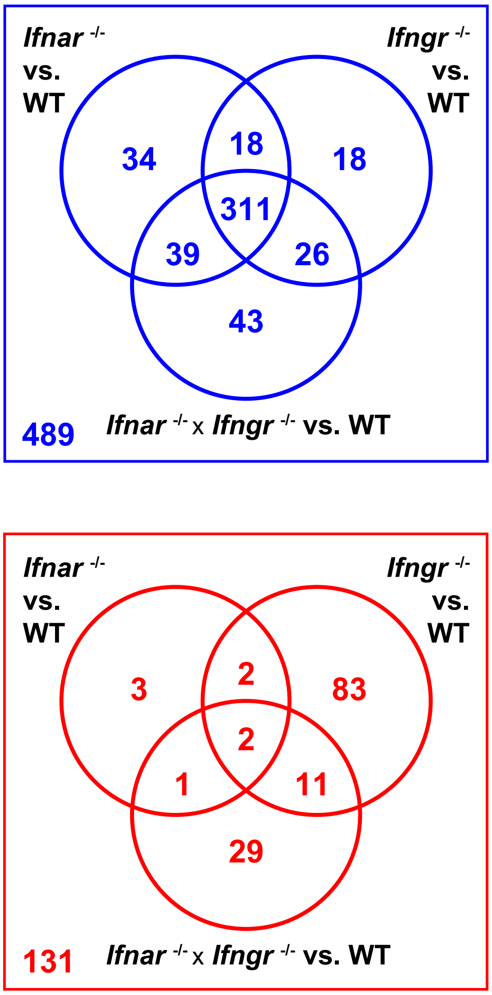

c

Module	Down regulated genes in each module	FDR p-val	Up regulated genes in each module	FDR p-val	Total genes in module
L1	0	1	1	0.42	78
L2	1	1	1	0.86	192
L3	4	1	5	0.34	370
L4	0	1	0	1	175
L5	31	1.8E-15	0	1	235
L6	5	1	6	0.84	734
L7	38	1.4E-17	0	1	309
L8	3	1	3	0.12	134
L9	1	1	0	0.86	79
L10	6	0.23	9	5.1E-06	143
L11	8	1.3E-02	1	0.65	121
L12	8	1	4	0.42	318
L13	3	1	5	8.5E-03	135
L14	4	1	4	0.42	330
L15	2	1	0	1	337
L16	1	1	0	1	115
L17	4	0.37	1	0.6	104
L18	3	1	1	1	492
L19	1	1	0	1	156
L20	5	1	0	1	418
L21	1	1	0	0.84	61
L22	5	1	5	0.06	223
L23	3	0.49	0	0.86	85
L24	3	1	9	1.9E-02	433
L25	5	0.23	13	1.9E-11	116
L26	10	8.6E-04	6	8.0E-04	121
L27	6	1	1	1	760
L28	5	1	0	1	302
L29	0	1	0	1	323
L30	3	1	0	1	289
L31	2	1	0	1	243
L32	1	1	1	0.77	144
L33	4	1	0	1	723
L34	8	1	1	1	670
L35	3	1	0	1	212
L36	0	1	0	0.86	84
L37	1	1	5	8.9E-03	142
L38	33	1.1E-54	0	0.65	35

Supplementary Figure 11

Supplementary Figure 11. Tonic activity of Type I and II IFN signaling in lung. a, Volcano plots depicting the differentially expressed genes between each of the IFN receptor knockout (KO) controls compared to Wild type (WT) controls in lung samples from the Toxoplasma WT and IFN receptor KO dataset. Significantly differentially expressed genes ($\log_2$ fold change >1 or <−1, and FDR p-value < 0.05) are represented as red (up-regulated) or blue (down-regulated) dots. The numbers of down- (in blue) or up-regulated (in red) genes are listed in the volcano plot. **b,** Venn diagrams showing the overlap between the down- (in blue) or up-regulated (in red) genes from the three comparisons. The number of genes involved in tonic signaling (i.e. the sum of all unique differentially expressed genes within the Venn diagram) is indicated on the bottom left corner of each Venn diagram. **c,** The numbers of down- (in blue) or up-regulated (in red) genes involved in tonic signaling within each lung module. The total number of genes within each lung module is also shown. Significance was calculated using a hypergeometric test, with FDR p-values shown here.

a **IFN receptor KO controls compared to Wildtype controls in Blood**

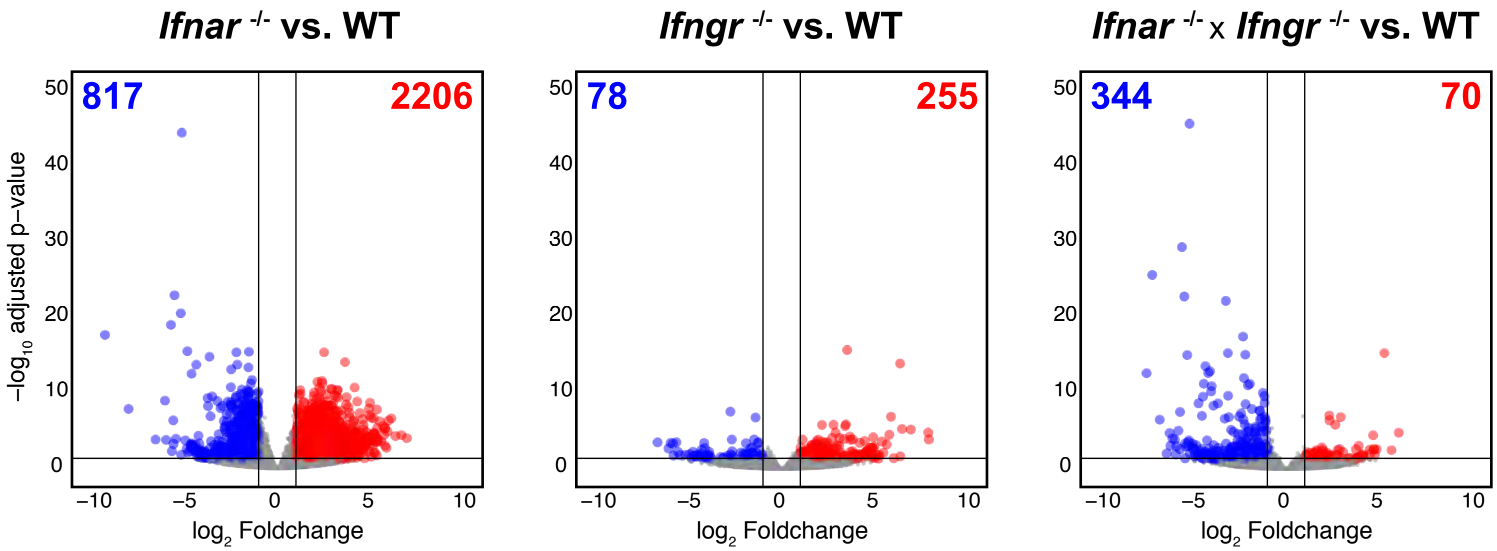

b

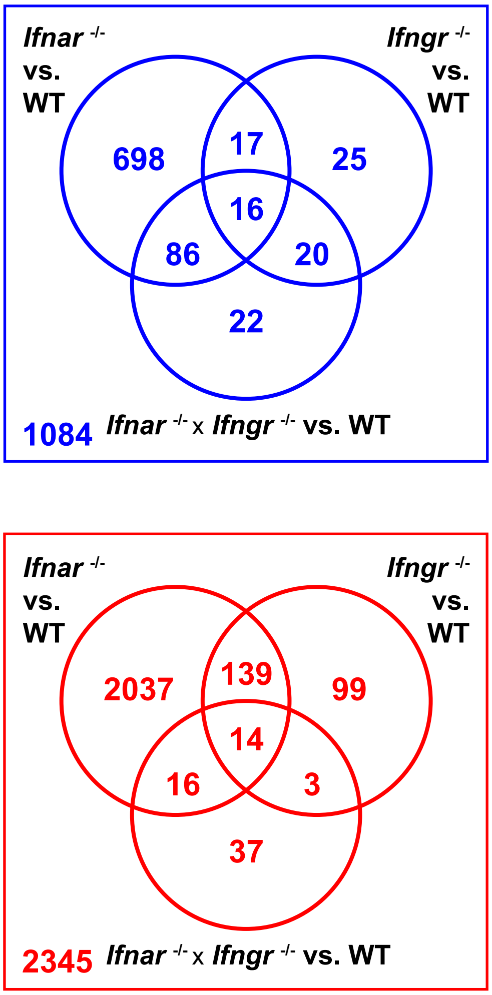

c

Module	Down regulated genes in each module	FDR p-val	Up regulated genes in each module	FDR p-val	Total genes in module
L1	2	1	1	1	78
L2	7	1	0	1	192
L3	19	1	9	1	370
L4	7	1	0	1	175
L5	53	2.0E-18	7	1	235
L6	43	0.92	80	1	734
L7	50	1.8E-11	9	1	309
L8	4	1	8	1	134
L9	10	4.9E-02	4	1	79
L10	4	1	21	1	143
L11	6	1	24	0.99	121
L12	18	1	46	1	318
L13	11	0.38	13	1	135
L14	12	1	14	1	330
L15	20	0.92	14	1	337
L16	5	1	1	1	115
L17	6	0.97	32	4.3E-03	104
L18	17	1	44	1	492
L19	5	1	28	1	156
L20	16	1	139	2.0E-12	418
L21	4	0.86	13	0.81	61
L22	7	1	145	3.0E-52	223
L23	7	0.46	25	2.2E-02	85
L24	7	1	84	0.99	433
L25	0	1	4	1	116
L26	10	0.38	13	1	121
L27	34	1	195	2.9E-06	760
L28	21	0.47	89	9.1E-06	302
L29	10	1	36	1	323
L30	15	1	131	3.7E-25	289
L31	21	0.14	123	5.1E-29	243
L32	7	1	55	7.6E-08	144
L33	37	1	258	3.9E-28	723
L34	32	1	200	2.4E-12	670
L35	14	0.74	4	1	212
L36	0	1	3	1	84
L37	2	1	9	1	142
L38	1	1	0	1	35

Supplementary Figure 12

Supplementary Figure 12. Tonic activity of Type I and II IFN signaling in blood. **a,** Volcano plots depicting the differentially expressed genes between each of the IFN receptor knockout (KO) controls compared to Wild type (WT) controls in blood samples from the Toxoplasma WT and IFN receptor KO dataset. Significantly differentially expressed genes (log_2 fold change >1 or <−1, and FDR p-value < 0.05) are represented as red (up-regulated) or blue (down-regulated) dots. The numbers of down- (in blue) or up-regulated (in red) genes are listed in the volcano plot. **b,** Venn diagrams showing the overlap between the down- (in blue) or up-regulated (in red) genes from the three comparisons. The number of genes involved in tonic signaling (i.e. the sum of all unique differentially expressed genes within the Venn diagram) is indicated on the bottom left corner of each Venn diagram. **c,** The numbers of down- (in blue) or up-regulated (in red) genes involved in tonic signaling within each lung module. The total number of genes within each lung module is also shown. Significance was calculated using a hypergeometric test, with FDR p-values shown here.

Differentially expressed genes in the Lung IFN-associated modules between IFN receptor KO controls and Wildtype controls in Blood

Downregulated genes in IFN receptor KO controls compared to Wildtype controls

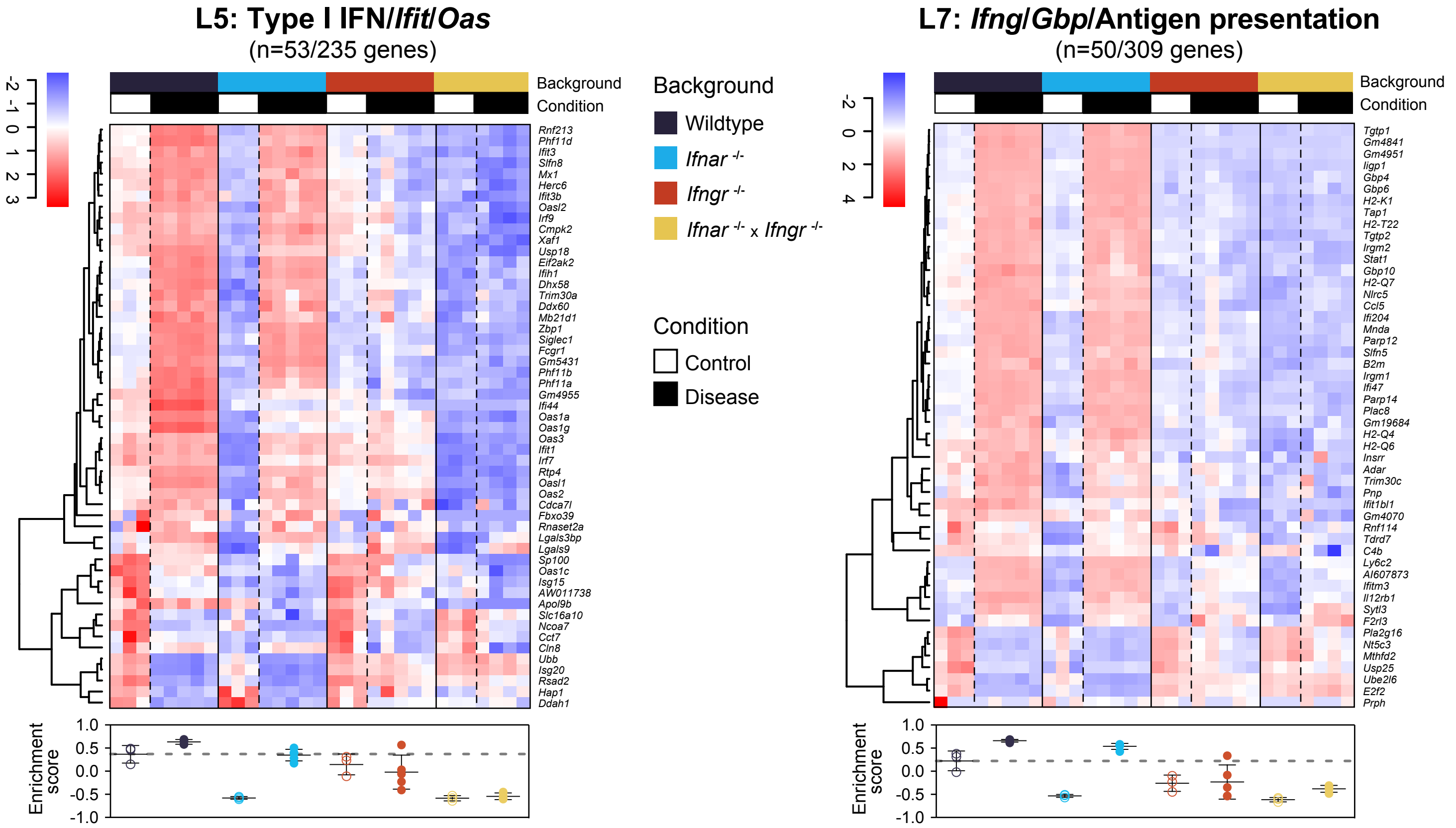

Supplementary Figure 13

Supplementary Figure 13. Genes involved in the tonic Type I and II IFN signaling in the lung IFN-associated modules in blood samples. Heatmaps depicting the log_2 gene expression values of the differentially expressed genes between IFN receptor KO controls compared to the Wild type controls in blood in the L5 (Type I IFN/*Ifit*/*Oas*) and L7 (*Ifng*/*Gbp*/Antigen presentation) modules (Supplementary Figure 12). Gene expression values were averaged and scaled across the row to indicate the number of standard deviations above (red) or below (blue) the mean, denoted as row Z-score. Dendrogram shows unsupervised hierarchical clustering of genes, with distances calculated using Pearson correlation and clustered using the complete linkage. Enrichment scores calculated on a single-sample basis using GSVA are shown for the differentially expressed genes below the heatmap for each module. Empty and filled circles represent control and disease samples, respectively, and color represents Wild type and IFN receptor KO mice. Mean and standard deviation for each group are shown, with a dashed line indicating the mean of the Wild type control group.

a

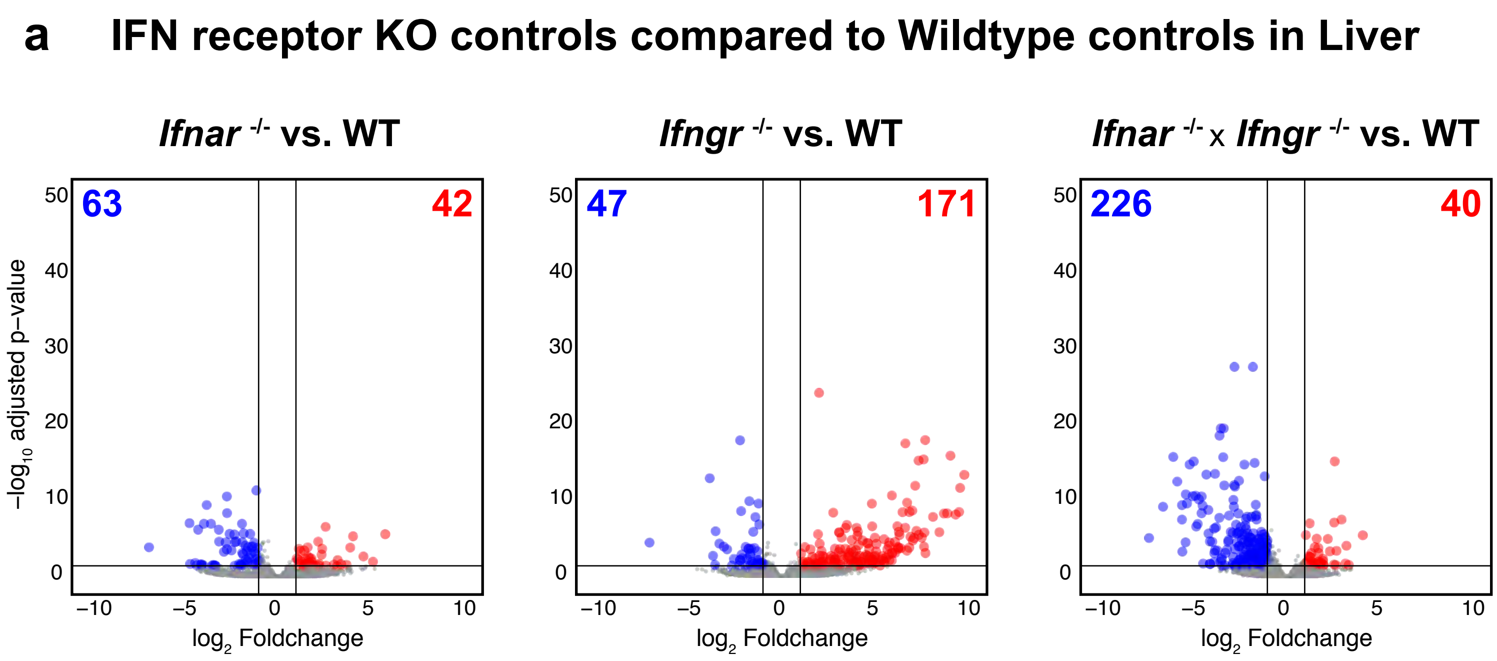

b

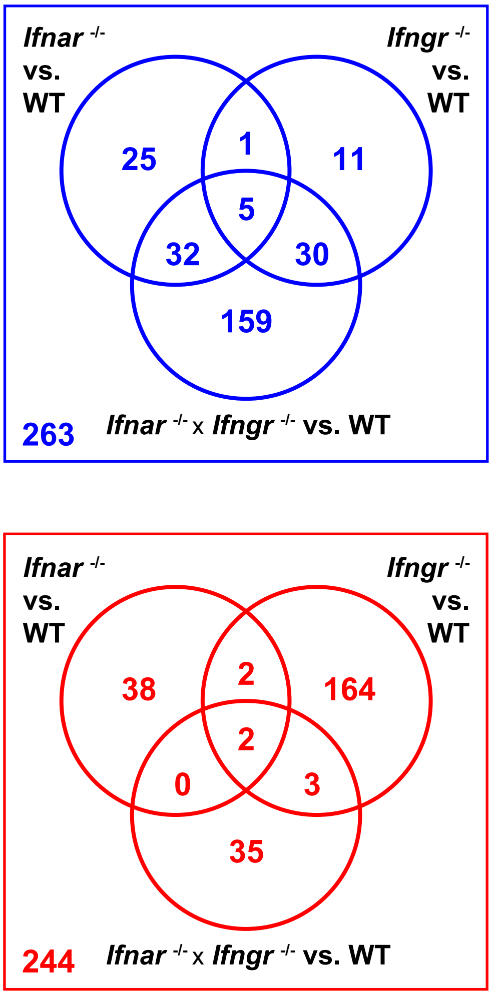

c

Module	Down regulated genes in each module	FDR p-val	Up regulated genes in each module	FDR p-val	Total genes in module
L1	4	0.21	1	1	78
L2	1	1	0	1	192
L3	18	7.8E-03	3	1	370
L4	0	1	0	1	175
L5	48	1.2E-32	2	1	235
L6	21	0.63	16	1	734
L7	58	3.5E-37	1	1	309
L8	0	1	0	1	134
L9	0	1	2	1	79
L10	0	1	3	1	143
L11	4	0.7	3	1	121
L12	3	1	7	1	318
L13	1	1	4	1	135
L14	6	1	2	1	330
L15	5	1	2	1	337
L16	1	1	0	1	115
L17	2	1	2	1	104
L18	1	1	5	1	492
L19	1	1	3	1	156
L20	1	1	7	1	418
L21	0	1	1	1	61
L22	0	1	50	4.6E-37	223
L23	1	1	4	0.47	85
L24	3	1	50	7.6E-23	433
L25	0	1	1	1	116
L26	0	1	5	0.47	121
L27	5	1	5	1	760
L28	2	1	3	1	302
L29	0	1	0	1	323
L30	1	1	2	1	289
L31	1	1	5	1	243
L32	0	1	2	1	144
L33	2	1	11	1	723
L34	1	1	13	1	670
L35	27	4.7E-13	1	1	212
L36	6	2.3E-02	0	1	84
L37	2	1	1	1	142
L38	1	0.82	0	1	35

Supplementary Figure 14

Supplementary Figure 14. Tonic activity of Type I and II IFN signaling in liver. a, Volcano plots depicting the differentially expressed genes between each of the IFN receptor knockout (KO) controls compared to Wild type (WT) controls in liver samples from the Toxoplasma WT and IFN receptor KO dataset. Significantly differentially expressed genes (log_2 fold change >1 or <−1, and FDR p-value < 0.05) are represented as red (up-regulated) or blue (down-regulated) dots. The numbers of down- (in blue) or up-regulated (in red) genes are listed in the volcano plot. **b,** Venn diagrams showing the overlap between the down- (in blue) or up-regulated (in red) genes from the three comparisons. The number of genes involved in tonic signaling (i.e. the sum of all unique differentially expressed genes within the Venn diagram) is indicated on the bottom left corner of each Venn diagram. **c,** The numbers of down- (in blue) or up-regulated (in red) genes involved in tonic signaling within each lung module. The total number of genes within each lung module is also shown. Significance was calculated using a hypergeometric test, with FDR p-values shown here.

Differentially expressed genes in the Lung IFN-associated modules between IFN receptor KO controls and Wildtype controls in Liver

Downregulated genes in IFN receptor KO controls compared to Wildtype controls

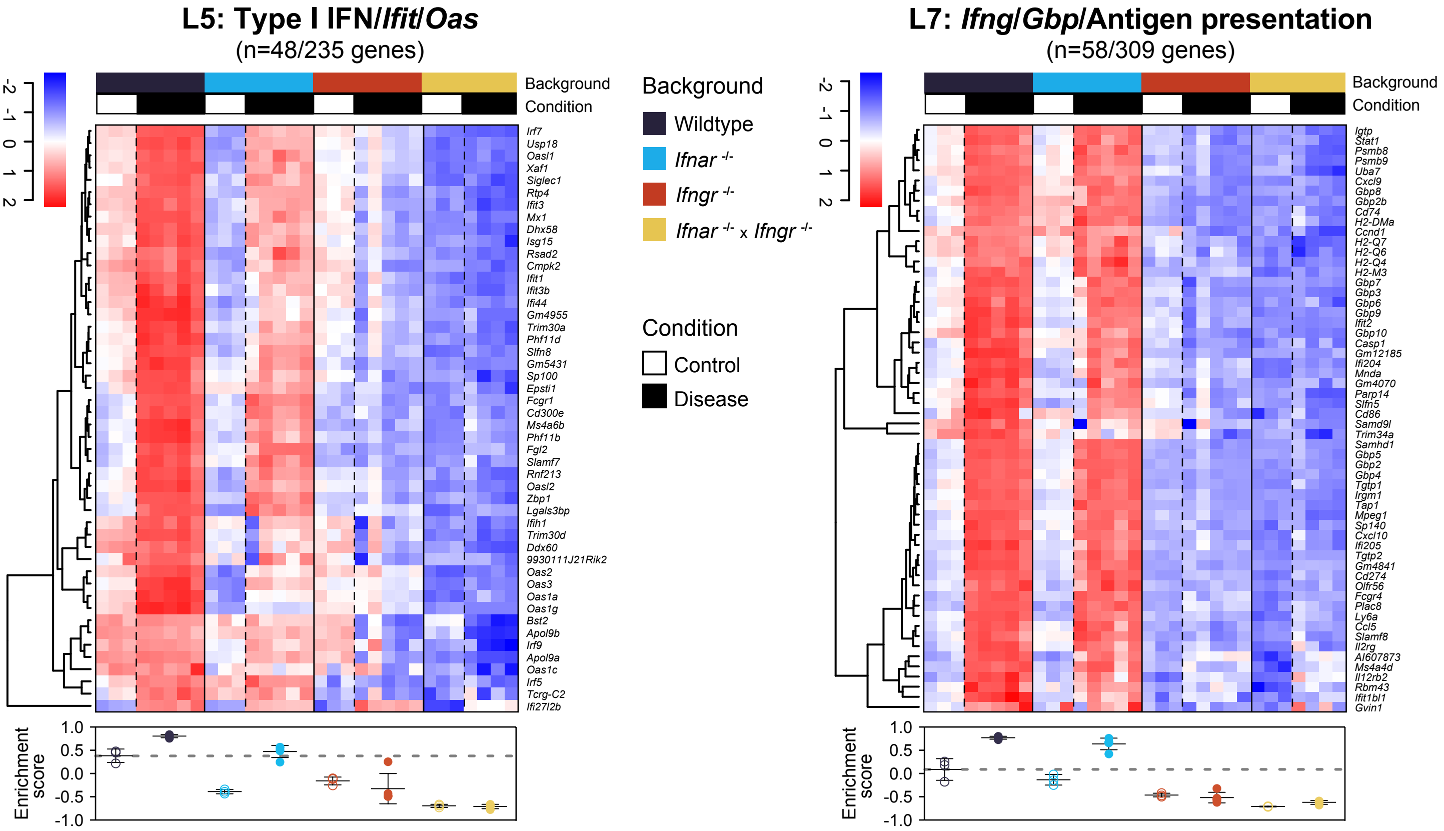

Supplementary Figure 15

Supplementary Figure 15. Genes involved in the tonic Type I and II IFN signaling in the lung IFN-associated modules in liver samples. Heatmaps depicting the $\log_2$ gene expression values of the differentially expressed genes between IFN receptor KO controls compared to the Wild type controls in liver in the L5 (Type I IFN/*Ifit*/*Oas*) and L7 (*Ifng*/*Gbp*/Antigen presentation) modules (Supplementary Figure 14). Gene expression values were averaged and scaled across the row to indicate the number of standard deviations above (red) or below (blue) the mean, denoted as row Z-score. Dendrogram shows unsupervised hierarchical clustering of genes, with distances calculated using Pearson correlation and clustered using the complete linkage. Enrichment scores calculated on a single-sample basis using GSVA are shown for the differentially expressed genes below the heatmap for each module. Empty and filled circles represent control and disease samples, respectively, and color represents Wild type and IFN receptor KO mice. Mean and standard deviation for each group are shown, with a dashed line indicating the mean of the Wild type control group.

a IFN receptor KO controls compared to Wildtype controls in Spleen

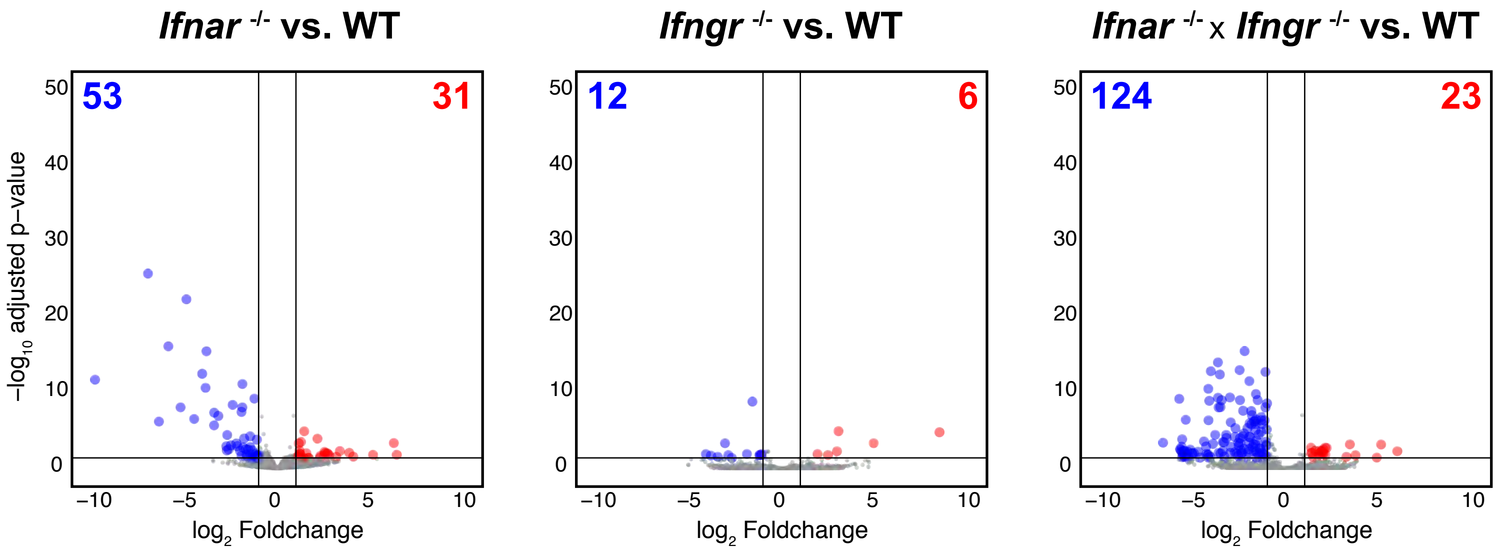

b

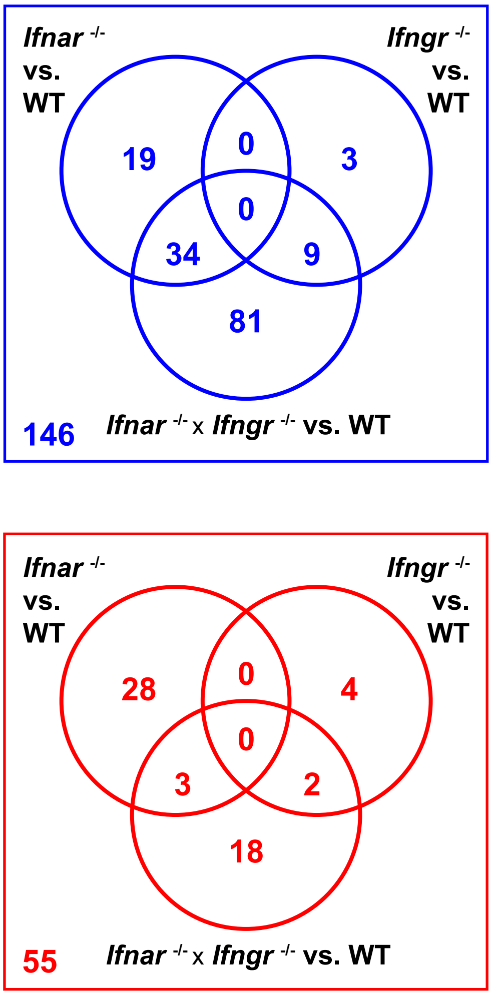

c

Module	Down regulated genes in each module	FDR p-val	Up regulated genes in each module	FDR p-val	Total genes in module
L1	1	1	0	0.6	78
L2	0	1	0	0.7	192
L3	0	1	1	0.64	370
L4	0	1	1	0.46	175
L5	32	4.7E-26	1	0.6	235
L6	10	1	0	0.95	734
L7	41	5.8E-33	1	0.63	309
L8	1	1	2	0.14	134
L9	0	1	1	0.2	79
L10	2	1	5	4.0E-04	143
L11	1	1	1	0.3	121
L12	0	1	7	4.0E-04	318
L13	0	1	4	1.9E-03	135
L14	0	1	0	0.83	330
L15	0	1	1	0.63	337
L16	1	1	0	0.63	115
L17	0	1	1	0.29	104
L18	0	1	0	0.88	492
L19	0	1	0	0.65	156
L20	1	1	4	0.15	418
L21	1	1	1	0.15	61
L22	1	1	0	0.72	223
L23	0	1	0	0.6	85
L24	4	1	0	0.86	433
L25	2	1	1	0.3	116
L26	0	1	0	0.63	121
L27	3	1	1	0.86	760
L28	2	1	0	0.83	302
L29	0	1	0	0.83	323
L30	1	1	1	0.62	289
L31	2	1	1	0.6	243
L32	1	1	0	0.64	144
L33	0	1	1	0.86	723
L34	6	1	2	0.66	670
L35	2	1	0	0.72	212
L36	0	1	0	0.6	84
L37	0	1	0	0.64	142
L38	0	1	0	0.44	35

Supplementary Figure 16

Supplementary Figure 16. Tonic activity of Type I and II IFN signaling in spleen. **a,** Volcano plots depicting the differentially expressed genes between each of the IFN receptor knockout (KO) controls compared to Wild type (WT) controls in spleen samples from the Toxoplasma WT and IFN receptor KO dataset. Significantly differentially expressed genes (log_2 fold change >1 or <−1, and FDR p-value < 0.05) are represented as red (up-regulated) or blue (down-regulated) dots. The numbers of down- (in blue) or up-regulated (in red) genes are listed in the volcano plot. **b,** Venn diagrams showing the overlap between the down- (in blue) or up-regulated (in red) genes from the three comparisons. The number of genes involved in tonic signaling (i.e. the sum of all unique differentially expressed genes within the Venn diagram) is indicated on the bottom left corner of each Venn diagram. **c,** The numbers of down- (in blue) or up-regulated (in red) genes involved in tonic signaling within each lung module. The total number of genes within each lung module is also shown. Significance was calculated using a hypergeometric test, with FDR p-values shown here.

Differentially expressed genes in the Lung IFN-associated modules between IFN receptor KO controls and Wildtype controls in Spleen

Downregulated genes in IFN receptor KO controls compared to Wildtype controls

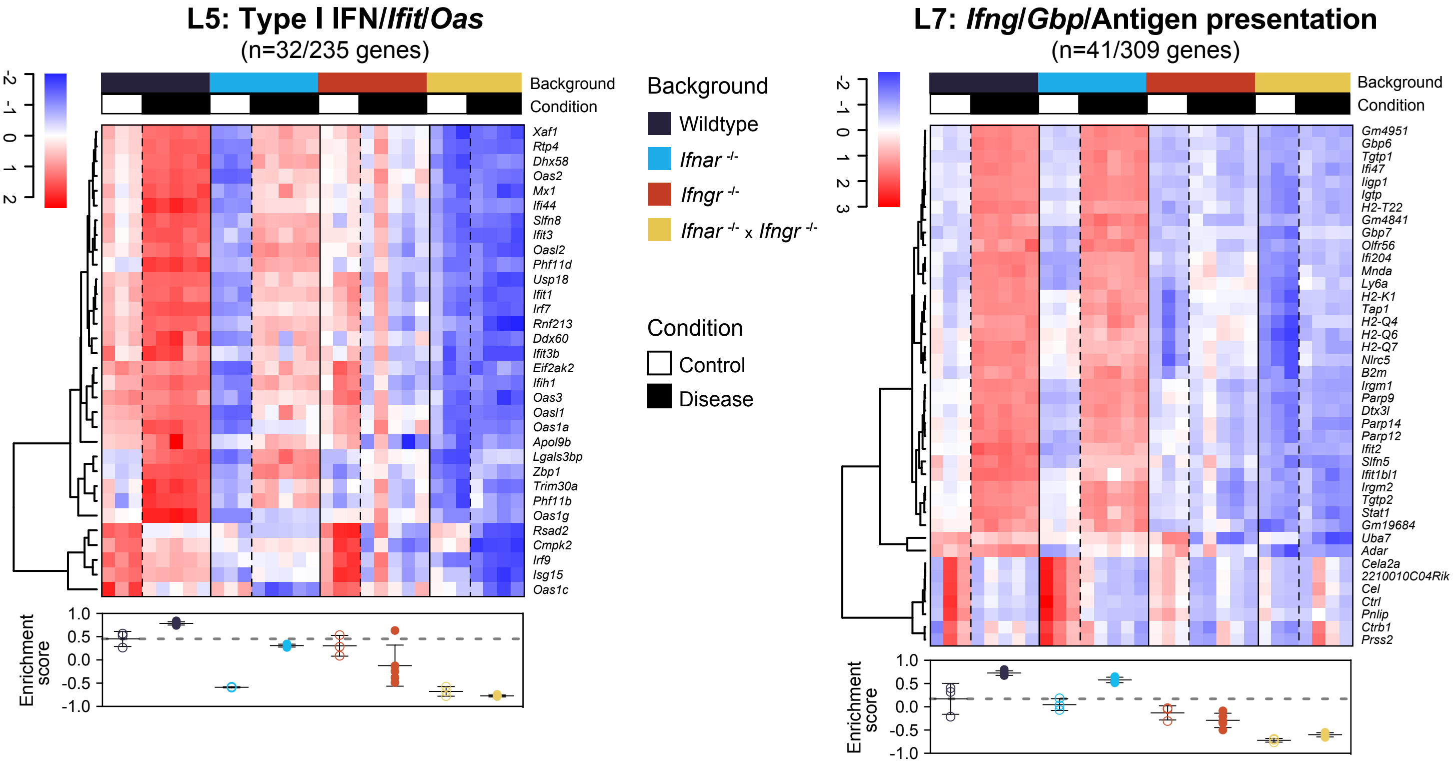

Supplementary Figure 17

Supplementary Figure 17. Genes involved in the tonic Type I and II IFN signaling in the lung IFN-associated modules in spleen samples. Heatmaps depicting the log_2 gene expression values of the differentially expressed genes between IFN receptor KO controls compared to the Wild type controls in spleen in the L5 (Type I IFN/*Ifit*/*Oas*) and L7 (*Ifng*/*Gbp*/Antigen presentation) modules (Supplementary Figure 12). Gene expression values were averaged and scaled across the row to indicate the number of standard deviations above (red) or below (blue) the mean, denoted as row Z-score. Dendrogram shows unsupervised hierarchical clustering of genes, with distances calculated using Pearson correlation and clustered using the complete linkage. Enrichment scores calculated on a single-sample basis using GSVA are shown for the differentially expressed genes below the heatmap for each module. Empty and filled circles represent control and disease samples, respectively, and color represents Wild type and IFN receptor KO mice. Mean and standard deviation for each group are shown, with a dashed line indicating the mean of the Wild type control group.

L7: *Ifng*/*Gbp*/Antigen presentation
(n=43/309 genes)

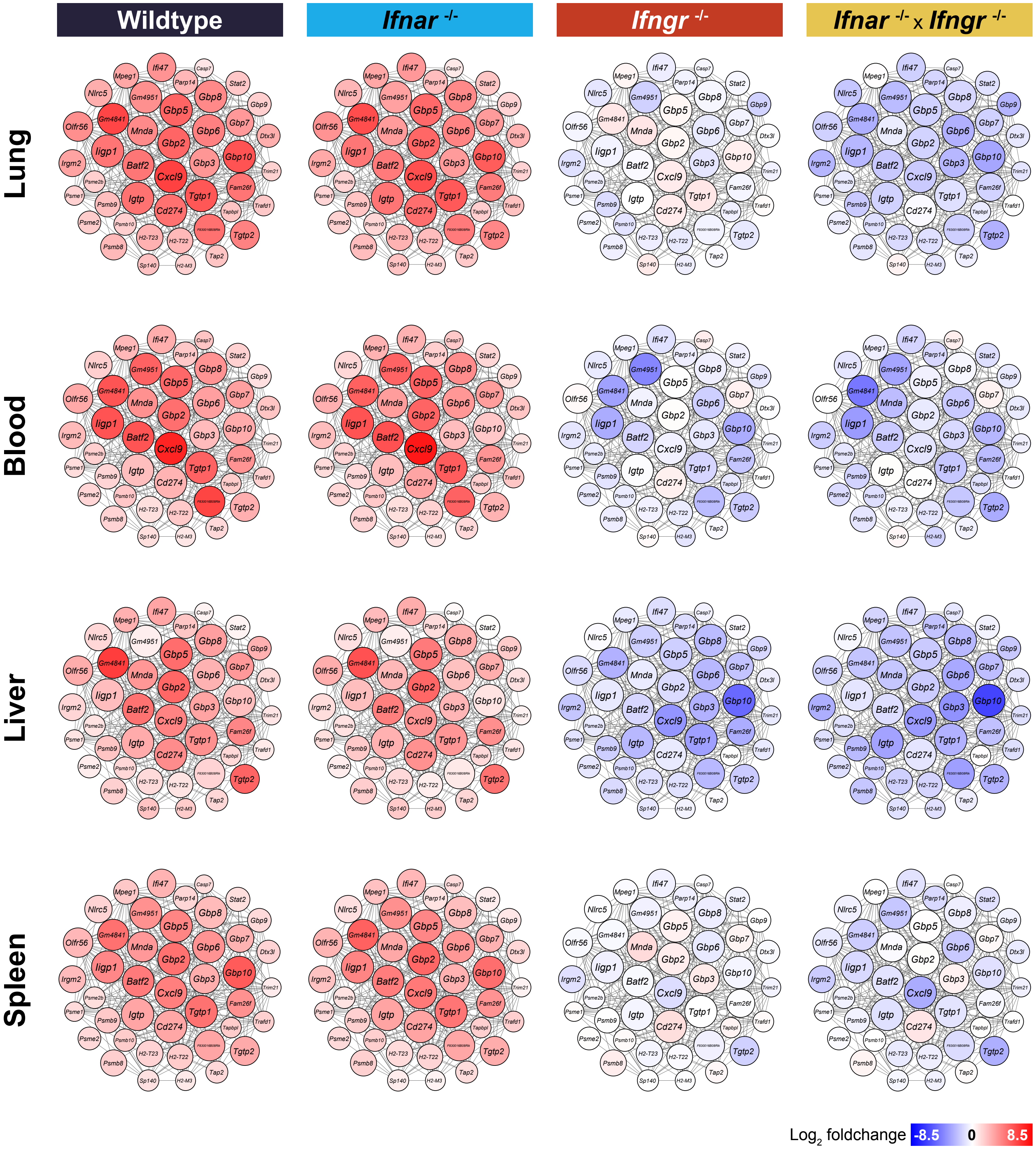

Supplementary Figure 18

Supplementary Figure 18. Type II, but not type I, IFN signaling regulates the expression of genes within the lung *Ifng*/*Gbp*/Antigen presentation (L7) module following *T. gondii* infection. Gene networks depicting the 'hub' genes in the lung L7 module representing genes with high intramodular connectivity, i.e. genes most connected with all other genes within the module. Each gene is represented as a circular node with edges representing correlation between the gene expression profiles of the two respective genes. Color of the node represents $\log_2$ foldchange of the gene for Wild type or IFN receptor knockout *T. gondii* infected mice compared to Wild type controls, across lung, blood, liver and spleen.

Histology

a

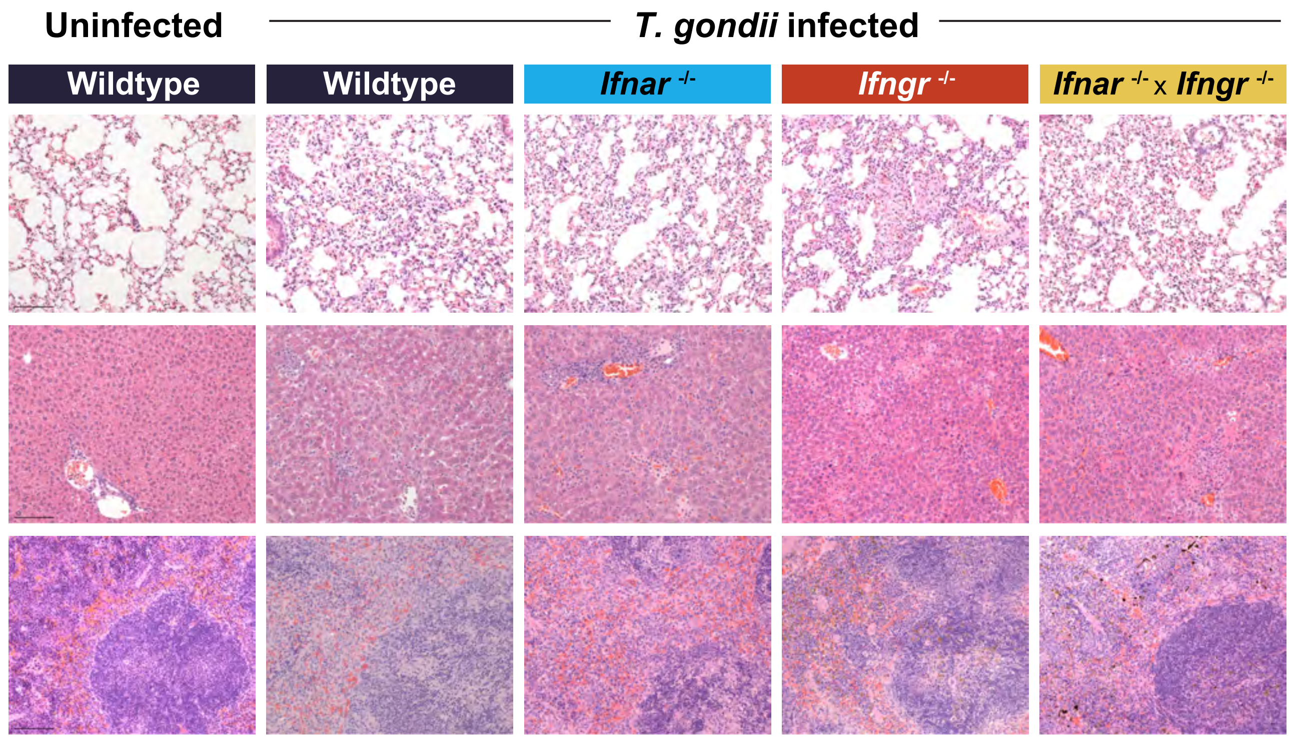

b

Tissue	Histological parameter	Descriptors	Wildtype					*Ifnar* -/-					*Ifngr* -/-					*Ifnar* -/- x *Ifngr* -/-			
Lung	Inflammation	Granulocytes	1	1	1	1	1	2	1	2	2	1	2	1	1	1	2	2	1	1	1
		Mononuclear cells	1	1	1	1	1	2	1	2	1	1	2	1	1	1	2	1	1	2	2
	Necrosis		0	1	1	1	1	1	1	0	0	0	2	1	1	1	2	0	0	1	1
Liver	Inflammation	Granulocytes	1	1	1	0	0	1	2	2	2	2	2	1	1	1	1	2	1	1	1
		Mononuclear cells	2	2	1	1	1	2	2	2	2	2	1	1	0	0	1	2	1	1	1
	Necrosis		1	1	1	1	1	2	2	1	1	1	2	1	2	2	2	1	1	2	2
	Thrombosis + Coagulative necrosis		0	0	P	0	0	P	P	P	P	P	P	0	P	0	0	0	0	0	0
Spleen	Inflammation	Granulocytes	1	1	1	1	1	2	2	2	2	2	1	0	0	0	0	2	2	2	2
		Mononuclear cells	2	2	2	2	2	1	1	1	1	1	2	0	2	2	2	1	1	1	1
	Necrosis		1	1	1	1	1	2	2	2	2	2	1	0	1	1	1	0	0	0	0

Semi-quantitative scoring: 0 = no lesions; 1 = mild change; 2 = moderate/marked change; P = present.

Mouse samples mapped to *Toxoplasma gondii* genome

c

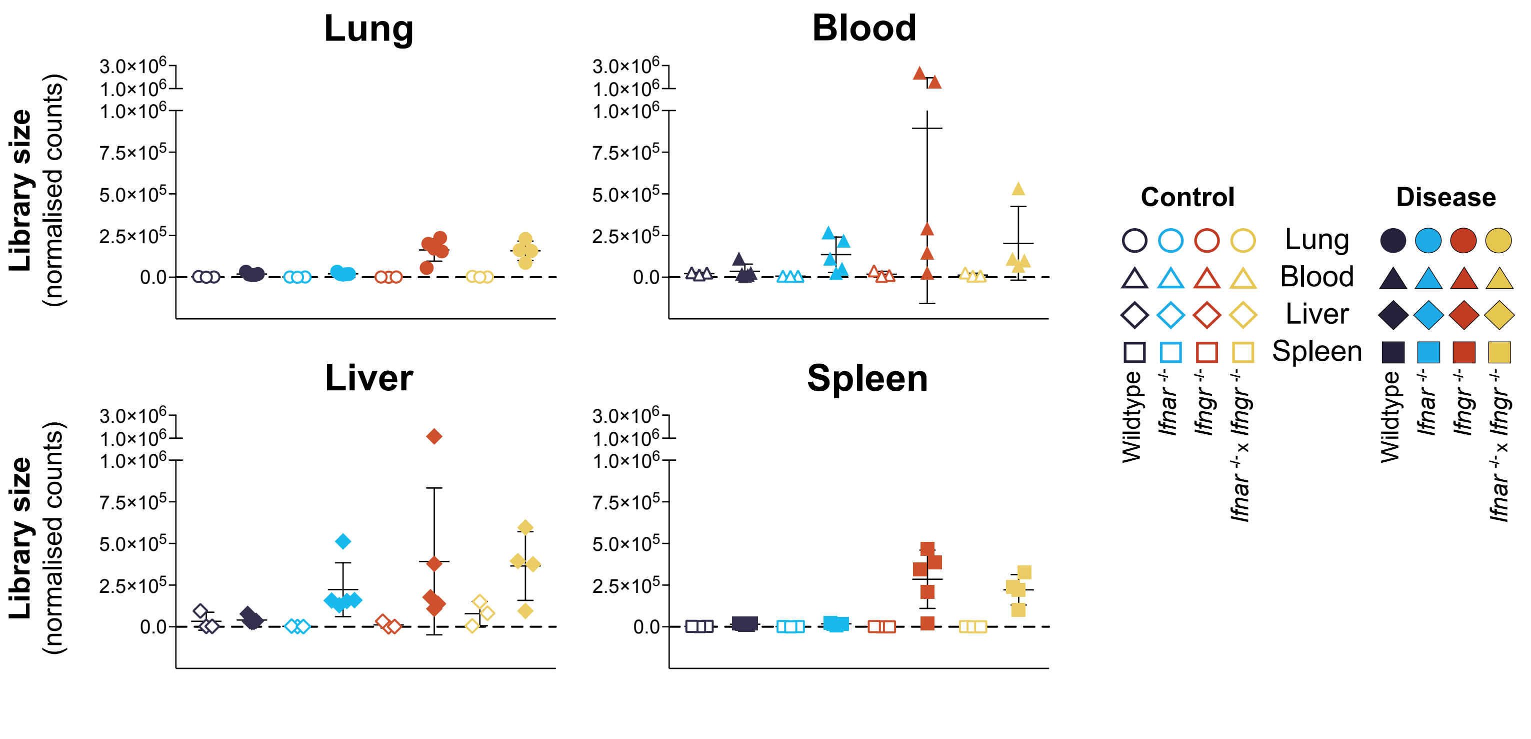

Supplementary Figure 19

Supplementary Figure 19. Type I and II IFN signaling are required to control pathology and parasite load during infection with *T. gondii*. a, Representative photomicrographs of hematoxylin and eosin (H&E) stained lung, liver and spleen from Wild type uninfected controls and Wild type, *Ifnar*$^{-/-}$, *Ifngr*$^{-/-}$ and double *Ifnar*$^{-/-}$ x *Ifngr*$^{-/-}$ mice infected with *T. gondii* (n=3-5 mice per group). Bar = 100µm**. b,** Lung, liver and spleen sections from each mouse were scored for relative severity of histological changes by a board-certified veterinary pathologist. **c,** Library size of the normalised counts for control and *T. gondii* infected RNA-seq samples from Wild type and IFN receptor KO mice, mapped to the *T. gondii* genome. Shape represents the different tissues, empty and filled symbols represent control and disease samples, respectively, and the color of the symbol represents Wild type and IFN receptor KO mice.

Lung modules assessed in lung and blood samples from mice infected with *T. gondii* in different animal houses

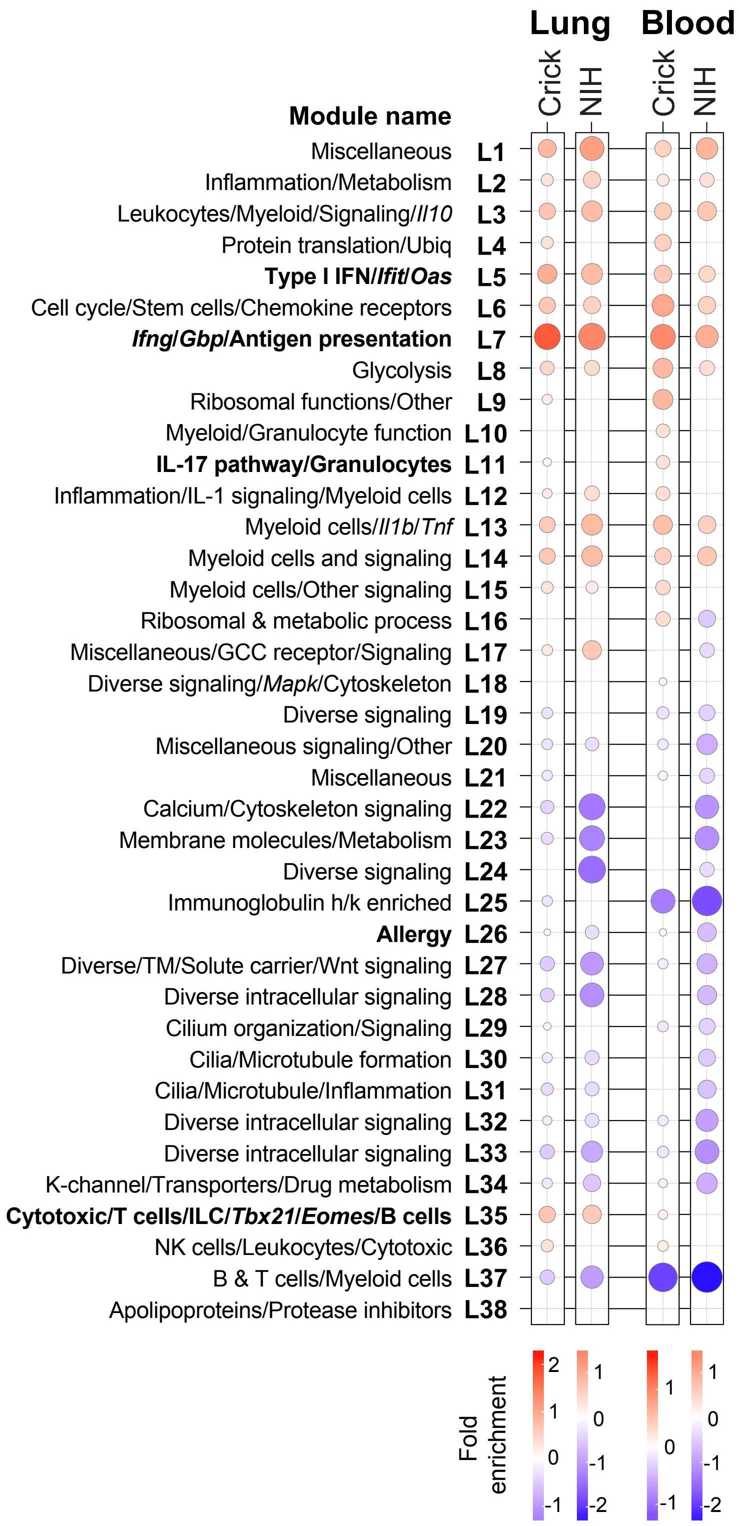

Supplementary Figure 20

Supplementary Figure 20. Lung modules assessed in lung and blood of samples from Wild type mice infected with *T. gondii* in different animal houses. Modular transcriptional profiles of lung and blood tissues from *T. gondii* infected, either at the Crick or at NIH, Wild type C57BL/6 mice as assessed using the lung modules. Red and blue circles indicate the cumulative over- or under-abundance of all genes within the module for each *T. gondii* infected Wild type group compared to their respective uninfected Wild type controls in each tissue. Color intensity of the dots represents the degree of perturbation, indicated by the color scale. Size of the dots represents the relative degree of perturbation, with the largest dot representing the highest degree of perturbation within the plot. Within each group, only modules with FDR p-value < 0.05 were considered significant and depicted here. GCC, glucocorticoid; K-channel, potassium channel; TM, transmembrane; Ubiq, ubiquitination.
