# Supplementary material for: Transcriptional profiling unveils type I and II interferon networks in blood and tissues across diseases
Source: Nat Commun. 2019 Jun 28;10:2887. doi: 10.1038/s41467-019-10601-6 (PMC6599044; doi:10.1038/s41467-019-10601-6)
Supplement: Supplementary file 2 — Description of Additional Supplementary Files [file 41467_2019_10601_MOESM2_ESM.pdf]

Description of Additional Supplementary Files

File Name: Supplementary Data 1
Description: Number of samples in each group and number of differentially expressed genes

File Name: Supplementary Data 2
Description: Genes in the lung modules with average normalised read counts for each group

File Name: Supplementary Data 3
Description: Genes in the blood modules with average normalised read counts for each group

File Name: Supplementary Data 4
Description: Annotation of the lung modules

File Name: Supplementary Data 5
Description: Annotation of the blood modules

File Name: Supplementary Data 6
Description: Cell-type specific signatures derived from ImmGen ULI RNA-seq dataset

File Name: Supplementary Data 7
Description: In vitro derived T helper cell sigantures

File Name: Supplementary Data 8
Description: Preservation of lung modules in blood

File Name: Supplementary Data 9
Description: Preservation of blood modules in lung

File Name: Supplementary Data 10
Description: Differential gene expression in BAL and blood samples from a test HDM allergy model (nasal sensitization)

File Name: Supplementary Data 11
Description: Differential gene expression in sorted cells from lung and blood from HDM allergy model

File Name: Supplementary Data 12
Description: Differential gene expression in lung samples from mouse models of infectious and inflammatory diseases

File Name: Supplementary Data 13
Description: Differential gene expression in blood samples from mouse models of infectious and inflammatory diseases

File Name: Supplementary Data 14

Description: Average normalised read counts for all genes in control and Toxoplasma gondii infected Wildtype and IFN receptor KO mice

File Name: Supplementary Data 15
Description: Differential gene expression in uninfected (control) and Toxoplasma gondii infected (disease) Wildtype and IFN receptor KO mice in lung

File Name: Supplementary Data 16
Description: Differential gene expression in control and Toxoplasma gondii infected Wildtype and IFN receptor KO mice in blood

File Name: Supplementary Data 17
Description: Differential gene expression in control and Toxoplasma gondii infected Wildtype and IFN receptor KO mice in liver

File Name: Supplementary Data 18
Description: Differential gene expression in control and Toxoplasma gondii infected Wildtype and IFN receptor KO mice in spleen

File Name: Supplementary Data 19
Description: Summary of Interferome Data in modules: type I and type II and overlapping number of genes

File Name: Supplementary Data 20
Description: Information on the Quality and Quantity of RNA samples
