# Supplementary material for: Transcriptional profiling unveils type I and II interferon networks in blood and tissues across diseases
Source: Nat Commun. 2019 Jun 28;10:2887. doi: 10.1038/s41467-019-10601-6 (PMC6599044; doi:10.1038/s41467-019-10601-6)
Supplement: Supplementary file 23 — Reporting summary [file 41467_2019_10601_MOESM23_ESM.pdf]

nature research

Corresponding author(s): Dr. Anne O'Garra

Last updated by author(s): Jan 23, 2019

Reporting Summary

Nature Research wishes to improve the reproducibility of the work that we publish. This form provides structure for consistency and transparency in reporting. For further information on Nature Research policies, see Authors & Referees and the Editorial Policy Checklist.

Statistics

For all statistical analyses, confirm that the following items are present in the figure legend, table legend, main text, or Methods section.

n/a	Confirmed	
☐	☒	The exact sample size (*n*) for each experimental group/condition, given as a discrete number and unit of measurement
☐	☒	A statement on whether measurements were taken from distinct samples or whether the same sample was measured repeatedly
☐	☒	The statistical test(s) used AND whether they are one- or two-sided<br>*Only common tests should be described solely by name; describe more complex techniques in the Methods section.*
☒	☐	A description of all covariates tested
☐	☒	A description of any assumptions or corrections, such as tests of normality and adjustment for multiple comparisons
☐	☒	A full description of the statistical parameters including central tendency (e.g. means) or other basic estimates (e.g. regression coefficient) AND variation (e.g. standard deviation) or associated estimates of uncertainty (e.g. confidence intervals)
☐	☒	For null hypothesis testing, the test statistic (e.g. *F*, *t*, *r*) with confidence intervals, effect sizes, degrees of freedom and *P* value noted<br>*Give P values as exact values whenever suitable.*
☒	☐	For Bayesian analysis, information on the choice of priors and Markov chain Monte Carlo settings
☒	☐	For hierarchical and complex designs, identification of the appropriate level for tests and full reporting of outcomes
☐	☒	Estimates of effect sizes (e.g. Cohen's *d*, Pearson's *r*), indicating how they were calculated

Our web collection on statistics for biologists contains articles on many of the points above.

Software and code

Policy information about availability of computer code

Data collection	Flow cytometry data were collected using the BD FACSDiva software version 8.0.1 on a FACSAria™ Fusion and Summit V5.4.0 software on a MoFlo XDP (Beckman Coulter).<br><br>Histology images were captured on an Olympus BX43 microscope using Micropix Cytocam software v1.6.0.0.
Data analysis	No custom code or algorithm was used to generate results that are reported in this study. Only publicly available data analysis tools were utilized. All tools, including parameters that were used where necessary, are provided in the 'Online Materials and Methods' section that accompanies this manuscript. These tools included:<br><br>R (version 3.3.1) and bioconductor packages:<br>DESeq2 v1.12.4,<br>qusage v2.4.0,<br>clusterProfiler v3.0.5,<br>gsva,<br>limma v3.28.21<br><br>Other packages for RNA-seq anlaysis:<br>FastQC,<br>MultiQC,<br>Trimmomatic v0.36,<br>HISAT2 v2.0.4,<br>HtSeq v0.6.1<br><br>Other data analysis tools:<br>GeneSpring GX v14.8,

CIBERSORT,
WGCNA,
Cytoscape
Ingenuity Pathway Analysis (IPA),
Metacore,
Gene ontology database,
Interferome database 2.0

GraphPad Prism Version 7 was also used to plot bar graphs, box and whisker graphs and line graphs as well as to perform experimental statistical analysis.

OlyVIA software was used to view and score histology slides.

The WebApp (http://ogarra.shinyapps.io/MouseModules/) was created using the shiny (v 1.2.0) package in R, and no other custom code or software was used for this.

For manuscripts utilizing custom algorithms or software that are central to the research but not yet described in published literature, software must be made available to editors/reviewers. We strongly encourage code deposition in a community repository (e.g. GitHub). See the Nature Research guidelines for submitting code & software for further information.

Data

Policy information about availability of data

All manuscripts must include a data availability statement. This statement should provide the following information, where applicable:

- Accession codes, unique identifiers, or web links for publicly available datasets
- A list of figures that have associated raw data
- A description of any restrictions on data availability

The materials, data, code and any associated protocols that support the findings of this study are available from the corresponding author upon request. The Microarray and RNA-seq datasets have been deposited in the NCBI Gene Expression Omnibus (GEO) database with the primary accession number GSE119856. These data are currently private (Reviewer access: https://www.ncbi.nlm.nih.gov/geo/query/acc.cgi?acc=GSE119856; token: yfgvqwqctfezngx) and will be made public upon publication of this manuscript. Publically available datasets used in this study include GSE109125 (sorted cells from Immunological Genome Project), GSE106464 (in vitro differentiated T helper cells) and GSE61106 (Burkholderia pseudomallei (acute) microarray).

Field-specific reporting

Please select the one below that is the best fit for your research. If you are not sure, read the appropriate sections before making your selection.

☒ Life sciences ☐ Behavioural & social sciences ☐ Ecological, evolutionary & environmental sciences

For a reference copy of the document with all sections, see nature.com/documents/nr-reporting-summary-flat.pdf

Life sciences study design

All studies must disclose on these points even when the disclosure is negative.

Sample size

For module derivation, the numbers of mice used as controls or infections/challenge, were pre-determined to provide statistical significance for development of the modular signatures across diseases. Given the exploratory nature of the study, an a priori estimate of sample sizes for laboratory animals was obtained using Mead's resource equation which does not require information on experimental variability and/or magnitude.

Mead's resource equation: E = N - B - T
E: is the degrees of freedom of the error component, and should be somewhere between 10 and 20
N: is the total number of individuals or units in the study (minus 1)
B: is the blocking component, representing environmental effects allowed for in the design (minus 1)
T: is the treatment component, corresponding to the number of treatment groups (including control group) being used, or the number of questions being asked (minus 1)

Solved equation: (10 to 20) = (11 to 21) - 0 - 1

T: 2 study groups per dataset , controls and disease i.e. (T = 1)
B: no differences in environment between groups (B = 0)
Based on that and the above equation (given E should be between 10 and 20), between 12 and 22 animals for each dataset. A rounded number of animals at the high end of the range (20, 10 controls and 10 disease animals) was chosen, taking into consideration the large number of variables being measured.

For sorted cell populations, we aimed to perform at least 3 biological replicates which reflected experimental relevance and sample availability, with statistical differences providing the rationale for sufficiency of the number of samples.

For T. gondii infection of IFN- deficient mice, animal sample size estimates were determined using previous and/or pilot studies using 4-5 animals per group and guided by the 3R principle.

Data exclusions	Figure 8d, one of Ifngr-/- was excluded from lung neutrophil enumeration as it was found to be an outlier. Exclusion criteria were not pre-established.
Replication	Unless otherwise stated, experimental replicates were included in analysis e.g. in RNA-Seq analysis with appropriate statistical methods applied. Where representative data were shown, the experimental findings were reproduced with similar results and summary showed.
Randomization	Animals were age and sex matched and randomly selected into experimental groups.
Blinding	Blinding was not performed during animal studies. Analyses of H&E-stained sections of lung, liver and spleen sections were single-blinded.

Reporting for specific materials, systems and methods

We require information from authors about some types of materials, experimental systems and methods used in many studies. Here, indicate whether each material, system or method listed is relevant to your study. If you are not sure if a list item applies to your research, read the appropriate section before selecting a response.

Materials & experimental systems

n/a	Involved in the study
☐	☒ Antibodies
☒	☐ Eukaryotic cell lines
☒	☐ Palaeontology
☐	☒ Animals and other organisms
☒	☐ Human research participants
☒	☐ Clinical data

Methods

n/a	Involved in the study
☒	☐ ChIP-seq
☐	☒ Flow cytometry
☒	☐ MRI-based neuroimaging

Antibodies

Antibodies used

Antibodies used in this study are as follows:

Flow cytometry:
eBioscience:
Name / Clone name/ Catalog no. / Lot no. (most recent lot no.) / dilution factor or concentration
CD3 APC / 145-2C11 / 17-0031-82 / 4283668 / 1:100
CD4 eFluor 450 / RM4-5 / 48-0042-82 / E08484-1634 / 1:200
CD44 PE / IM7 / 12-0441-83 / E01240-1630 / 1:400

Harlan, custom order:
Anti-CD16/32 / 24G2 / 10ug/ml

Validation

All flow cytometry antibodies were validated by the manufacturer.

Animals and other organisms

Policy information about studies involving animals; ARRIVE guidelines recommended for reporting animal research

Laboratory animals

All mice were bred and maintained in specific pathogen-free conditions according to Home Office UK Animals (Scientific Procedures) Act 1986 unless otherwise stated and were used at 6-18 weeks of age. C57BL/6/J wild-type mice were bred at the MRC National Institute for Medical Research (NIMR) or The Francis Crick Institute unless otherwise stated. Ifnar1-/- originally provided by Matthew Albert (Institute Pasteur, France)1 and Ifngr1-/- 2on the C57BL/6 background were further inter-crossed to generate Ifnar1-/-Ifngr1-/- mice. All animal experiments were carried out in accordance with UK Home Office regulations unless otherwise stated, project licences: Toxoplasma gondii infection of Ifnr deficient mice, 80/2616 (The Francis Crick Institute); Influenza A infection, 70/7643 (MRC NIMR); Respiratory syncytial virus infection, 70/7554 (Imperial College London); Burkholderia pseudomallei infection, 70/6934 (London School of Hygiene and Tropical Medicine); Candida albicans infection, 70/8811 (MRC NIMR); House dust mite allergy, 70/7643 and P5AF488B4 (MRC NIMR and The Francis Crick Institute) and 70/7463 (Imperial College London); Plasmodium chabaudi chabaudi AS infection, 80/2358 and 70/8326 (MRC NIMR and The Francis Crick Institute); murine Cytomegalovirus infection, 30/2969 (Cardiff University); Listeria monocytogenes infection, 70/7643 (MRC NIRM) and were approved by the institutions' Ethical Review Panels unless otherwise stated. T. gondii infection experiments for module derivation, C57BL/6 mice were obtained from Taconic Biosciences and maintained at an American Association for the Accreditation of Laboratory Animal Care–accredited animal facility at NIAID and housed in accordance with the procedures outlined in the Guide for the Care and Use of Laboratory Animals under an animal study proposal approved by the NIAID Animal Care and Use Committee.

Wild animals

This study does not involve wild animals.

Field-collected samples

This study does not contain field-collected samples.

Ethics oversight

All mice were bred and maintained in specific pathogen-free conditions according to Home Office UK Animals (Scientific

Ethics oversight	Procedures) Act 1986 unless otherwise stated and were used at 6 to 18 weeks of age. All animal experiments were carried out in accordance with UK Home Office regulations unless otherwise stated, project licences: Toxoplasma gondii infection of wild type control mice, Ifnar-/-, Ifngr-/- and double Ifnar-/- x Ifngr-/- mice (for Figs. 5 – 8 and Supplementary Figs. 10 – 19) 80/2616 (at The Francis Crick Institute); Influenza A infection, 70/7643 (MRC NIMR); Respiratory syncytial virus infection, 70/7554 (Imperial College London); Burkholderia pseudomallei infection, 70/6934 (London School of Hygiene and Tropical Medicine); Candida albicans infection, 70/8811 (MRC NIMR); House dust mite allergy, 70/7643 (MRC NIMR), P5AF488B4 (The Francis Crick Institute) and 70/7463 (Imperial College London); Aspergillus fumigatus infection, 70/8811 (The Francis Crick Institute); Plasmodium chabaudi chabaudi AS infection, 80/2358 (MRC NIMR) and 70/8326 (The Francis Crick Institute); murine cytomegalovirus infection, 30/2969 (Cardiff University); Listeria monocytogenes infection, 70/7643 (MRC NIRM) and were approved by the institutions' Ethical Review Panels unless otherwise stated. C57BL/6 mice mice for T. gondii infection used for module derivation and further analysis (Figs. 1-4 and Supplementary Figs. 1, 4, 5, 6 and 7) were maintained and infected at an American Association for the Accreditation of Laboratory Animal Care–accredited animal facility at National Institute of Allergy and Infectious Diseases (NIAID) and housed in accordance with the procedures outlined in the Guide for the Care and Use of Laboratory Animals under an animal study proposal approved by the NIAID Animal Care and Use Committee.

Note that full information on the approval of the study protocol must also be provided in the manuscript.

Flow Cytometry

Plots

Confirm that:

- [ ] The axis labels state the marker and fluorochrome used (e.g. CD4-FITC).
- [ ] The axis scales are clearly visible. Include numbers along axes only for bottom left plot of group (a 'group' is an analysis of identical markers).
- [ ] All plots are contour plots with outliers or pseudocolor plots.
- [ ] A numerical value for number of cells or percentage (with statistics) is provided.

Methodology

Sample preparation	For fluorescence-activated cell sorting (FACS) of T cells, pooled blood from four or five individual HDM or PBS treated mice was collected into heparin sodium (Wockhardt) at 10 to 30 international units per ml of blood. Peripheral blood mononuclear cells were isolated by density separation with Lympholyte®-Mammal (Cedarlane). Lung CD4+ T cells were enriched by positive selection (Miltenyi Biotech) from a corresponding pool of lungs. Blood and lung cells were stained with CD3 (145-2C11) APC, CD4 (RM4-5) eFluor450 and CD44 (IM7) PE (all from eBioscience) and CD3+CD4+, CD3+CD4+CD44low and CD44high cells were sorted on MoFlo XDP (Beckman Coulter) and BD FACSAria™ Fusion (Beckton Dickinson) flow cytometers and 15,000 per population collected into TRI-Reagent LS (Sigma-Aldrich).
Instrument	Cells were sorted on BD FACSAria™ Fusion (Beckton Dickinson) and MoFlo XDP (Beckman Coulter) flow cytometers.
Software	Flow cytometry data were collected using the BD FACSDiva software version 8.0.1 on a FACSAria™ Fusion and Summit V5.4.0 software on a MoFlo XDP (Beckman Coulter).
Cell population abundance	Whole CD3+CD4+, naïve CD3+CD4+44low and memory CD3+CD4+CD44high T cell fractions were routinely sorted using purity mode setting to over 95% purity, as checked by flow cytometry.
Gating strategy	Lymphocytes were identified by cell size in FSC vs SSC, doublets were discriminated by FSC-width and SSC-width, and live (propidium idodide was used for live/dead discrimination) CD3+CD4+, CD3+CD4+CD44low and CD44high cells were FACS sorted from blood and lung single cell suspensions, the latter enriched for CD4+ T cells by MACS.

- [ ] Tick this box to confirm that a figure exemplifying the gating strategy is provided in the Supplementary Information.
